# Optimization of Antiproliferative Properties of Triimine Copper(II) Complexes

**DOI:** 10.1021/acs.jmedchem.4c01806

**Published:** 2024-11-04

**Authors:** Katarzyna Choroba, Bartosz Zowiślok, Sławomir Kula, Barbara Machura, Anna M. Maroń, Karol Erfurt, Cristiana Marques, Sandra Cordeiro, Pedro V. Baptista, Alexandra R. Fernandes

**Affiliations:** † Institute of Chemistry, University of Silesia, Szkolna 9, 40-006 Katowice, Poland; ‡ Department of Chemical Organic Technology and Petrochemistry, Silesian University of Technology, Krzywoustego 4, 44-100 Gliwice, Poland; § Associate Laboratory i4HB - Institute for Health and Bioeconomy, NOVA School of Science and Technology, NOVA University Lisbon, 2819-516 Caparica, Portugal; ∥ Departamento de Ciências da Vida, NOVA School of Science and Technology, UCIBIO, Campus de Caparica, 2829-516 Caparica, Portugal

## Abstract

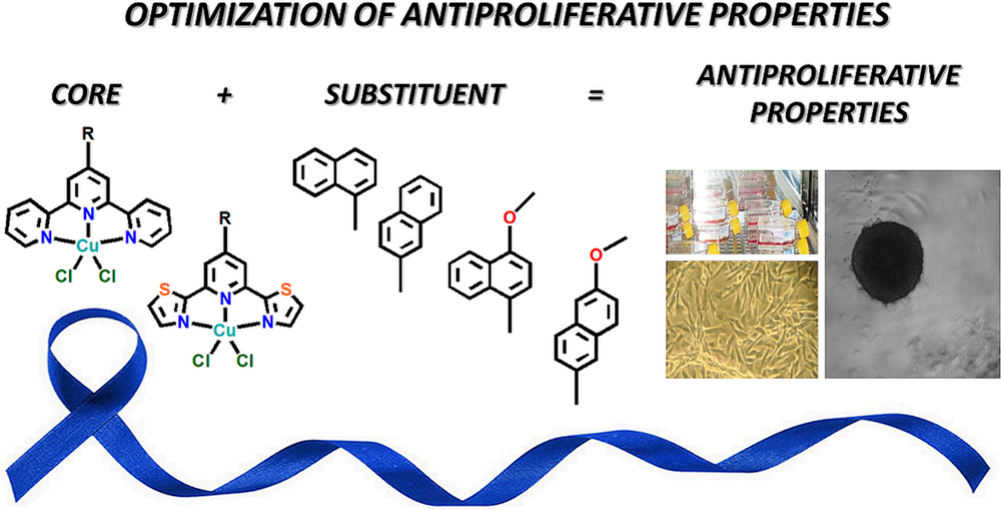

Cu(II) complexes with 2,2′:6′,2″-terpyridines (terpy) and 2,6-bis(thiazol-2-yl)pyridines (dtpy) with 1- or 2-naphtyl and methoxy-naphtyl were synthesized to elucidate the impact of the triimine core, naphtyl linking mode, and presence of methoxy groups on the antiproliferative activity of [CuCl_2_(L^*n*^)]. Their antiproliferative effect was analyzed in ovarian (A2780) and colorectal (HCT116) carcinomas and colorectal carcinoma resistant to doxorubicin (HCT116-DoxR) cell lines and in normal human fibroblasts. Among all complexes, the 1- and 2-naphtyl substituted terpy Cu(II) complexes (**Cu1a** and **Cu1b**) showed the strongest cytotoxicity, namely, in HCT116-DoxR 2Dcells and were also capable of inducing the loss of cell viability in 3D HCT116-DoxR spheroids. Their intracellular localization, capability to increase reactive oxygen species (ROS), and interaction with DNA (nonintercalative mode) trigger oxidative DNA cleavage leading to cell death by apoptosis and autophagy. **Cu1a** and **Cu1b** do not show in vivo toxicity in a chicken embryo and can interact with bovine serum albumin (BSA).

## Introduction

Cancer ranks second as the leading cause of death worldwide, behind cardiovascular diseases. Regarding global trends, however, there is a high probability that this statistical tendency will be reversed before 2030. Besides surgery, the main treatment of cancer is based on chemotherapy. Currently, almost half of chemotherapy patients receive cisplatin, which induces cytotoxic properties through covalent binding to nuclear DNA and the formation of Pt-DNA cross-links. Despite the widespread clinical use of cisplatin and its derivatives (carboplatin or oxaliplatin), platinum-based drugs have several drawbacks, including tumor cell resistance, low selectivity, and severe side effects such as emetogenesis, neurotoxicity, myelotoxicity or nephrotoxicity.^1,2^ Therefore, there is a strong need to design new biologically active agents with better therapeutic indices. In particular, an increasing scientific interest has been devoted to the development of non-platinum anticancer agents, especially those containing bioessential metal ions^3−9^ and to the search for coordination compounds operating via mechanisms distinct from those of cisplatin and its analogues.^10−13^

Cu(II) complexes with 2,2′:6′,2″-terpyridine (terpy) derivatives,^14−26^ which combine both aforementioned approaches, are among the most promising candidates. Unlike platinum, copper is an endogenous metal, and Cu(I) and Cu(II) ions play vital roles in metalloproteins and numerous physiological cellular processes.^27,28^ The Cu(II)/Cu(I) redox-couple participates in the generation of reactive oxygen species (ROS) by reacting with molecular oxygen or hydrogen peroxide.^29^ As a cofactor in angiogenesis,^30,31^ copper is essential in the formation of new blood vessels from the existing vasculature. Additionally, d–d transitions in the red wavelength range make Cu(II) complexes interesting regarding their potential applications in photodynamic therapy.^14^

Chelating terpy-like ligands, introduced into the copper coordination sphere, modulate the complex stability, solubility, lipophilicity, and reactivity, facilitate the complex transport through cell membranes, and promote noncovalent interactions with DNA via π-stacking, major groove, and electrostatic binding. Thanks to the highly efficient Kröhnke method,^32−34^ which allows for the introduction of various types of substituents into the central pyridine ring and the replacement of peripheral pyridines by other heterocycles in a one-pot reaction, the overall efficacy of Cu-based drugs can be widely tuned by combining copper coordination diversity and structural modifications of terpy-like ligands, as reviewed in refs (14,21,35,36) and summarized in Table S1. Moreover, 2,2′:6′,2″-terpyridine derivatives are known for their prominent antitumor activity.^37^

**Scheme 1:**
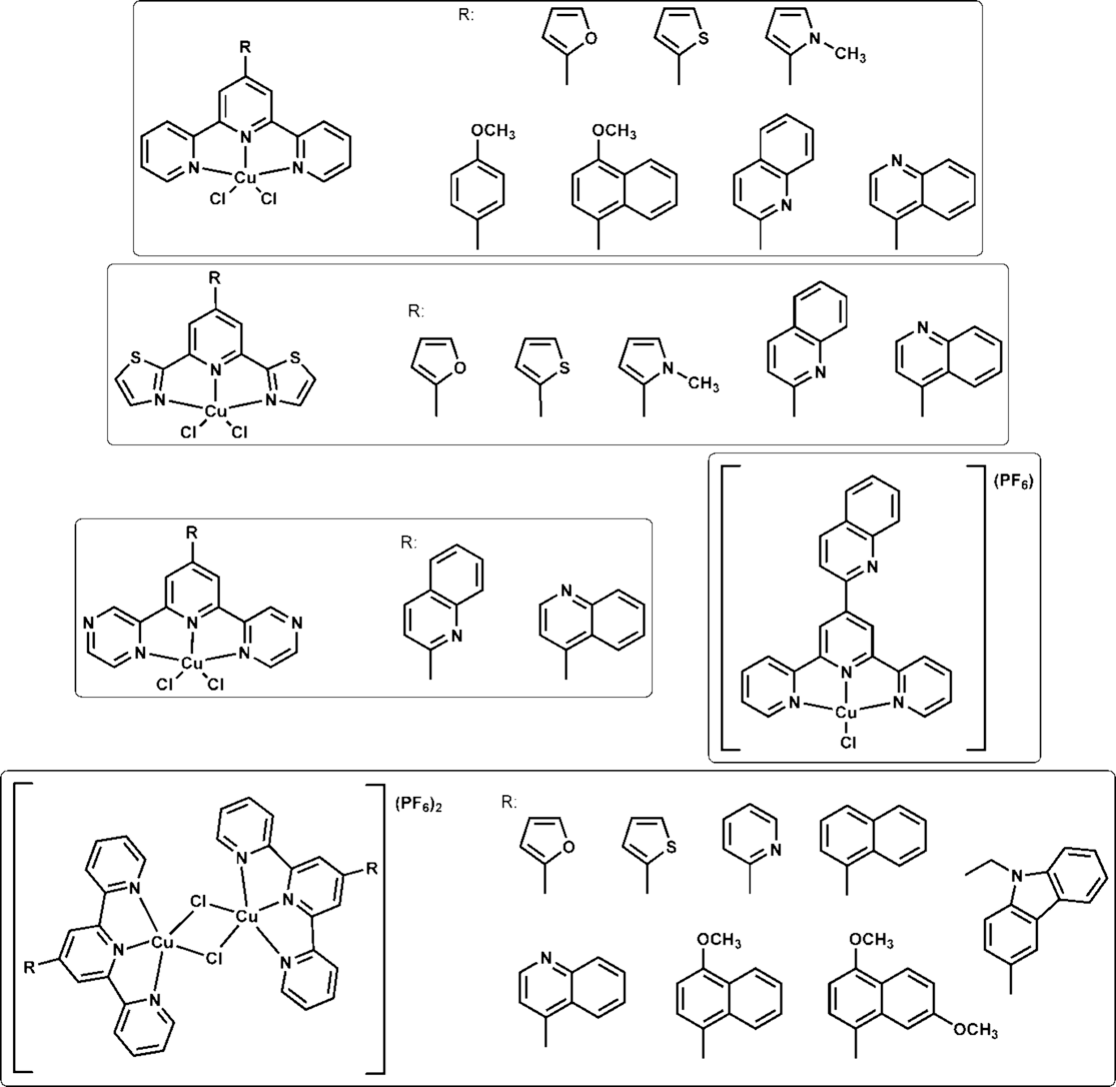
Copper(II) Compounds Discussed Previously in Our Study^22−24,38^

In our previous studies,^22−24,38^ we have preliminarily assessed the role of a substituent attached to the central pyridine ring of terpy and the triimine skeleton (terpy, 2,6-bis(thiazol-2-yl)pyridine (dtpy) and 2,6-di(pyrazin-2-yl)pyridine (dppy)) in determining the geometry and therapeutic indices of resulting triimine Cu(II) complexes. We demonstrated that enhanced antiproliferative activity against human colorectal (HCT116) and ovarian (A2780) carcinoma is induced by increasing substituent π-conjugation (4-methoxyphenyl and 4-methoxynaphtyl) and decreasing the dihedral angle between the substituent and triimine planes (2-quinolyl versus 4-quinolyl) (Scheme 1).^23,24^ Furthermore, five-coordinate Cu(II) complexes with a geometry closer to tetragonal pyramid were found to be more cytotoxic than those showing a distorted trigonal bipyramidal geometry, while square-planar [CuCl(2-quin-terpy)]^+^ (where 2-quin-terpy is 4′-(quinol-2-yl)-2,2′:6′,2″-terpyridine) was much more active than its square pyramidal analogue [CuCl_2_(2-quin-terpy)] toward HCT116 and A2780 cells.^23,38^ In turn, the replacement of terpy framework with more π-accepting thiazole groups in dtpy was beneficial for the specificity of Cu(II) complexes against the ovarian cancer-derived cell line (A2780).^22,23^ Terpyridine Cu(II) complexes with appended substituted phenyl groups, quinoline, or other heterocycles were found to have IC_50_values significantly lower than those of cisplatin,^15−26^ while the antiangiogenic effect was confirmed for [Cu(4′-(2-quin)-terpy)Cl]PF_6_.^38^

**Scheme 2:**
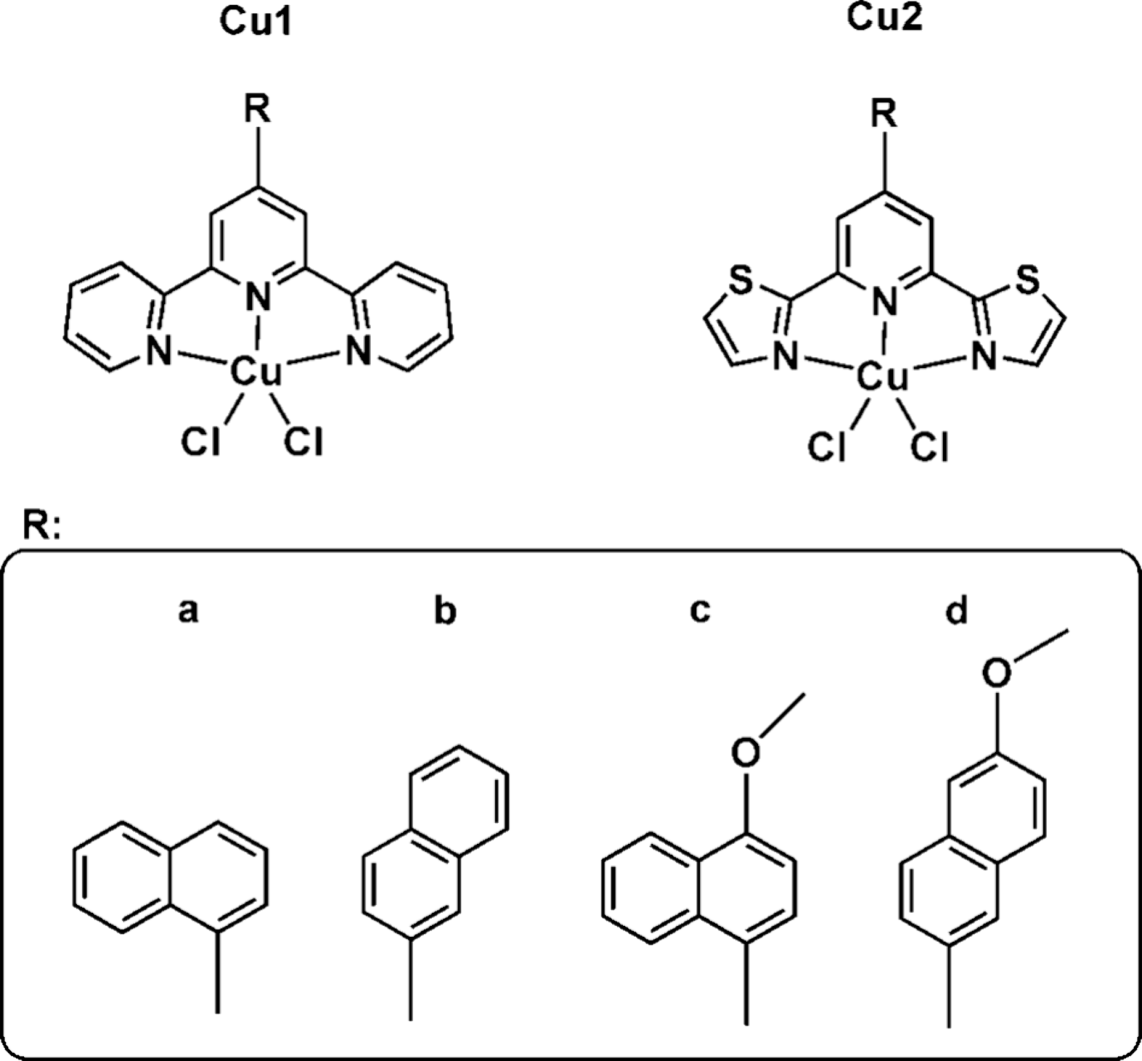
Schematic Representation of the Copper(II) Compounds Discussed in This Study

Continuing our research in this field, we present herein a series of new Cu(II) compounds with *n*-naphtyl (*n* = 1, 2) and metoxy-naphtyl substituted terpys and dtpys (**Cu1a–b**, **Cu1d**, and **Cu2a–d** in Scheme 2). Except for **Cu1c** which was previously reported^24^ and included in the current paper for comparative analysis, all presented compounds are designed for the first time. The choice of the pendant group attached to the triimine terpy and dtpy frameworks was based on our previous experience^22−24^ and a comprehensive literature survey of terpyridine Cu(II) complexes (Table S1), indicating the positive impact of π-extended aryl units and methoxy groups on the antiproliferative activity of Cu-based drugs. Furthermore, the complexes were designed to broaden the knowledge of structure-antiproliferative relationships in the series of five-coordinate Cu(II) complexes [Cu(NNN)Cl_2_]. More specifically, the impact of the triimine core (terpy, dtpy), naphtyl linking mode, and the dihedral angle between the substituent plane and planar triimine backbone, as well as the functional (methoxy) group introduced into the naphtyl ring, were explored.

The triimine backbone was demonstrated to be crucial regarding the stability of the resulting Cu(II) complexes in solution, while the decoration of the aryl substituents with methoxy groups resulted in a lowering of their therapeutic indexes toward colorectal cancer cell lines. Most importantly, two of the obtained compounds (**Cu1a** and **Cu1b**) were found to be highly promising, as they are characterized by a very good therapeutic window in HCT116 and HCT116-DoxR-resistant cells, with higher selectivity indexes relative to doxorubicin and cisplatin. Among the [Cu(NNN)Cl_2_] reported so far (Table S1), **Cu1a** and **Cu1b** display the highest cytotoxic effect toward colorectal cancer cell lines. To understand the biological activity of these systems, a wide range of in vitro and in vivo studies were performed, allowing us to determine their cellular mechanisms. Regarding the anticancer profiles of **Cu1a** and **Cu1b**, the results presented herein are relevant for making further progress in the design of efficient Cu-based drugs for colorectal cancer cells.

## Results and Discussion

### Synthesis and Molecular Structures

**Scheme 3:**
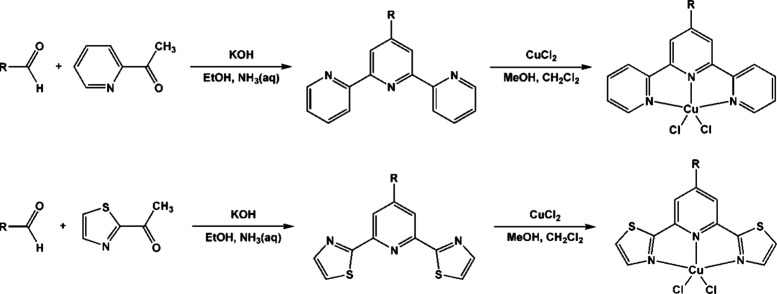
Synthesis of Ligands and Cu(II) Complexes

The synthesis of Cu(II) complexes was preceded via preparation of the triimine ligands in the Khrönke condensation between 2-acetylpyridine (**L1a, b, d**) or 2-acetylthiazole (**L2a**–**d**) and the aldehydes: 1-naphthaldehyde (**L1a, L2a**), 2-naphthaldehyde (**L1b, L2b**), 4-methoxy-1-naphthaldehyde (**L2c**), and 6-methoxy-2-naphthaldehyde (**L1d, L2d**)^24,39−42^ (see Scheme 3). The synthesis of Cu(II) complexes followed standard procedures described elsewhere.^22,23^ The reaction between CuCl_2_·2H_2_O in methanol and an equimolar amount of the appropriate ligand in methanol: dichloromethane mixture (1:1) resulted in the formation of green solids of **Cu1a–d** and **Cu2a–d** with good yields (75–86%).

**Figure 1:**
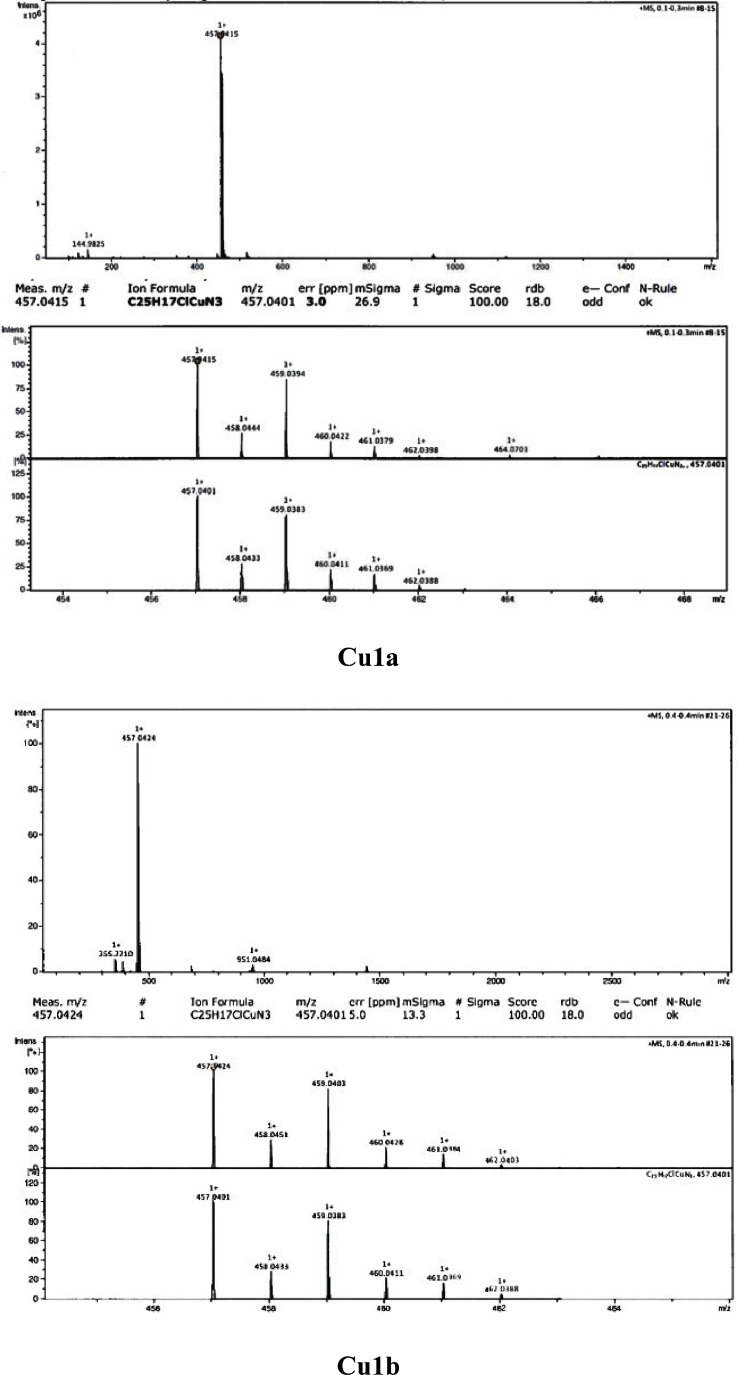
HR-MS spectra of **Cu1a** and **Cu1b**.

The successful tridentate coordination of the triimine ligand and formation of five-coordinate complexes with the general formula [CuCl_2_(L^*n*^)] were confirmed by elemental analysis, HR-MS spectrometry (Figures 1 and S1), FT-IR spectroscopy (Figure S2) and single crystal X-ray analysis (Tables S2–S8 and Figure S5). Additionally, the purity was evidenced by UPLC (Figures S3 and S4) using a PDA detector in the 190–400 nm wavelength range. Typically of [Cu(NNN)Cl_2_] systems,^16,17,19,23,25,43^ the mass spectrometry spectra with electrospray ionization (ESI-MS) exhibit *m*/*z* signals corresponding to the ion [CuCl(R-terpy)]^+^ [457.0415 (**Cu1a**), 457.0424 (**Cu1b**), 487.0513 (**Cu1c**), 487.0523 (**Cu1d**), 468.9540 (**Cu2a**), 468.9539 (**Cu2b**), 498.9628 (**Cu2c**), and 498.9630 (**Cu2d**)] with isotopic distributions typical of copper. In agreement with the coordination of R-terpy and R-dtpy to the Cu(II) ion, the characteristic ν_C=N_ and _C=C_ stretching modes in the FT-IR spectra of Cu(II) complexes shifted to higher wavenumbers compared to those of the free ligands.

**Figure 2:**
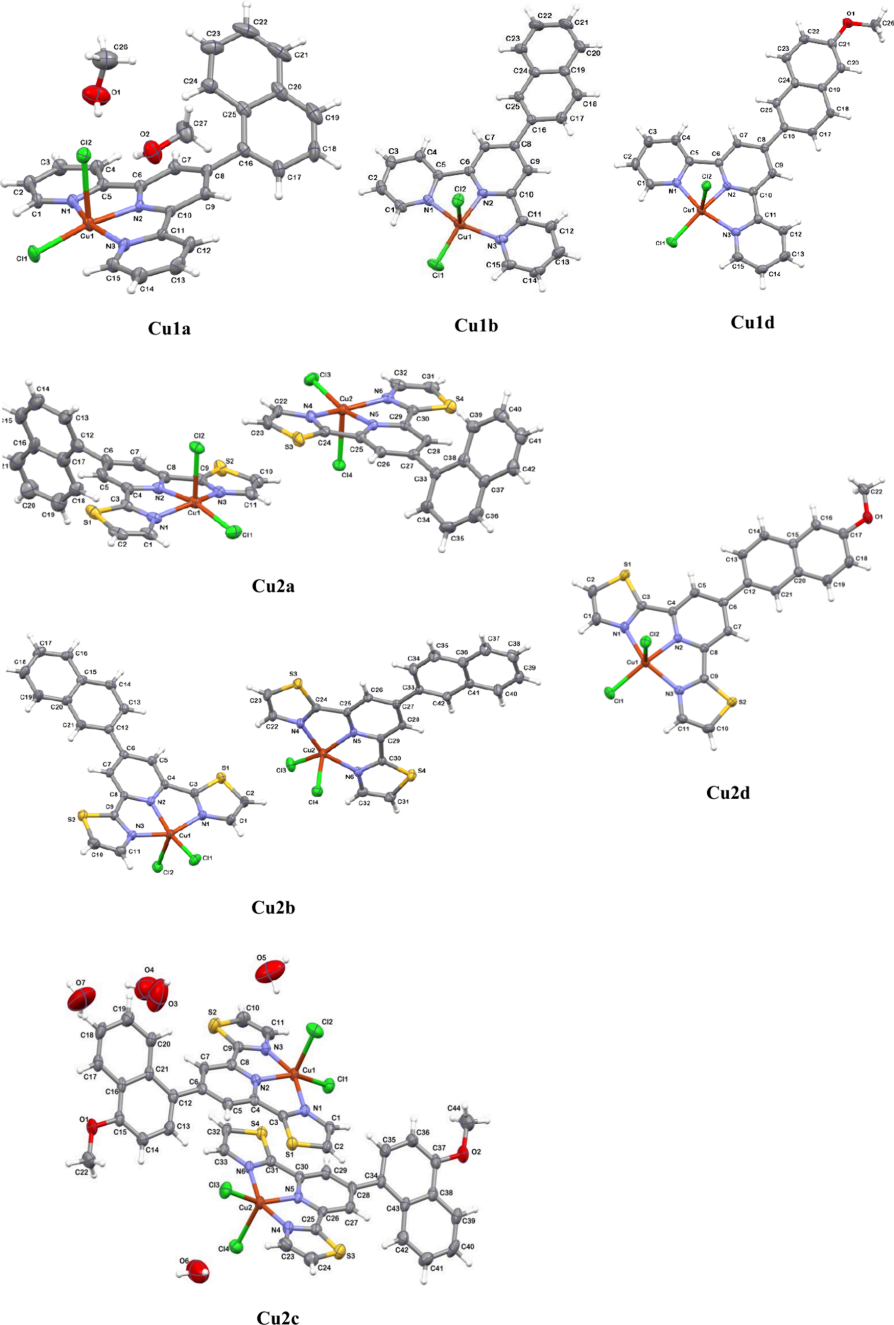
Perspective views showing the asymmetric units of the structures **Cu1a** (CCDC 2124050), **Cu1b** (CCDC 2150148), **Cu1d** (CCDC 2124051), **Cu2a** (CCDC 2124052), **Cu2b** (CCDC 2124054), **Cu2c** (CCDC 2361049) and **Cu2d** (CCDC 2124053), with the atom numbering. Displacement ellipsoids are drawn at the 50% probability level. Except for **Cu1a** and **Cu1b**, solvent molecules were removed from the electron density map, and they were modeled employing the OLEX2 solvent mask command.^44^

X-ray quality crystals of the newly reported Cu(II) complexes were obtained, following slow evaporation from MeOH solutions. The crystal structures of all complexes with the exception of **Cu1b**, consist of [CuCl_2_(L^*n*^)] and cocrystallized solvent molecules (H_2_O, MeOH). Except for **Cu1a**, however, it was not possible to satisfactorily model solvent molecules. Employing the OLEX2 solvent mask command,^44^ solvent molecules were removed from the electron density map, and they are not considered in the given chemical formula and other crystal data (Tables 1 and S2–S8; Figures 2 and S5). As expected, all Cu(II) complexes show a five-coordinate geometry around the metal ion, formed by three nitrogen atoms of substituted terpyor dtpy ligand and two chloride ions (Figure 2). In each case, the angular structural index τ^45^ and SHAPE measure *S*_Q_(*P*) parameters^46^ indicate that the coordination geometry around the Cu(II) ion is best described as square pyramidal (Table 1). Typical of square pyramidal Cu(II) complexes with planar terpy-like ligands,^47^ (i) the Cu–Cl apical bond [2.4359(16)–2.643(2) Å] is significantly elongated relative to the Cu–Cl basal one [2.2022(17)–2.2438(14) Å] in accordance with Jahn–Teller distortion,^48^ (ii) the Cu–N_central pyridine_ bond length [1.9390(19)–1.978(4) Å] is noticeably shorter compared to the two outer Cu–N bonds[2.025(5)–2.076(4) Å], and (iii) bite angles N–Cu–N, formed after κ^3^N-coordination of the R-terpy/R-dtpy ligand, are smaller than ideal value 90° [77.94(16)–80.0(2)°] (Table S3 in ESI).

**Table 1:** Distortion of the Coordination Sphere of Cu Ions from Ideal Five-Vertexes Polyhedral (Square-Pyramid (SPY) and Trigonal Bipyramid (TBY)) Expressed by Angular Structural Index Parameter τ^45^ and Calculated Using the SHAPE Program^46^

Cu(II) complexes		SHAPE v2.0 continuous shape measures		τ_5_
		TBPY-5	SPY-5	
**Cu1a**		6.539	1.971	0.178
**Cu1b**		6.080	1.589	0.115
**Cu1c**(24)		4.987	1.589	0.085
**Cu1d**		6.682	1.835	0.216
**Cu2a**	molecule 1	6.665	1.564	0.158
	molecule 2	6.571	1.475	0.165
**Cu2b**	molecule 1	5.783	1.654	0.089
	molecule 2	5.240	1.673	0.046
**Cu2c**	molecule 1	5.335	1.530	0.025
	molecule 2	5.228	1.500	0.002
**Cu2d**		6.151	1.631	0.126

Continuous shape measures are calculated with the use of SHAPE v2.0^46,49^ employing eq 1:
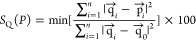
1

, *N* vectors with the 3*N*Cartesian coordinates of the problem structure Q; , the coordinates of the ideal polyhedron *P*; , the position vector of the geometric center that is chosen to be the same for the two polyhedral.

Parameter τ_5_^45^ is calculated with use of the eq 2:
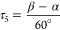
2α and β–two greatest valence angles, α < β

In each case, the triimine unit (terpy or dtpy) forms a planar framework, with the angles between the mean planes of the central pyridine and peripheral aromatic rings below 8.5° value (Table 2). Regarding the twist of the substituent plane relative to the central pyridine, significant differences can be observed between Cu(II) complexes bearing 1-naphtyl (**Cu1a** and **Cu2a**) and 2-naphtyl substituents (**Cu1b**, **Cu1d, Cu2b** and **Cu2d**), which are attributed to stronger inter-ring H···H repulsive interactions in the case of complexes with 1-naphtyl substituted terpy and dtpy ligands. While the pendant substituents of **Cu1b, Cu1d**, **Cu2b**, and **Cu2d** remainnearly coplanar with the central pyridine plane (1.36–7.70°), the dihedral angle between the central pyridine and appended group falls in the range 35–70° for **Cu1a**, **Cu2a**, and **Cu2c**. The replacement of the terpy by dtpy core results in an increased twist of the aryl substituent relative to the triimine core^22,23,25,26^ (Table S8).

**Table 2:** Dihedral Angles between Mean Planes of Selected Aromatic Rings in the Studied Cu(II) Structures

Cu(II) complexes		dihedral angle py_central_–R	dihedral angles py_central_–py_distal_
**Cu1a**		48.24	5.04
			5.84
**Cu1b**		3.96	5
			5.77
**Cu1c**(24)		30.02	7.02
			8.37
**Cu1d**		1.55	4.63
			6.99
**Cu2a**	molecule 1	58.94	1.86
			5.5
	molecule 2	69.94	1.89
			6.52
**Cu2b**	molecule 1	7.7	7.53
			8.39
	molecule 2	1.36	3.15
			5.82
**Cu2c**	molecule 1	39.14	6.27
			7.64
	molecule 2	36.9	4.75
			7.6
**Cu2d**		5.45	1.28
			7.88

### UV–Vis Spectra and Stability Studies in Solution

The stability of **Cu1a-d** and **Cu2a-d** was assessed using a spectrophotometric method. Absorption spectra of the Cu(II) complexes were collected every 4 h for 72 h in DMSO and phosphate-buffered saline (PBS) solutions (*c* = 25 μM) (see Figure S6 and Table S9 in ESI). The diluted solutions in a water-based medium were prepared from concentrated (50 mM) stock solutions in DMSO. All obtained terpy Cu(II) complexes demonstrated no spectral changes in DMSO and PBS solutions over 72 h, indicating that compounds **Cu1a–d** are stable under these conditions (Figure S6). In contrast, Cu(II) complexes with dtpy-based ligands (**Cu2a–d**) decomposed within a few minutes in DMSO, showing UV–vis spectra almost overlapping with those of the free ligands (Figure S7). The higher solution stability of **Cu2a–d** was observed in ethanol and acetonitrile (Figure S8a,b). In PBS, compounds **Cu2a–d** tended to aggregate rapidly due to the release of the organic ligand, which is insoluble in water.

The electronic absorption spectra of the terpy Cu(II) complexes (*c* = 25 μM) are dominated by one or two strong bands of intraligand (IL) transitions (π → π* and *n →*π*) in the range 250–410 nm,^39,41,42^ which are significantly red-shifted relative to the free ligand (Figure S7). In solutions of higher concentration (*c* = 1 mM), the UV–vis spectra of these systems also show a weak and broad band in the wavelength range 550–1000 nm, attributed to metal-centered transitions within the copper(II) ion (*d*_*xy*_ → *d*_*x*_^2^_–*y*_^2^, *d*_*yz*_,*d*_*xz*_ → *d*_*x*_^2^_–*y*_^2^and *d*_*z*_^2^ → *d*_*x*_^2^_–*y*_^2^)^50−52^ (see Inset Plots in Figures S6 and S7 in ESI).

### Cell Viability Assays in 2D Models

The antiproliferative effect of the Cu(II) complexes in tumor and healthy cell lines was assessed through the MTS assay, a colorimetric method that is widely used to evaluate cellular viability, specifically mitochondrial viability.^53,54^ Cellular viability was determined after 48 h exposure to a concentration range (0.1–50 μM) of Cu(II) complexes in three tumor cell lines. The chosen cell lines aim to provide a deeper understanding of the complexes’ efficacy in two different types of cancer cell lines, specifically colorectal carcinoma (HCT116), doxorubicin (Dox) resistant colorectal carcinoma (HCT116-DoxR), and ovarian carcinoma (A2780) cell lines. Additionally, the viability of healthy primary dermal fibroblasts was performed as a comparison of the complexes’ antiproliferative effects in normal cells. This multifaceted approach was adopted to identify the most selective and promising Cu(II) complexes in a particular tumor cell line before further biological studies.

Cell viabilities in HCT116, HCT116-DoxR, and A2780 for Cu(II) complexes are depicted in Figures S9–S11. A reduction of cell viability is observed as the concentration of Cu(II) complexes **Cu1a**, **Cu1b**, **Cu1d**, and **Cu2c** increases (Figures S9–S11). On the other hand, complexes **Cu2a**, **Cu2b**, and **Cu2d** did not exhibit any significant antiproliferative effect for the tested human cells, as there is no significant reduction in cell viability up to 50 μM of Cu(II) complexes (Figures S9–S11). This lack of antiproliferative activity observed for Cu(II) naphtyl-substituted dtpy complexes could be related to their poor stability and decomposition in aqueous-based media (PBS) observed in Figure S6. From the cell viability data, the relative IC_50_ values (concentration at which 50% cell viability is achieved) for each Cu(II) complex in the respective cell line were calculated (Table 3).^54,55^ When looking at the cell viability in HCT116 and A2780 tumor cells, it is possible to see that the new complexes **Cu1a**, **Cu1b**, and **Cu1d** are more cytotoxic to tumor cells compared to the previously published complex **Cu1c**,^24^ indicating that these new substitutions favor the tumor cell cytotoxicity (Table 3). Considering this result, **Cu1c** was not tested in the additional colorectal cancer-resistant cell line (HCT116-DoxR). Comparing cell viability values and their respective IC_50_ (Table 3) considering the different tumor cell lines, the HCT116-DoxR cell line stands out as the cell line where most complexes exhibited higher antiproliferative activities (lower IC_50_), following the order: **Cu1a**> **Cu1b**> **Cu1d**> **Cu2c** with 1-naphtyl-substituted terpy Cu(II) complex(**Cu1a**) showing the strongest cytotoxicity when compared to 2-naphtyl-substituted terpy complex (**Cu1b**).

**Table 3:** Relative IC_50_ Values and SI (Selectivity Index, SI) of Cu(II) Complexes in HCT116, HCT116-DoxR, A2780 Tumor Cell Lines and in Normal Primary Dermal Fibroblasts after 48 h of Exposure; These Values Correspond to the Mean + SEM of Atleast Three Independent Assays

	IC_50_(μM)				SI		
complexes	HCT116	HCT116-DoxR	A2780	fibroblasts	HCT116	HCT116-DoxR	A2780
**Cu1a**	0.28 ± 0.03	0.24 ± 0.02	0.53 ± 0.07	9.26 ± 0.90	33.1	**38.6**	17.5
**Cu1b**	0.21 ± 0.05	0.29 ± 0.02	0.57 ± 0.06	13.30 ± 1.00	63.3	**45.9**	23.3
**Cu1c**(24)	0.40 ± 0.06		0.95 ± 0.05	32.78 ± 0.06	82		34.5
**Cu1d**	0.36 ± 0.04	0.31 ± 0.01	0.48 ± 0.08	6.42 ± 0.50	17.8	20.7	13.4
**Cu2a**	>50	>50		>50			
**Cu2b**	>50	>50		>50			
**Cu2c**	22.25 ± 0.20	15.22 ± 0.05	18.38 ± 0.73	15.58 ± 1.10	0.7	1.0	0.8
**Cu2d**	>50	>50		>50			
**Doxorubicin**	0.50 ± 0.10	>6	0.10 ± 0.04	12.10 ± 0.20	24.2	2.0	121.0
**Cisplatin**	15.60 ± 5.3		1.90 ± 0.20	8.80 ± 2.90	0.6		4.6

Moreover, by examining Table 3 and Figure S11, it can be observed that all tested complexes show lower antiproliferative activities in the A2780 cell line compared to other colorectal carcinoma cell lines.

**Figure 3:**
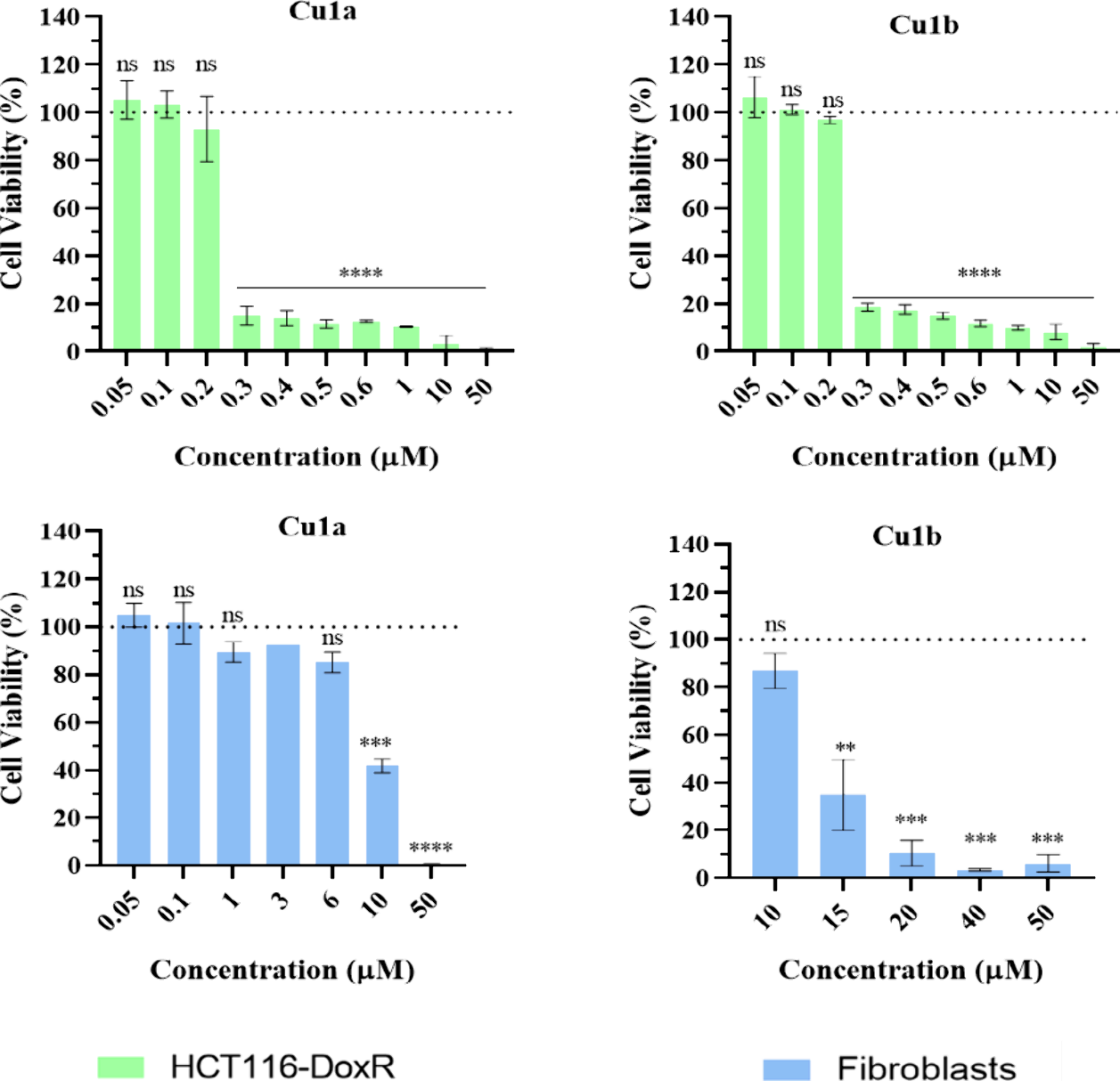
Cell viability of HCT116-DoxR and Fibroblasts cell line after 48 h exposure to different concentrations of **Cu1a** and **Cu1b** complexes. 0.1% (v/v) DMSO was used as the vehicle control. The presented values represent the mean ± SEM of two independent biological assays, and statistical significance was evaluated concerning the DMSO control using the T-Student and One-Way ANOVA methods (* *p*-value ≤0.05; ** *p*-value ≤0.005; *** *p*-value ≤0.0005; **** *p*-value ≤0.0001).

In the HCT116-DoxR cell line, complexes **Cu1a** and **Cu1b**, stand out as the most promising ones due to their lower IC_50_ (0.24 and 0.29 μM, respectively, i.e., higher cytotoxicity) (Table 3 and Figure 3), even when compared to the sensitive parental HCT116 cell line. These results are particularly relevant as this cell line mimics the acquired resistance in many colorectal cancer patients via P-glycoprotein (Pg-P) activation, a drug efflux transporter that is a key effector of Dox resistance.^56^ Complex **Cu1d** proved to be the third more cytotoxic in both HCT116 sensitive (Figure S9) and HCT116-DoxR (Figure S10), with IC_50_ values between 0.3 and 0.4 μM. Complex **Cu2c** showed the lowest cytotoxicity among Cu(II) complexes with antiproliferative activity, presenting an IC_50_ of 15.22 μM in HCT116-DoxR and 22.25 μM in HCT116 cell lines (Table 3).

In the identification of new metal complexes for cancer treatment, it is crucial to consider the impact that these molecules may have on healthy cells. Therefore, an MTS assay was also conducted on a lineage of healthy human cells, primary fibroblasts (Figures S12 and 3). These cells play a crucial role in the tumor microenvironment, making their study fundamental.^57^ Cell viability values and IC_50_ were determined in primary fibroblasts, and selectivity index (SI) values (ratio between IC_50_ of fibroblasts and the IC_50_ in each tumor cell line), a parameter that allows comparing the specificity of a drug toward normal versus tumor cells, were calculated (Table 3).

Complex **Cu2c** is more cytotoxic in normal fibroblasts compared to tumor cell lines (SI ≤ 1), which is associated with its low stability (Figure S6), is a good indication for not considering it in additional biological studies.

In the A2780 cell line, most complexes showed lower SI values compared to the other two colorectal carcinoma cell lines (Table 3). It is noteworthy that the two most promising complexes (**Cu1a** and **Cu1b**) still have higher SI values when compared to the other complexes, and with the two antitumor drugs, doxorubicin, and cisplatin (Table 3 and Figure S13), emerging again as the most promising, particularly in HCT116-DoxR cells (Figure 3 and Table 3), indicating a good therapeutic window, particularly in this resistant cell line. Although both complexes also showed very good SI values in HCT116 or A2780 cell lines, their impact in HCT116-DoxR cells, a model of acquired resistance of many cancer patients, is highly relevant, and this cell line was selected for additional biological studies.

To understand whether the cytotoxicity of the complexes is attributed to their respective ligands, it is important to evaluate their cytotoxicity in HCT116-DoxR (the selected cell line). The IC_50_ values are detailed in Table 4.

**Table 4:** Relative IC_50_ Values of Each Ligand in the HCT116-DoxR Cell Line after 48 h of Exposure^a^

	IC_50_(μM)
Ligands	HCT116-DoxR
**L1a**	∼0.18
**L1b**	0.24 ± 0.40
**L1d**	0.20 ± 0.05
**L2a**	>50
**L2b**	>50
**L2c**	∼11.50
**L2d**	∼14.65

a These values correspond to the mean + SEM of at least two independent assays.

Interestingly, viability results in Figure S14 show that the high antiproliferative effects of Cu(II) complexes **Cu1a, Cu1b**and **Cu1d** in HCT116-DoxR cell line, is mostly due to the cytotoxic action of their ligands **L1a, L1b**, and **L1d** respectively (Tables 3 and 4). Indeed, these three ligands show slightly higher cytotoxicities compared to their respective Cu(II) complexes (Tables 3 and 4). As expected, ligands **L2a–d,** shared by complexes **Cu2a–d,** showed none or lower antiproliferative activity, which may explain the low cytotoxic effect observed for the complexes (Tables 3 and 4). Since these complexes have been shown to decompose when in solution, once again, demonstrating a good correlation between the ligands cytotoxicity and solubility with their Cu(II) complexes antiproliferative potential.

### Cell Viability Assays in 3D Tumor Spheroids

**Figure 4:**
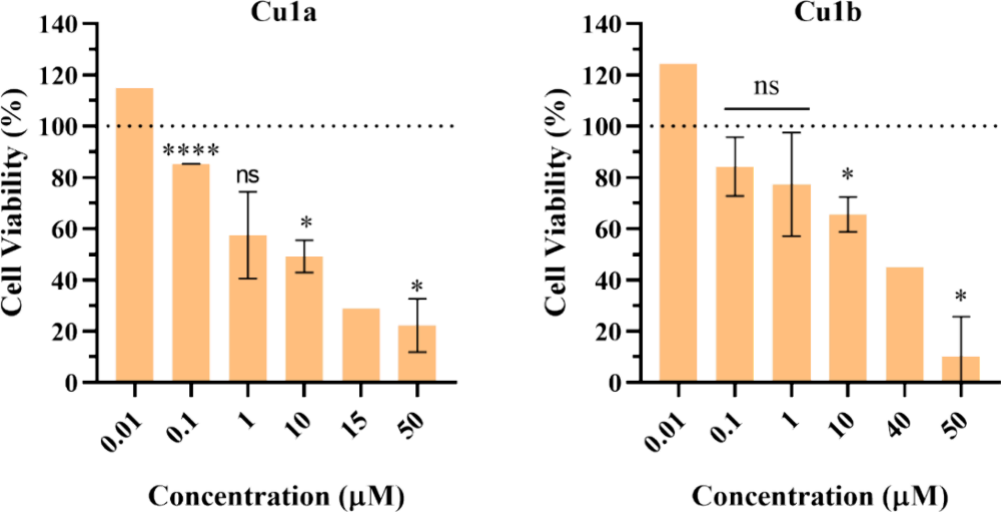
Cell viability of HCT116-DoxR 3D spheroids after 48 h of exposure to different concentrations of **Cu1a** and **Cu1b** complexes. 0.1% (v/v) DMSO was used as the vehicle control. The presented values represent the mean ± SEM of two independent biological assays, and statistical significance was evaluated concerning the DMSO control using the T-Student and One-Way ANOVA methods (* *p*-value ≤0.05; **** *p*-value ≤0.0001).

The research on tumor microenvironment (TME) has recently been gaining attention due to its important role in tumor growth, progression, and response to therapy.^57−60^ Because of this, the development of 3D cancer models that mimic the interactions in the TME and the tumor structure and complexity is of great relevance to cancer research and drug development. In this regard, the cytotoxicity of the two most promising Cu(II) complexes, **Cu1a** and **Cu1b**, was also evaluated in HCT116-DoxR 3D spheroid models to compare the influence of a more complex tumor microenvironment on their antiproliferative potential. The MTS assay was performed inHCT116-DoxR 3D spheroids with 6 days of growth (Figure S15 in ESI), followed by exposure to the complexes for 48 h. The results of cell viabilities and respective relative IC_50_ values calculated are provided in Figure 4 and Table 5, respectively.

**Table 5:** Relative IC_50_ Values of Complexes **Cu1a** and **Cu1b** in 2D HCT116-DoxR and in 3D Spheroids after 48 h of Exposure^a^

	IC_50_(μM)	
Cu(II) complexes	HCT116-DoxR 2D	HCT116-DoxR 3D spheroids
**Cu1a**	0.24 ± 0.02	10.10 ± 0.22
**Cu1b**	0.29 ± 0.02	33.86 ± 0.40

a These values correspond to the mean + SEM of at least three independent assays.

As expected, due to the complexity and density of the 3D models, drug penetration in HC116-DoxR 3D structures is more complex compared to a monolayer 2D culture, and in this regard, the IC_50_ concentrations are expected to be higher, which is exactly what is observed in the viability assay (Table 5). Specifically, the IC_50_ values for complexes **Cu1a** and **Cu1b** were ∼50× and 136×, respectively, higher in the 3D tumor model of HCT116-DoxR cells compared to the values obtained in 2D models (Table 5). Nevertheless, the cytotoxicity order is the same as previously, **Cu1a** ≫ **Cu1b**. This increase in IC_50_ concentrations in 3D spheroids follows cellular complexity increase, which is a common phenomenon in toxicology and pharmacology studies.^61^ This suggests that spheroids require a higher concentration of metal complexes to achieve the same biological effect. This difference is mainly attributed to the complexity of solid tumors, which present an associated gradient and a slower and irregular diffusion process, leading to a differential cellular response.^60,62,63^ Thus, the concentration used for spheroids will be more similar to the concentration used in in vivo assays.^64^ However, it is important to note that 3D spheroids also have some disadvantages, such as the lack of standardized and simplified methods compared to 2D models. 2D models have the advantage of extensive literature and a variety of techniques and technologies available for faster and more effective analyses.^63^ For this reason, subsequent assays were performed in 2D models to balance result representativeness with the practicality and resource availability for the research.

**Figure 5:**
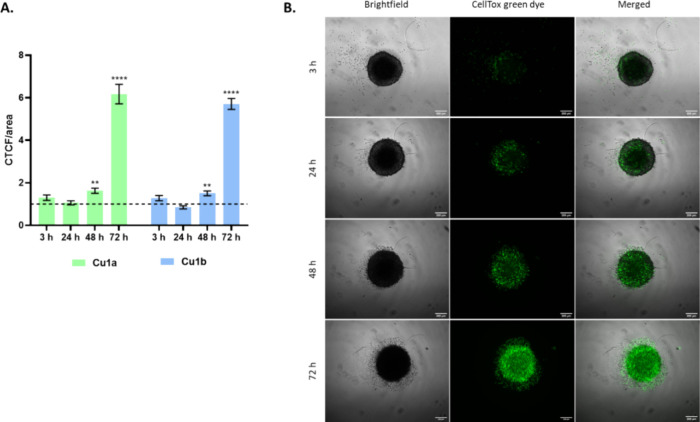
Cell viability in HCT116-DoxR 3D spheroids after exposure to DMSO (vehicle control) or 4.8 or 5.8 μM of complexes **Cu1a** and **Cu1b**, respectively, and incubation with CellTox Green dye for 3, 24, 48, and 72 h. (A) CTCF/area values represent as the mean ± SEM of three independent biological assays (each with at least 3 spheroids), and statistical significance was evaluated concerning the DMSO control (dashed horizontal black line in Figure) using the T-Student and One-Way ANOVA methods (** *p*-value ≤0.05; **** *p*-value ≤0.0001). (B) Representative images of fluorescence microscopy of HCT116-DoxR spheroids during the different times of exposure to complex **Cu1a**(see also Figure S16).

To prove that the spheroid’s structure is indeed being compromised and the complexes indeed induce the loss of cell viability in HCT116-DoxR 3D spheroids, the CellToxGreen cytotoxicity assay was performed in 3D spheroids with 6 days of growth (see Supplementary Figure S15 and Video S15), followed by their exposure to the complexes for 72 h. The results represented in Figures 5 and S16 show the increase of cell death (increase of green color) after 3, 24, 48, and 72 h of exposure to DMSO (vehicle control) or 4.8 or 5.8 μM of complexes **Cu1a** and **Cu1b**, respectively. Moreover, over time a detachment of death cells from the spheroids is observed (with green cells in the periphery of the outer region of the spheroids more clearly visualized at 72 h of exposure; Figure 5B and Supplementary Figure S16B,C).

As demonstrated in Figures 5 and S16, the cell viability/integrity of the spheroids is maintained until 24 h, after that the cells in the periphery of the 3D spheroids start to dye (green staining due to binding of CellTox green dye to cellular DNA) and the spheroid structure starts to fall apart (visualization of green death cells around the spheroid, Figure 5B and Supplementary Figure S16B,C) clearly highly relevant after 72 h of exposure to these concentrations of Cu(II) complexes. Additionally, only after 72 h of exposure to both complexes can we see green staining of the whole 3D spheroids, indicating the time dependence of complex diffusion within the tumor spheroid to promote cellular death (Figure 5B and SupplementaryFigure S16B,C). These results corroborate the obtained results for MTS (Figure 4) and the need for a higher concentration of Cu(II) complexes (IC_50_) to reduce 50% of cell viability at 48 h (MTS data in Table 5) and help understand which layer of the spheroids the complexes affect mostly over time.

### Evaluation of Complex Internalization and Subcellular Localization Using Inductively Coupled Plasma-Atomic Emission Spectroscopy (ICP-AES)

To validate whether the observed in vitro cytotoxicity of complexes **Cu1a** and **Cu1b** in HCT116-DoxR cells could be associated with differences in their internalization, the % of copper in the supernatant and in the cellular fraction was analyzed by Inductively Coupled Plasma-Atomic Emission Spectroscopy (ICP-AES), as done by others.^65^ To ensure significant results, due to the low IC_50_ values of the complexes and the ICP-AESlow limit of detection for copper, 10× the IC_50_ concentration of each complex was used. In this regard, HCT116-DoxR cells were exposed to 10× the IC_50_concentration of complexes **Cu1a** and **Cu1b** for 3 and 6 h of incubation at 37 °C. As shown inTable 6, all complexes were internalized after 3h of incubation, with **Cu1a** exhibiting a higher internalization rate (∼81%) compared to **Cu1b** (∼65%), which is in line with their cellular cytotoxicity (**Cu1a**> **Cu1b**) and similar to what has been described by others.^38^ Interestingly, after 6 h of incubation, there is a slight increase in the % of copper in the cellular fraction for both complexes (Table 6). This suggests that both complexes are mostly internalized in the first 3 h of exposure to HCT116-DoxR cells, reaching a saturation, which is in line with an active process.

**Table 6:** Percentage (%) of Copper in the Cellular Fractions after 3 and 6 h of Exposure of HCT116-DoxR Cells to Complexes **Cu1a** and **Cu1b** at 10× IC_50_ Concentrations

Cu(II) Complexes	10× IC_50_(μM)	percentage of internalization (%)	
		3 h	6 h
**Cu1a**	2.4	81.3	88.5
**Cu1b**	2.9	65.0	79.4

This internalization time is in line with the time needed for other complexes to internalize cells.^38,63,65^ To better understand where the complexes have a tendency to accumulate, the Abcam Standard cell fractionation kit (ab109719) was used to obtain the cytosolic, mitochondrial (which also includes the membrane and the endoplasmic reticulum), and nuclear (including the cytoskeleton and the Golgi complex) fractions after exposure of HCT116-DoxR cells to the complexes **Cu1a** and **Cu1b** for 6 h. Table 7 shows that the complexes are mostly detected in the cytosol (57.2 and 64.6% for complexes **Cu1a** and **Cu1b**, respectively), being also able to accumulate in the mitochondria, membrane, and endoplasmic reticulum. Despite a lower %, the complexes are also able to reach the nucleus (9.5 and 5.9% for complexes **Cu1a** and **Cu1b**, respectively).

**Table 7:** Percentage (%) of Internalization of Copper after 6 h Exposure of HCT116-DoxR Cells to Complexes **Cu1a** and **Cu1b** at 10× IC_50_ Concentrations and Respective Subcellular Fractionation^a^

Cu(II) complexes	10× IC_50_(μM)	percentage of internalization (%)		
		nucleus, cytoskeleton and golgi complex	mitochondria, membrane and endoplasmic reticulum	cytosol
**Cu1a**	2.4	9.5	29.0	57.2
**Cu1b**	2.9	5.9	26.8	64.6

a For this analysis, an Abcam standard cell fractionation kit (ab109719) was used.

### Stability Measurements

Despite that no solubility issues were observed in phosphate saline solution for **Cu1a** and **Cu1b** (at 25 μM), it is important to assess their solubility in biological media. Moreover, considering their higher IC_50_ values in 3D models (values between 10 and 33 μM, Table 5), complex solubility/stability was also evaluated through UV–visible spectroscopy over the first 3h of incubation at 37 °C (time for complexes internalization (Table 6)) (see Figure S17 in ESI). The complexes (powder form) were first dissolved in 100% (v/v) DMSO and subsequently in RPMI culture medium without *phenol red* and fetal bovine serum (FBS), at a final concentration of 50 μM. Absorption spectra were recorded in the wavelength range of 220–800 nm. As previously, high-energy absorption bands corresponding to π → π* and n → π* transitions with peaks between 230 and 380 nm ranges were observed, which may correspond to the aromatic rings of terpyridine.^24,38^ Regarding complex **Cu1a**, three bands in the region of 230–270 nm, 270–300 nm, and 300–360 nm were identified (Figure S17) for 0 and 3 h with the maintenance of solubility, which is a positive result. Regarding complex **Cu1b**, three absorbance bands are also evident, at 230–260 nm, 260–300 nm, and at 310–360 nm (Figure S17) for 0 and 3 h but a slight reduction of its solubility from 0 to 3 h of incubation.

However, this is not an issue for our biological assays as the concentrations used in all biological assays were 100× lower than 50 μM. However, when considering future in vivo experiments, the use of higher concentrations must be considered.

To ensure maximum solubility during biological assays, all solutions used were freshly prepared and homogenized well before each assay.

### Evaluation of Cell Death by Apoptosis

To understand the mechanism by which HCT116-DoxR cells lose their viability when exposed to the complexes **Cu1a** and **Cu1b**, particularly the type of cell death being induced, we began by studying one of the most common forms of programmed cell death in epithelial cells, apoptosis.^66^ Initially, a quantitative assay with double staining using annexin V-FITC (FITC, fluorescein isothiocyanate) and propidium iodide (PI) through flow cytometry was performed.^24^ Annexin V-FITC, a probe with an affinity for the ionic phospholipid phosphatidylserine (PS), predominantly located on the inner side of the plasma membrane in healthy cells, undergoes translocation to the outer plasma membrane during the early stages of apoptosis.^61,67^ In contrast, PI is a DNA intercalating agent that marks cells with compromised plasma membrane (usually in the late stages of apoptosis or in necrosis), emitting red fluorescence upon interaction with nucleic acids.^54,68^ This assay allows for distinguishing cells in four different conditions: viable cells (FITC–, PI−), early apoptosis (FITC+, PI−), late apoptosis (FITC+, PI+), and necrosis (FITC–, PI+).

**Figure 6:**
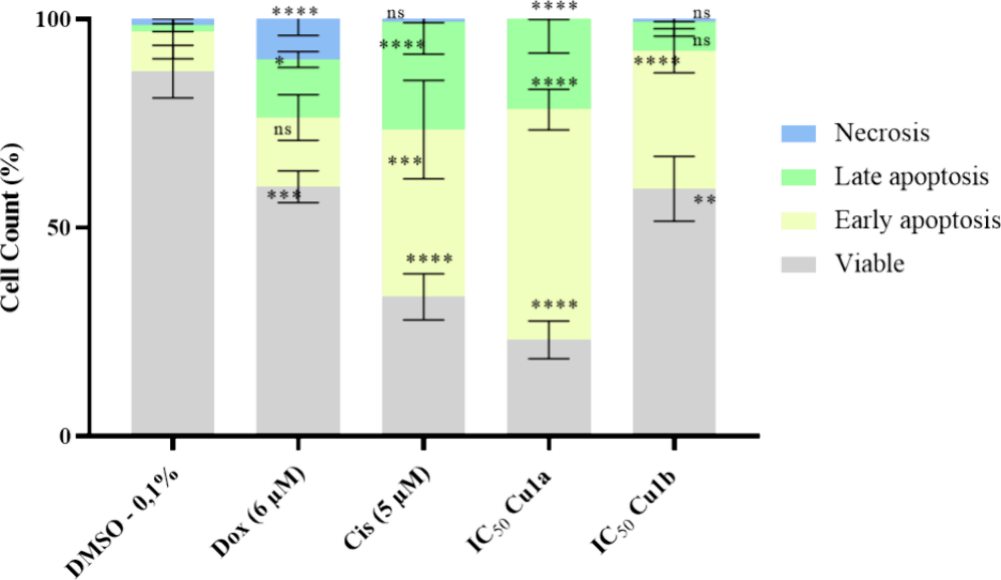
Quantification of viable HCT116-DoxR tumor cells, in early apoptosis, late apoptosis, and necrosis marked with Annexin V-FITC and PI, was performed by flow cytometry after exposure to **Cu1a** and **Cu1b** complexes for 48 h at their IC_50_ concentrations. Three controls (DMSO 0.1%, Cis, and Dox) were also included for comparison. The presented results are expressed as mean ± SEM obtained from at least two independent biological assays. To assess the statistical significance of these results compared to the control group treated with DMSO, the one-way ANOVA method was used (* *p* ≤ 0.05; ** *p* ≤ 0.005; *** *p* ≤ 0.0005; **** *p* ≤ 0.0001).

The results presented in Figure 6 and Table 8 show that in the negative control of 0.1% (v/v) DMSO, the cell viability rate was 87.4%, in line with expectations.^67,69^ Additionally, there was a proportion of 9.5% of cells in early apoptosis, 1.6% in late apoptosis, and 1.5% in necrosis. Regarding cells treated with Dox, a lower percentage of viable cells was observed, about 59.8%, with 26.6% in early apoptosis, 13.9% in late apoptosis, and the highest percentage of necrosis at 9.8%, consistent with literature findings.^69^ Cisplatin (Cis) was used as a positive control, exhibiting the highest percentage of cells in early apoptosis at 40.1 and 25.8% in late apoptosis. These results are consistent with those who demonstrated the induction of apoptosis by both Cis and Dox.^70^ The concentrations of Cis and Dox used in the study were determined based on previous assays conducted in the laboratory^62,67^ (Table 3, Figure S10).

**Table 8:** Percentage of Viable, Early Apoptotic, Late Apoptotic, and Necrotic Cells in HCT116-DoxR Cells Exposed to DMSO 0.1% (Vehicle Control), Dox, and Cis as Positive Controls, and Exposure of 48 h to the IC_50_ Concentrations of Complexes **Cu1a** and **Cu1b**

controls	viable cells	early apoptosis	late apoptosis	necrosis
marked cells	73.5% ± 7.9	22.7% ± 8.8	3.3% ± 1.1	0.5% ± 0.4
DMSO	87.4% ± 6,3	9.5% ± 6.4	1.6% ± 1.5	1.5% ± 1.1
cisplatin	33.4% ± 5.5	40.1% ± 11.8	25.8% ± 7.7	0.9% ± 1.1
doxorubicin	59.8% ± 3.8	16.6% ± 5.5	13.9% ± 1.9	9.8% ± 4.0
**Cu(II) complexes**				
Cu1a	23.1% ± 4.5	55.2% ± 4.9	21.7% ± 8.2	0.0% ± 0.1
Cu1b	59.3% ± 7.8	33.1% ± 5.3	6.9% ± 3.4	0.7% ± 0.6

The results shown in Figure 6 and Table 8 also indicate that cells exposed to complexes **Cu1a** and **Cu1b**, exhibit a statistically significant increase in the percentage of cells undergoing apoptotic processes when exposed for 48 h to their IC_50_ concentrations. In the case of cells exposed to complex **Cu1a**, a cell survival rate of 23.1% was observed, with most cells in early apoptosis (55.2%), 21.7% in late apoptosis, and no necrosis. On the other hand, complex **Cu1b** shows a percentage of 33.1% of cells in early apoptosis and 6.9% in late apoptosis. In agreement with cytotoxicity data (Table 3) and internalization (Tables 6 and 7), complex **Cu1a** induces a higher percentage of cells in apoptosis when compared to complex **Cu1b**, which is even higher than the % of apoptotic cells in cisplatin (Figure 6 and Table 8).

Cells treated with these metallic complexes are predominantly in early apoptosis (Table 8). The ability of Cu(II) complexes to induce apoptosis had been described previously.^24,69^ The percentage of cells in necrosis was less than 1% under all conditions studied except for cells exposed to Dox (9.8%) and DMSO (1.5%), suggesting that the complexes do not induce necrosis significantly (Table 8). Given that the necrosis mechanism involves the recruitment of inflammatory molecules and is associated with tumor progression, the **Cu1a** and **Cu1b** complexes seem to be highly promising.

Under stress conditions, the proapoptotic protein BAX undergoes a conformational change and acquires the ability to translocate to the mitochondrial outer membrane. This results in the disruption of the membrane potential and, consequently, pore formation.^68^ For this reason, it becomes relevant to investigate whether the levels of pro- and antiapoptotic proteins are altered and if the complexes induce changes in the mitochondrial membrane potential (ΔΨ_m_).^70^

### Evaluation of Mitochondrial Membrane Potential (ΔΨ_m_)

**Figure 7:**
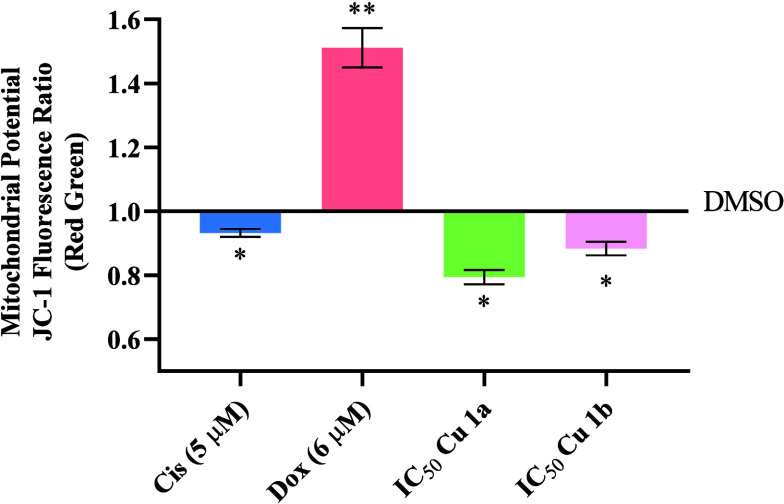
Evaluation of the mitochondrial membrane potential in HCT116-DoxR tumor cells after 48 h of exposure to the IC_50_ concentration of complexes **Cu1a** and **Cu1b** through labeling with JC-1probe. The cells were exposed to the 0.1% DMSO vehicle control and positive controls of Cis (5 μM) and Dox (6 μM). The presented data are normalized to DMSO and shown as the mean ± SEM of two independent assays using the Student *t* test method (* *p* ≤ 0.05, ** *p* ≤ 0.005).

In the early stages of apoptosis, the rupture of the mitochondrial outer membrane and the dissipation of the mitochondrial membrane potential (Ψ_M_) are distinguishing features.^71^ Changes in membrane potential can be measured and represent an essential parameter, indicating the functional state of the mitochondria. For this analysis, the lipophilic cationic probe 5,5′,6,6′-tetrachloro-1,1′,3,3′-tetraethylbenzimidazolylcarbocyanine iodide (JC-1) was used, allowing the evaluation of depolarization or hyperpolarization of the mitochondrial membrane.^72^ In healthy cells, JC-1 accumulates inside the electronegative mitochondria as aggregates (red fluorescence) when the membrane potential is positively charged (hyperpolarized).^71^ However, when depolarization of the mitochondrial membrane potential occurs, the JC-1 probe will accumulate outside of the mitochondria in its monomeric form (green fluorescence), therefore causing a decrease in the ratio of JC-1 aggregate/monomer fluorescence intensity.^61,71^ HCT116-DoxR cells were exposed to concentrations corresponding to the IC_50_ concentrations of complexes **Cu1a** and **Cu1b** for 48 h. Subsequently, the cells were stained with the JC-1 probe and subjected to flow cytometry analysis to confirm potential alterations in the mitochondrial membrane potential induced by the complexes. 0.1% (v/v) DMSO was used as vehicle control and Dox (6 μM) and Cis (5 μM) as positive controls. The analysis reveals that the red/green fluorescence ratio is less than 1 for the two complexes as well as for the positive control cisplatin (Figure 7). As observed, complex **Cu1a** induced the greatest depolarization with a ratio of approximately 0.79, followed by complex **Cu1b** with a ratio of 0.88 (Figure 7). This indicates that the JC-1 probe is predominantly in monomeric form, indicating a loss of membrane potential and statistically significant depolarization. It is important to mention that Dox did not induce membrane depolarization since the cell line used is resistant to this drug, and the concentration used maintains resistance but is not sufficient for a change in mitochondrial potential, as observed previously by Choroba et al.^64^ These results (Figure 7) are in line with the results obtained for the subcellular localization of the complexes in the mitochondrial fraction (Table 7) (with a higher accumulation for **Cu1a**), that may induce a change in ΔΨ_m_ of the mitochondria membrane, triggering the intrinsic apoptotic pathway (also higher for **Cu1a** compared to **Cu1b**) (Figures 6 and 7).

### Quantification of BAX and BCL-2 Protein Expression

The dysregulation of the mitochondrial-intrinsic apoptotic pathway is one of the most crucial events in the context of carcinogenesis. The BCL-2 protein family plays a crucial role in regulating this intrinsic pathway, comprising antiapoptotic proteins (e.g., BCL-2 and BCL-xl) that prevent membrane disruption and pro-apoptotic proteins (e.g., Bax, Bak) that promote apoptosis.^73,74^ The balance between the BAX/BCL-2 ratio determines the susceptibility of cells to choose the intrinsic apoptotic pathway (ratio >1) (increase in BAX protein expression) or favor cell survival (ratio <1) (increased expression of BCL-2 compared to BAX).^74^ Low levels of this ratio can result in the resistance of cancer cells to apoptosis. Therefore, there is a clear relationship between the Bax/BCL-2 ratio and the impact on tumor progression and aggressiveness.^74^

The expression of both proteins was quantified by Western Blot after incubation of HCT116-DoxR cells for 48 h with IC_50_ concentrations of complexes **Cu1a** and **Cu1b** (Figure S18). 0.1% DMSO was used as a negative control and β-actin protein was used for the normalization of the total protein quantity.

**Figure 8:**
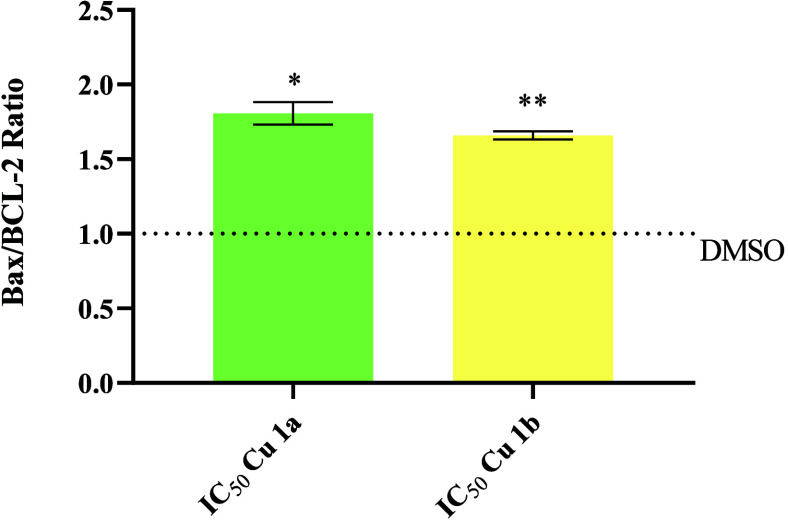
BAX/BCL-2 ratio in HCT116-DoxR cells after 48 h of incubation with the 0.1% DMSO vehicle control or the IC_50_ concentrations of complexes **Cu1a** and **Cu1b**. The data were normalized to the control and subsequently normalized to the results of β-actin. The provided results are represented as the mean ± SEM obtained from at least two independent biological assays. To assess the statistical significance of these results compared to the DMSO-treated control group, the Student’s *t* test was utilized (* *p* ≤ 0.05; ** *p* ≤ 0.005).

Upon observing Figure 8, it is evident that, for all complexes, the BAX/BCL-2 ratio was greater than 1, indicating significantly higher levels of the pro-apoptotic protein BAX compared to BCL-2, when compared to the 0.1% DMSO control. Complex **Cu1a** exhibited the highest expression levels with a ratio 1.80× higher than that of the control, followed by complex **Cu1b** with a ratio 1.65× higher than that of the control (Figure 8).

Taken together, these results (Figure 8) are in line with previous data (Figures 6 and 7, and Tables 6 and 7), namely, **Cu1a** and **Cu1b** cellular internalization (Table 6), accumulation in the mitochondria (Table 7), and the depolarization of the mitochondrial membrane potential (Figure 7), which triggers BAX induction of the intrinsic or mitochondrial apoptotic process (Figure 6).

### Evaluation of Autophagy

Furthermore, previous studies have shown that complexes containing terpyridine derivatives have the ability to trigger both apoptosis and autophagy cell death mechanisms in cancer cells simultaneously.^38^ This convergence of death pathways may arise from an adaptive response of the heterogeneous tumor cell population.^38^ In this context, understanding the mechanism by which cells lose viability in the presence of the two Cu(II) complexes with the simultaneous induction of apoptosis and autophagy becomes crucial. Autophagy is a highly conserved catabolic process crucial for maintaining cellular homeostasis but is also associated with type II programmed cell death.^75^ It involves intracellular degradation through autophagolysosomes responsible for breaking down luminal content and enabling recycling of cellular components.^76,77^ The assessment of the complexes’ ability to induce autophagy was conducted using flow cytometry, employing the CYTO-ID green detection reagent. This probe emits green fluorescence, marking the autophagic vacuoles within the cell. Higher fluorescence indicates a greater proportion of autophagolysosomes.^78^ Three positive controls were used: Dox, Cis, and rapamycin (1500 nM), whose concentration was previously optimized for the HCT116-DoxR cell line in the laboratory.^64^ Rapamycin is known to induce autophagy through its mechanistic target, mTOR.

**Figure 9:**
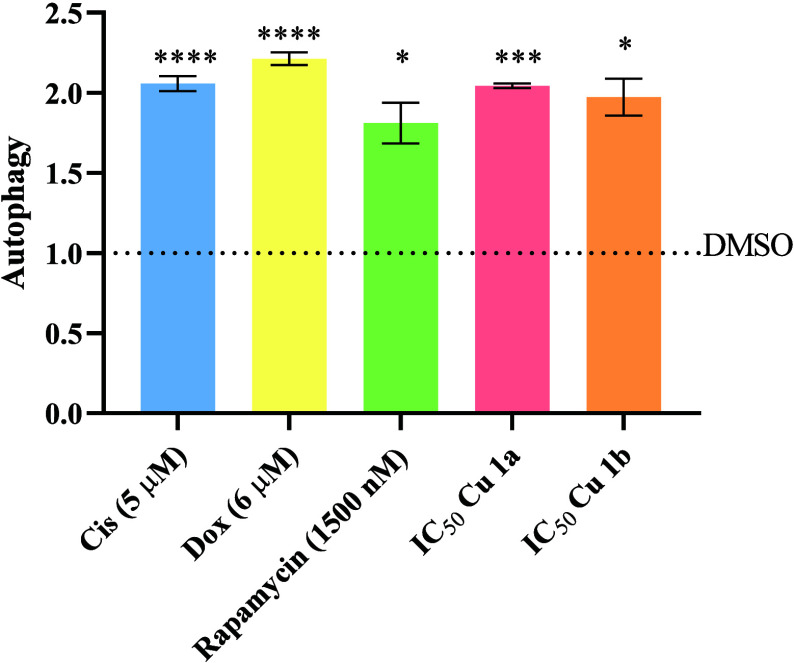
Quantification of HCT116-DoxR tumor cells undergoing autophagy by flow cytometry after exposure for 48 h to the IC_50_ concentrations of complexes **Cu1a** and **Cu1b** or one of the four controls (0.1% DMSO, Cis, Dox, and rapamycin). The provided results are shown as the mean ± SEM obtained from at least two independent biological assays. To assess the statistical significance of these results compared to the DMSO-treated control group, the Student’s *t* test method was used (**** *p* ≤ 0.0001; *** *p* ≤ 0.0005; ** *p* ≤ 0.005; * *p* ≤ 0.05).

As shown in Figure 9, an increase in green fluorescence intensity is observed in cells exposed to Cis, Dox, and rapamycin controls, as well as in the presence of the two Cu(II) complexes, compared to the 0.1% DMSO control. Indeed, complexes **Cu1a** and **Cu1b** revealed an approximately 2×-fold significant increase in autophagic vesicles. Dox, among the positive controls, exhibited the highest induction of autophagy, being 2.2 times higher compared to the negative control (Figure 9). In summary, the results reinforce the complexes’ ability to induce the autophagic process, showing levels comparable to the positive controls of doxorubicin and cisplatin, albeit slightly higher than rapamycin, which is also in line with the higher accumulation of **Cu1a** and **Cu1b** complexes in the cytosolic fraction which may also contribute to the trigger of this process (Table 7 and Figure 9).

### Evaluation of Reactive Oxygen Species (ROS) Production

The literature widely describes the relationship between the induction of these types of programmed cell death and the production of reactive oxygen species (ROS).^79−81^ ROS can arise from endogenous or exogenous sources and play a role in carcinogenesis and tumor development, causing DNA damage.^81,82^ The accumulation of high ROS levels results in oxidative stress in cancer cells, a condition that can be exploited by surpassing the intrinsic antioxidant capacity of tumor cells as a targeted therapeutic approach.^75^ It is known that Cu(II) complexes induce ROS production.^38^ In this regard, the production of reactive oxygen species was quantified in HCT116-DoxR cells using the 2′,7′-dichlorodihydrofluorescein diacetate (H_2_DCF-DA) probe, which is rapidly oxidized in the presence of ROS, generating the compound 2′,7′-dichlorodihydrofluorescein (DCF), whose fluorescence can be detected by flow cytometry.^71^ The fluorescence intensity is directly proportional to the amount of intracellular ROS. For this, HCT116-DoxR cells were exposed for 48 h to the IC_50_ concentrations of complexes **Cu1a** and **Cu1b**. Cisplatin and Dox were used as positive controls, and 0.1% DMSO served as the vehicle control. Tert-butyl hydroperoxide (TBHP) solution at 42 μM, corresponding to the previously determined IC_50_for the HCT116-DoxR cell line, was used as a positive control for ROS production.^64^ TBHP is a widely used organic peroxide in oxidative stress studies as it activates caspase-mediated apoptosis and ROS production.^83^

**Figure 10:**
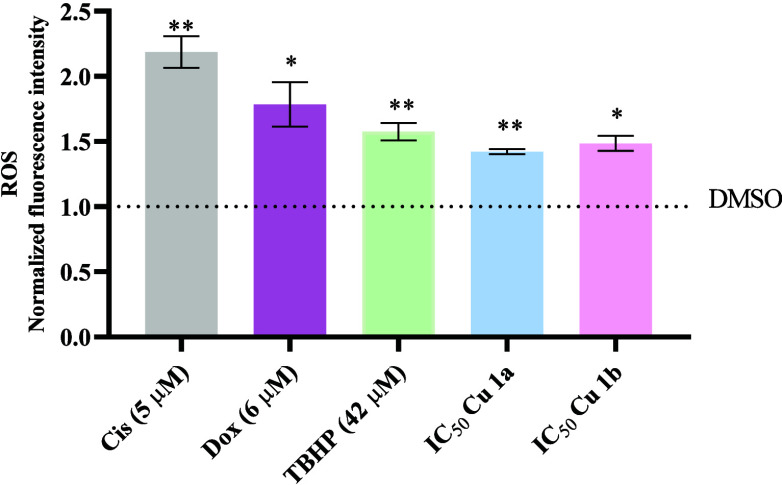
Quantification of reactive oxygen species (ROS) induction in viable HCT116-DoxR tumor cells after 48 h of exposure to the **Cu1a** and **Cu1b** complexes at their respective IC_50_ concentrations. Four controls (0.1% DMSO, Cis, Dox, and TBHP) are also represented. The graph is normalized to DMSO. The provided results are shown as mean ± SEM obtained from at least two independent biological assays. To assess the statistical significance of these results compared to the DMSO-treated control group, the Student *t* test method was used * *p* ≤ 0.05; ** *p* ≤ 0.005).

As shown in Figure 10, complexes **Cu1a** and **Cu1b** can induce ROS production with values 1.42× and 1.48×, respectively, higher than the DMSO control. However, ROS production by the complexes is not as high as that obtained for the positive controls of Dox and Cisplatin. Cells exposed to Cisplatin and Dox controls exhibited ROS production values of 2.2× and 1.8×, respectively, higher than the DMSO control, even surpassing the TBHP positive control (Figure 10).

This increase in the intracellular ROS by both Cu(II) complexes is in line with their accumulation in the cytosol and particularly mitochondria, where most intracellular ROS is produced (Table 7). Previous studies have reported a correlation between increased ROS and cell death, involving Au(II), Cu(II), and Pt(II) complexes with terpyridine ligands in HCT116 cells.^38^ The results in Figure 10 indicate that when HCT116-DoxR cells are exposed to the **Cu1a** and **Cu1b** metal complexes, there is a statistically significant increase in intracellular ROS production compared to DMSO control (Figure 10) that together with their accumulation in the cytosol and mitochondria (Table 7) may trigger BAX overexpression, mitochondria depolarization and the trigger of intrinsic apoptosis and autophagy (Figures 6**–**9).

### Cell Cycle Progression Analysis

It is important to note that exposure to increased levels of ROS can trigger genotoxic stress, an effect often associated with chemotherapy and that may promote cell cycle arrest.^84^ The cell cycle comprises a sequential set of cellular events such as checkpoints that regulate transitions between different phases when cells are under stress conditions. Indeed, if DNA damage occurs, interphase checkpoints can trigger cell cycle arrest, repair, and induction of apoptosis or senescence.^85^ Indeed, data in Table 7 show some degree of accumulation of both complexes in the nucleus, which may expose the genomic DNA to their action. One way to confirm whether the Cu(II) complexes, besides being cytotoxic to HCT116-DoxR cells, may also be cytostatic, is the analysis of the cell cycle progression when HCT116-DoxR cells are incubated or not in the presence of the **Cu1a** and **Cu1b** complexes. In this assay, the cytostatic effect of the complexes **Cu1a** and **Cu1b** and their influence on cell cycle progression were investigated using PI. This DNA intercalating fluorophore allows the analysis of DNA content in various phases of the cell cycle (G0/G1, S, and G2/M) (the fluorescence intensity emitted by PI doubles with the duplication of DNA quantity in the G1 and G2 phases),^86^ and its detection can be performed by flow cytometry. HCT116-DoxR cells were exposed to IC_50_ concentrations of complexes **Cu1a** and **Cu1b** for 9, 12, 18, and 24 h of incubation. A 0.1% (v/v) DMSO solution was used as a vehicle control, and Dox and Cisplatin were used as positive controls. To ensure that all cells were in the same phase of the cell cycle (S phase) when exposed to the Cu(II) complexes and controls, a double thymidine block was applied.^83^

**Figure 11:**
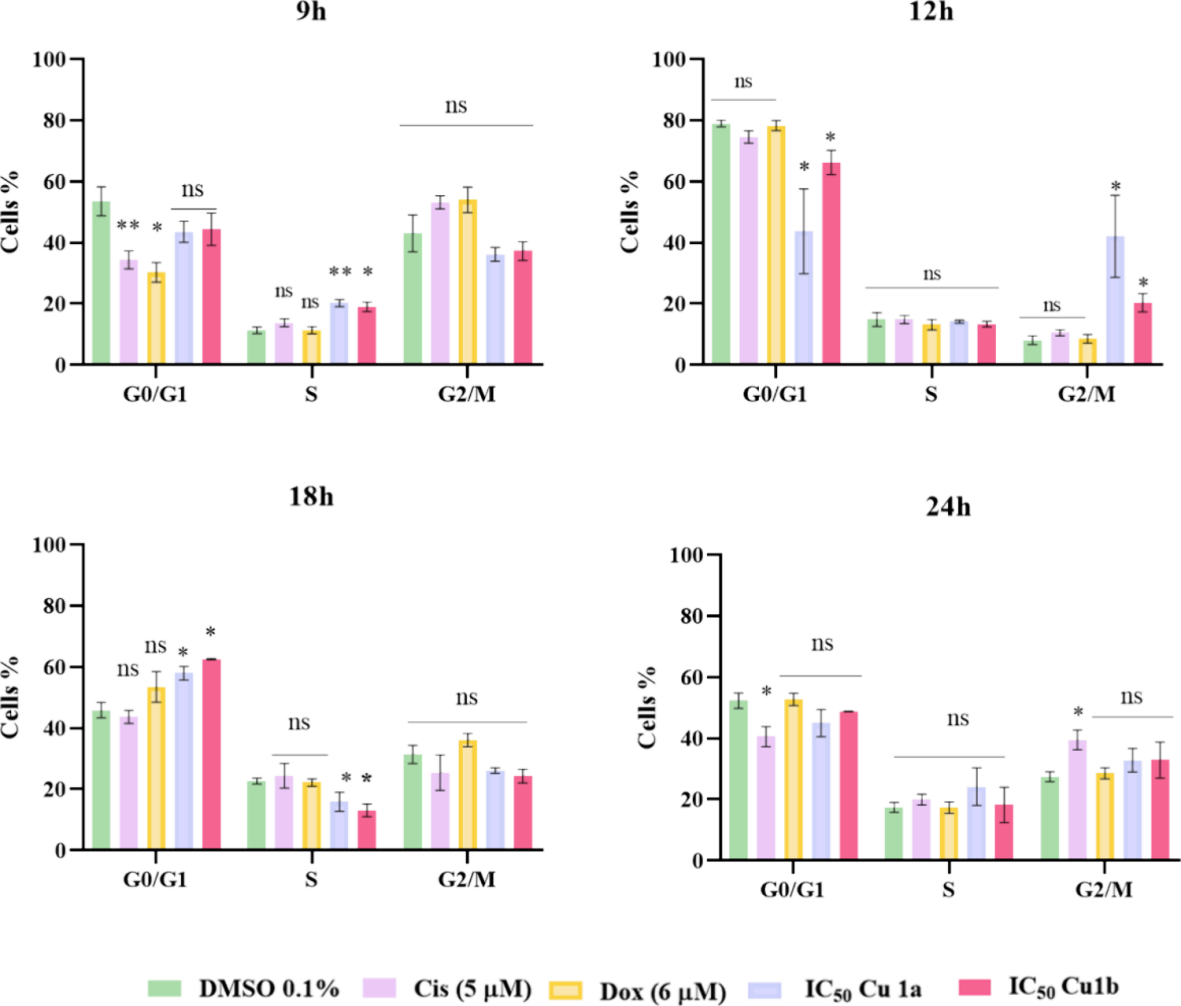
Cell cycle progression in viable HCT116-DoxR tumor cells after 48 h of exposure to the IC_50_ concentrations of complexes **Cu1a** and **Cu1b**. Three controls (0.1% DMSO, Cis, and Dox) are also represented. The provided results are shown as mean ± SEM obtained from at least two independent biological assays. To assess the statistical significance of these results compared to the DMSO-treated control group, the Student *t* test method was used (* *p* ≤ 0.05; ** *p* ≤ 0.005; *** *p* ≤ 0.0005).

The results illustrated in Figure 11 show that the Cisplatin positive control has a cytostatic effect in G2/M after incubation for 24 h of incubation. On the other hand, due to the resistance of these cells to Dox, the concentration used did not affect the cell cycle progression or cause an arrest. On the other hand, the Cu(II) complexes, **Cu1a** and **Cu1b**, exhibited a delay in the cell cycle at the S phase after 9 h, in the G2/M after 12 h of incubation, and a delay in the cell cycle at G0/G1 at 18 h (Figure 11). These different cell cycle delays might represent the heterogeneity of the tumor population and the induction of different types of cell death mechanisms(apoptosis and/or autophagy) by each individual cell considering the intensity of stress that they are subjected to.

According to the literature, other copper complexes have demonstrated the ability to block the cell cycle in the G0/G1 phase.^71^

### Evaluation of Cellular Senescence

Senescence is a process involving a stable, nonreversible cell cycle arrest intended to regulate cellular fate. Senescence is typically activated by intrinsic and extrinsic stimuli that induce DNA damage, oxidative stress, or nutrient deprivation. The cellular response to stimuli or damage usually varies according to the cell type, intensity, and nature of the stimulus, and can manifest as damage repair, cell death, or senescence.^80^

The percentage of senescent cells was assessed using the Senescence Assay Kit (Abcam), enabling the quantification of beta-galactosidase activity through flow cytometry. HCT116-DoxR cells were exposed to the IC_50_ concentrations of complexes **Cu1a** and **Cu1b** for 48 h. A 0.1% (v/v) DMSO solution served as vehicle control, and Dox and Cis were used as positive controls.

**Figure 12:**
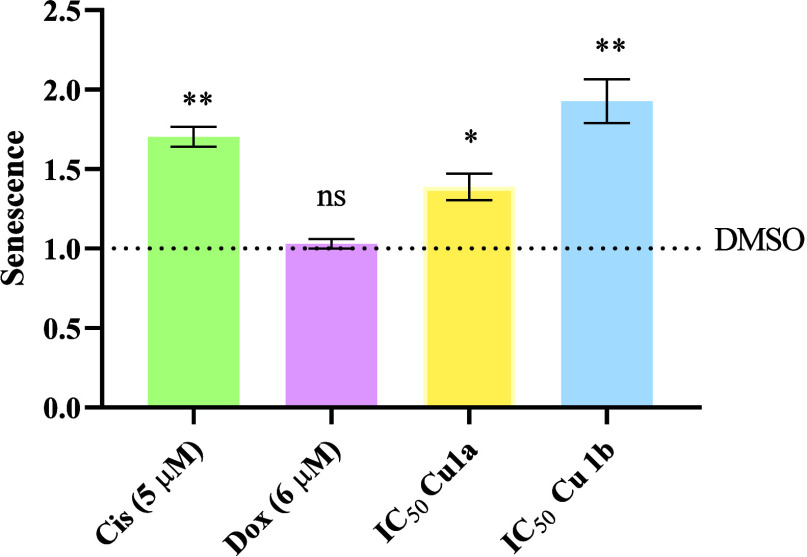
Quantification of senescent HCT116-DoxR cells after exposure for 48 h to the IC_50_ concentrations of complexes **Cu1a** and **Cu1b**. 0.1% (v/v) DMSO was used as a vehicle control, and Dox (6 μM) and Cis (5 μM) were used as positive controls. The provided results are shown as mean ± SEM obtained from at least two independent biological assays. To assess the statistical significance of these results compared to the DMSO-treated control group, the Student’s *t* test method was used (* *p* ≤ 0.05, ** *p* ≤ 0.005).

The results portrayal in Figure 12 indicate an increase in the percentage of senescent cells after incubation with **Cu1a** and **Cu1b** complexes. Remarkably, complex **Cu1b** exhibited the highest quantity of cells in senescence, consistent with the cell cycle results (Figure 11). This complex demonstrates the most significant cell cycle delay, with a halt in the cycle between 12–18 h, resulting in an increase in the formation of senescent cells.

In conclusion, the capability of complexes to enter the nucleus (Table 7), together with their ability to induce an increase in the intracellular ROS levels (Figure 10), may interfere with the cell cycle progression and induce also premature cellular senescence (Figures 11 and 12).

### DNA Interaction Studies

Based on the previous data, such as the ability of Cu(II) complexes to enter the nucleus, generate ROS, and the observed delay in the cell cycle progression, it is important to understand if both, **Cu1a** and **Cu1b,** complexes can interact with DNA. In this regard, three different analytical techniques were used to study the interaction of both complexes with DNA: (i) ethidium bromide displacement assay^87,88^ and (ii) UV–vis absorption titration,^89,90^ both using calf-thymus DNA (ctDNA), and (iii) interaction with plasmid DNA (pDNA).

#### Ethidium Bromide Displacement Assay

**Figure 13:**
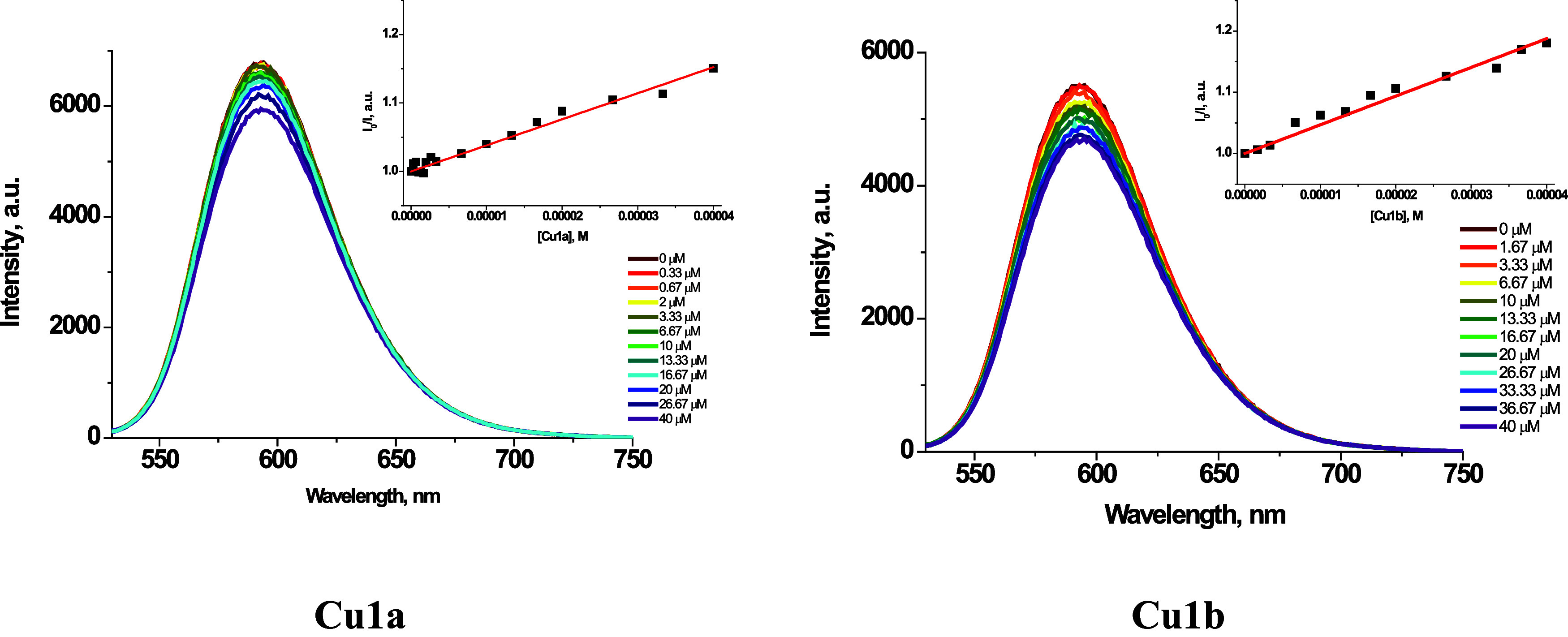
Emission spectra (λ_ex_ = nm) of EB–ctDNA in PBS buffer with the increasing concentration of **Cu1a** and **Cu1b**. The insets show the Stern–Volmer plots for the corresponding complexes.

First, fluorescence spectroscopy was used to investigate the ability of complexes **Cu1a** and **Cu1b** to displace ethidium bromide (EB) from the ctDNA-EB adduct. Upon photoexcitation in the range from ∼480 to ∼520 nm, the adduct ctDNA-EB shows a strong emission at 596 nm, with an intensity 10 times higher compared to that observed for free EB.^89^ Compounds that intercalate between DNA base pairs equally or more strongly than EB replace it from the adduct ctDNA-EB, which results in a noticeable decrease or quenching of the fluorescence emission. The emission spectra of ctDNA-EB in PBS buffer in the presence of changing concentrations of complexes **Cu1a** and **Cu1b** are presented in Figure 13. For both **Cu1a** and **Cu1b**, the decrease in the fluorescence intensity of ctDNA-EB with increasing complex concentrations fulfills the linear Stern–Volmer equation (eq 3)^90^:
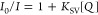
3

Calculated *K*_sv_ constants are equal to 3.8 × 10^3^ and 4.7 × 10^3^ M^–1^ for **Cu1a** and **Cu1b**, respectively (see insets in Figures 13 and S19–S20, ESI).

The apparent binding constants (*K*_app_) are estimated using eq 4:
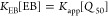
4(where *K*_EB_ = 1.0 × 10^7^ M^–1^, [EB] is the EB concentration (50 μM), [*Q*_50_] is the complex concentration at 50% reduction of EB fluorescence intensity, estimated based on the linear Stern–Volmer plot for *I*^0^/*I* = 2). *K*_app_ is equal to 1.75 × 10^5^ M^–1^ for **Cu1a** and 2.35 × 10^6^ M^–1^ for **Cu1b**. In agreement with the more planar ligand geometry of 2-naphtyl-terpy than 1-naphtyl-terpy, complex **Cu1b** shows a higher tendency to release EB from ctDNA-EB adduct. Nevertheless, both Cu(II) complexes show rather moderate ability to replace EB from the adduct with ctDNA.^21^

#### UV–Vis Absorption Titration

**Figure 14:**
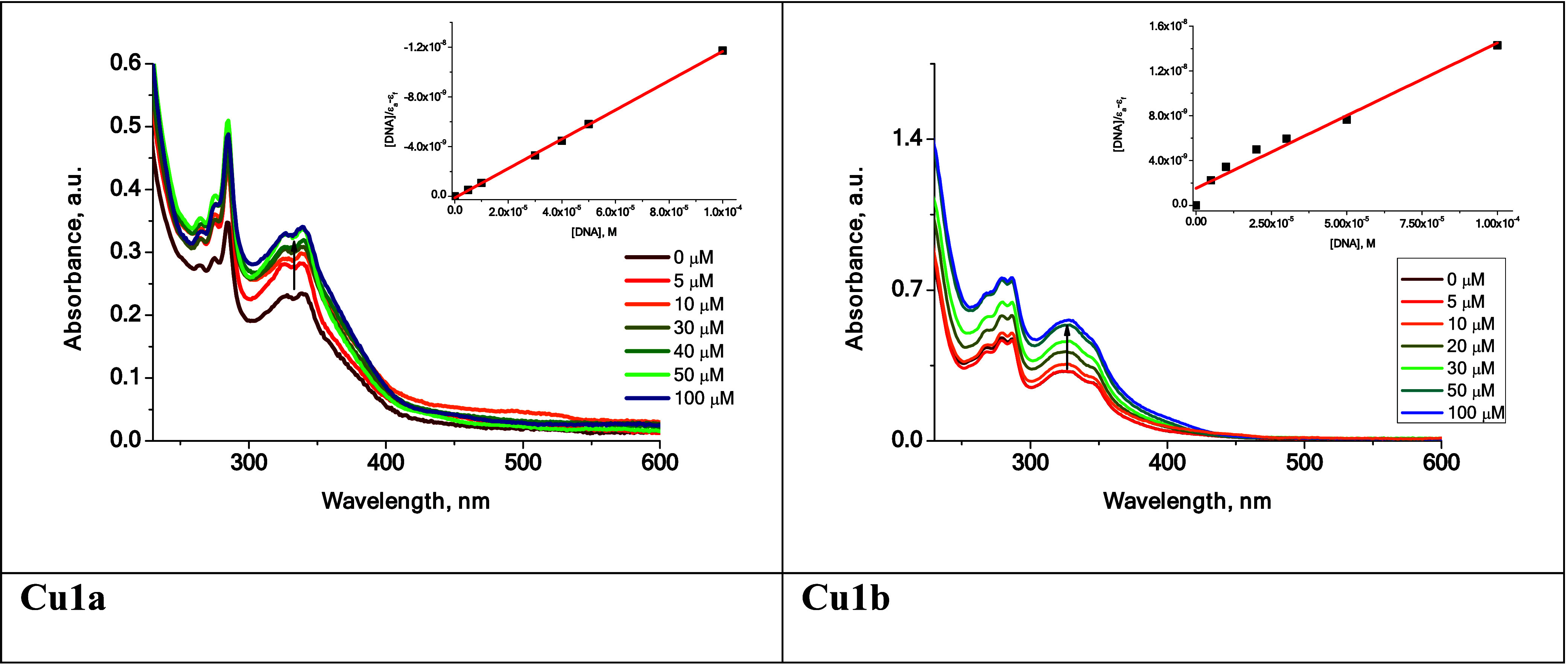
UV–vis absorption spectra of **Cu1a** and **Cu1b** solutions in PBS (*c* = 25 μM) in the absence or presence of ctDNA (*c*: 0–100 μM). The insets show the Wolfe–Shimmer plots for the corresponding complexes.

To gain further insight into the nature of interactions of complexes **Cu1a** and **Cu1b** with DNA, the solutions of Cu(II) complexes in PBS buffer were titrated with increasing amounts of ctDNA (0–100 μM), and changes in the lowest-energy absorption band of drugs were monitored by UV–vis spectroscopy. For both Cu(II) complexes, the addition of DNA results in a gradual increase in the absorption intensity of the band at ∼350 nm, while the band position remains the same (Figure 14). The hyperchromic effect is indicative of nonintercalative interactions with DNA, most likely via groove binding.^91,92^ Based on the variations in the absorbance of **Cu1a** and **Cu1b** upon the DNA addition, the intrinsic binding constants *K*_b_ were calculated via Wolfe–Shimmer equation (eq 5):
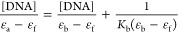
5

Plotting [DNA]/ε_a_– ε_f_ versus [DNA], where [DNA] represents the concentration of DNA, and ε_a_, ε_f_ and ε_b_ are the apparent extinction coefficient (*A*_obs_/[M]), extinction coefficient for free metal complex (M) and extinction coefficient for the free metal complex (M) in the fully bound form, respectively (see inset, Figures 14 and S21–S22, ESI), gives *K*_b_ values, 1.10 × 10^6^ M^–1^ for **Cu1a** and 0.85 × 10^6^ M^–1^ for **Cu1b**. These values fall in the range reported previously for related Cu(II) complex with 4′-((naphthalen-2-yl)methoxy)-2,2′:6′,2″-terpyridine^21^ that was proposed as an “outside binder”.

Our in vitro data show that both Cu(II) complexes may interact with ctDNA by a nonintercalative mode, most likely via groove binding (“outside binders”) which also has the ability to replace EB from the adduct with ctDNA (Figures 13 and 14).

#### Plasmid DNA (pDNA) Interaction and Cleavage

**Figure 15:**
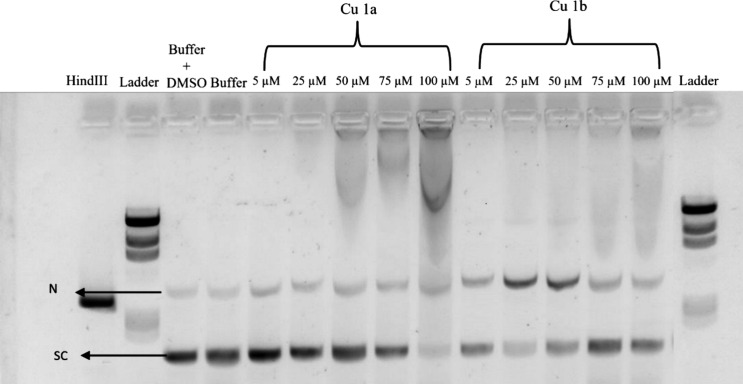
Gel electrophoresis in 0.8% agarose gel with TAE 1× buffer containing 0.0015% Gel Red was performed to assess the integrity of all samples exposed to the complexes. The electrophoresis conditions included a constant voltage of 90 V for 90 min. All samples containing the complexes were incubated in an oven at 37 °C for 24 h. Each sample contained 100 ng of cell-derived pDNA. The figure legends are as follows: Ladder: Molecular weight marker lambda *Hin*dIII; *Hin*dIII: pUC18 sample incubated with *Hin*dIII for 2 h Buffer: pUC18 sample exposed to 5 mM Tris-HCl and 50 mM NaCl pH 7.2 buffer for 24 h; Buffer+ DMSO: pUC18 sample exposed to 5 mM Tris-HCl and 50 mM NaCl pH 7.2 buffer with 1% (v/v) DMSO for 24 h; 5 μM: pUC18 sample exposed to 5 μM of the indicated complexes **Cu1a** and **Cu1b** complexes; 25 μM: pUC18 sample exposed to 25 μM of the indicated complexes **Cu1a** and **Cu1b**; 50 μM: pUC18 sample exposed to 50 μM of the indicated complexes **Cu1a** and **Cu1b**; 75 μM: pUC18 sample exposed to 75 μM of the indicated complexes **Cu1a** and **Cu1b**; 100 μM: pUC18 sample exposed to 100 μM of the indicated complexes **Cu1a** and **Cu1b**. Black arrows indicate the isoforms of the pUC18 plasmid: N, nicked circular isoform; SC, supercoiled isoforms.

In order to further understand the interaction of Cu(II) complexes with DNA, and the possible genotoxic damage due to ROS generated by the Cu(II) complexes, incubation of pUC18 plasmid DNA (pDNA) with **Cu1a** or **Cu1b** was performed. The procedure involved exposing 100 ng of pDNA (pUC18) to increasing concentrations of complexes **Cu1a** and **Cu1b** (5, 25, 50, 75, and 100 μM), which were incubated at 37 °C for 24 h. Controls were also performed with pUC18 in 5 mM Tris-HCl and 50 mM NaCl pH = 7.2 buffer in 1% (v/v) DMSO, in addition to control with pUC18 preincubated with the *Hin*dIII restriction enzyme for 2 h, as *Hin*dIII is a restriction endonuclease that cleaves the phosphodiester bonds of the DNA structure,^54^ and can be used as a positive control for the linearized plasmid isoform (L) (Figure 15). The pUC18 plasmid is typically found in a supercoiled conformation (SE), which undergoes greater migration due to its compact structure, and in a smaller fraction as a circular isoform (nicked, N), due to the cleavage of one of the strands, and identified as the uppermost band on the gel (Figure 15). Alternatively, if both strands are cleaved, they exhibit a linear conformation (L) (Figure 15).

In the DMSO and buffer controls, only two isoforms, circular and supercoiled, are observed, while the positive control of the linear form with the *Hin*dIII enzyme exhibits a single band corresponding to the linear isoform (L) as expected (Figure 15).

When pDNA samples were exposed to increasing concentrations of the complex **Cu1a**, an increase in the circular form (N) was observed compared to negative control with the simultaneous decrease of the supercoiled isoform (Figure 15). For concentrations higher than 50 μM, an apparent retention of the pDNA in the well is observed, which is in line with previous results (Figures 13 and 14) and the nonintercalative interactions with pDNA, most likely via groove binding and a modification in its migratory profile (Figures 15 and Supplementary Figure S23). At a concentration of 100 μM, an almost total disappearance of the supercoiled isoform is observed, primarily attributed to the increased retention in the well.

Exposure to different concentrations of complex **Cu1b** resulted in a decrease in supercoiled forms and an increase in the intensity of the circular isoform at concentrations of 25 and 50 μM. However, for higher concentrations (75 and 100 μM), increased retention in the wells also occurred which affected the pDNA cleavage (Figures 15 and Supplementary Figure S23).

Considering all of the results obtained so far, particularly the ability of the complexes to induce ROS, and after determining the optimal concentration at which the complexes cleave pDNA (50 μM), the underlying mechanism of cleavage was investigated. The sodium azide (NaN_3_) scavenger, capable of quenching singlet oxygen (^−1^O_2_), was used for this purpose.^93^ Additionally, hydrogen peroxide (H_2_O_2_) was employed as a well-known positive control for DNA cleavage through oxidative mechanisms in the presence of ROS.^71^

**Figure 16:**
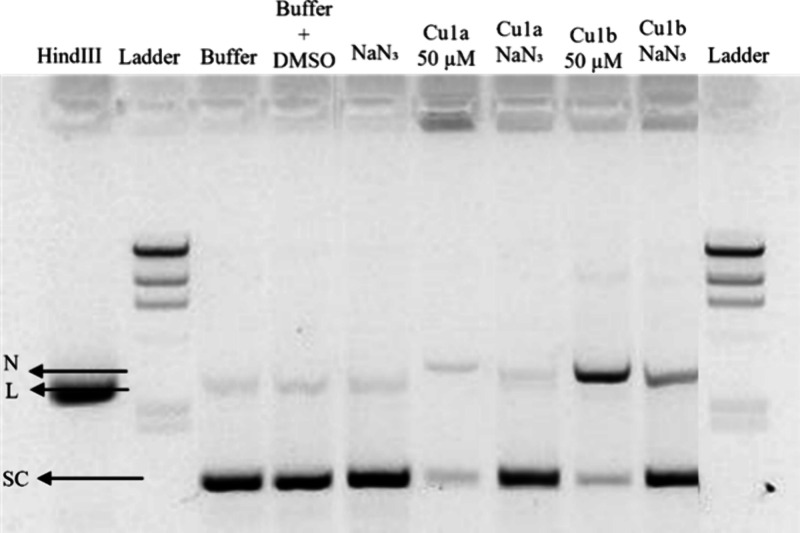
Gel electrophoresis was performed using a 0.8% agarose gel in TAE 1× buffer with 0.0015% Gel Red, applying a current of 70 V for 90 min. This aimed to determine the pDNA cleavage mechanisms of complexes **Cu1a** and **Cu1b** after incubation at 37 °C for 24 h. The legend for the gel is as follows: Ladder: Molecular weight marker lambda *Hin*dIII; *Hin*dIII: pUC18 sample exposed to *Hin*dIII activity for 2 h; Buffer: pUC18 sample exposed to 5 mM Tris-HCl and 50 mM NaCl pH = 7.2 buffer for 24 h; Buffer + DMSO: pUC18 sample exposed to 5 mM Tris-HCl and 50 mM NaCl pH = 7.2 buffer with 1% (v/v) DMSO for 24 h; NaN_3_: pUC18 sample exposed to 50 μM NaN_3_; **Cu1a** 50 μM: pUC18 sample exposed to 50 μM of complex **Cu1a**; **Cu1b** 50 μM: pUC18 sample exposed to 50 μM of complex **Cu1b**; **Cu1a** NaN_3_: pUC18 sample exposed to 50 μM of complex **Cu1a** and 50 μM NaN_3_; **Cu1b** NaN_3_: pUC18 sample exposed to 50 μM of complex **Cu1b** and 50 μM NaN_3_. Black arrows represent the identified isoforms of pUC18 on the gel: N, nicked isoform; L, linear isoform of pUC18; SE, supercoiled isoform.

In Figure 16 and similarly to what was observed in Figure 15, the three bands corresponding to the top DNA isoforms are shown. For the DMSO and Buffer controls, only two bands are observed, indicating the presence of circular and supercoiled forms. In contrast, the *Hin*dIII control shows only the band corresponding to linear DNA, owing to its specific cleavage activity.

The NaN_3_ scavenger, when used alone, showed no capacity to cleave pDNA, exhibiting a profile similar to that of the negative controls. For complex **Cu1a**, low-intensity bands corresponding to the circular and supercoiled isoforms are observed, primarily due to significant retention (Figure 16). However, when this complex is combined with the scavenger agent, an increase in the supercoiled isoform and a reduction in the nicked isoform are observed, similar to controls. For complex **Cu1b,** and as previously observed for 50 μM, a high cleavage of pDNA occurs, evidenced by a band corresponding to the circular isoform on the gel. When complex **Cu1b** was incubated with NaN_3_, an increase in the supercoiled isoform and a decrease in the nicked isoform were observed (decreased pDNA cleavage). This demonstrates that NaN_3_, by sequestering oxygen singlets, is reducing the cleavage capability of the complexes (Figures 16 and S23).

In conclusion, the analysis of the results allows us to infer that the mechanism of cleavage of pDNA by the two complexes is dependent on singlet oxygen radicals (^−1^O_2_). This conclusion aligns with the findings from the ROS production assay (Figure 10), where it was demonstrated that these complexes have a propensity to generate such species.

**Figure 17:**
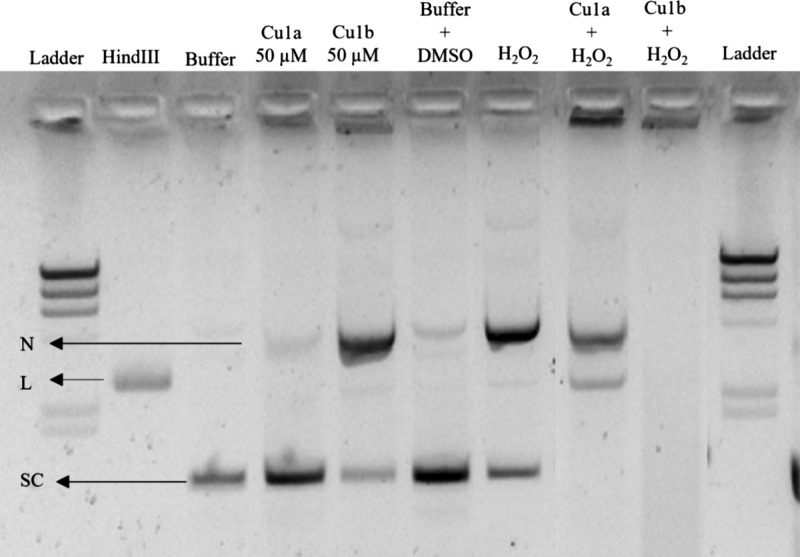
Gel electrophoresis was conducted using a 1.0% agarose gel in TAE 1× buffer with 0.0015% Gel Red, with a current of 80 V for 90 min. This aimed to determine the pDNA cleavage mechanisms after incubation with Cu(II) complexes for 24 h at 37 °C. The legend for the gel is as follows: Ladder: Molecular weight marker lambda *Hin*dIII; *Hin*dIII: pUC18 sample exposed to *Hin*dIII activity for 2 h; Buffer: pUC18 sample exposed to 5 mM Tris-HCl and 50 mM NaCl pH = 7.2 buffer for 24 h; Buffer + DMSO: pUC18 sample exposed to 5 mM Tris-HCl and 50 mM NaCl pH = 7.2 buffer with 1% (v/v) DMSO for 24 h; **Cu1a** 50 μM: pUC18 sample exposed to 50 μM of complex **Cu1a**; **Cu1b** 50 μM: pUC18 sample exposed to 50 μM of complex **Cu1b**; H_2_O_2_: pUC18 sample exposed to 100 μM H_2_O_2_; **Cu1a** + H_2_O_2_: pUC18 sample exposed to 50 μM of complex **Cu1a** and 100 μM H_2_O_2_; **Cu1b** + H_2_O_2_: pUC18 sample exposed to 50 μM of complex **Cu1b** and 100 μM H_2_O_2_. Black arrows represent the identified isoforms of pUC18 on the gel: N, nicked circular isoform; L, linear isoform of pUC18; SE, supercoiled isoform.

As depicted in Figure 17, the ability of hydrogen peroxide (H_2_O_2_) alone to act on pDNA, cleaving it, is observed as expected.^71^ This is evidenced by a decrease in the supercoiled form and an increase in the intensity of the band corresponding to the circular isoform. A faint line of the linear isoform is also visible (Figure 17). When **Cu1a** was incubated with H_2_O_2_, pDNA cleavage increased compared to H_2_O_2_ alone with a disappearance of the supercoiled isoform, and the appearance of the linear and circular (nicked) isoforms (Figures 17 and S23). This result shows an additive effect on the action of H_2_O_2_ when combined with the **Cu1a** complex (Figure S23). Regarding **Cu1b** complex incubation with H_2_O_2_, a complete disappearance of pDNA isoforms is observed, which could be due to the complete oxidation of pDNA, and only a band of the pDNA-**Cu1b** complex is observed near the well (Figure 17). These results are corroborated by literature where it was described that Cu(II) complexes are able to cleave pDNA through the oxidative pathway.^94,95^

To sum up, the Cu(II) complexes that enter the nuclear fraction (9.5 and 5.9% of complexes **Cu1a** and C**u1b**, respectively) (Table 7) are able to interact with DNA by groove binding (as obtained for calf-thymus DNA interaction (Figures 13 and 14) and cleave it due to ROS generation (oxidative mechanism via singlet oxygen radicals (^−1^O_2_) (Figures 15–17) leading to cell cycle arrest and premature senescence of HCT116-DoxR cells. On the other hand, as a high % of complexes do colocalize with the cytosol and mitochondria (Table 7), the simultaneous induction of ROS, activation of BAX, and depolarization of mitochondrial membrane may trigger autophagy and intrinsic apoptosis of HCT116-DoxR cells.

### Cell Migration Assay

**Figure 18:**
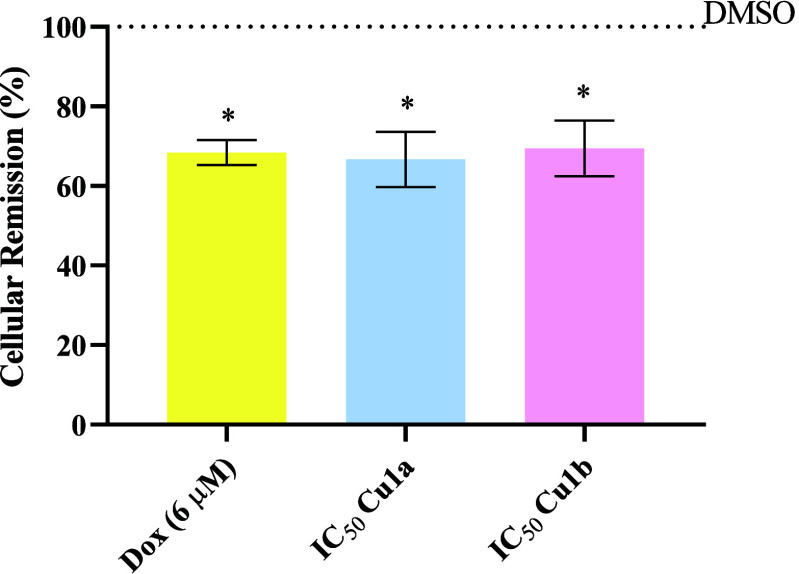
Dermal fibroblasts exposed to IC_50_ concentrations of complexes **Cu1a** and **Cu1b** for 24 h at 37 °C. 0.1% (v/v) DMSO and Dox were used as controls. The provided results are shown as mean ± SEM obtained from at least two independent biological assays. The Student’s *t* test method was used to assess the statistical significance of these results compared to the DMSO-treated control group (* *p* ≤ 0.05, ** *p* ≤ 0.005).

Cellular migration is the individual movement of cells from one location to another.^96^ This migratory process is an integral part of both the metastatic process and wound healing.^54^ In this context, the influence of **Cu1a** and **Cu1b** on cell migration was analyzed as part of the investigation of these complexes as antimetastatic agents. The in vitro wound healing assay is highly used to understand how the complexes affect cellular migration by measuring the closure of a gap previously created in the cell culture plate. Primary dermal fibroblasts, a cell model associated with the wound healing process, were exposed to concentrations equal to the IC_50_ concentrations of complexes **Cu1a** and **Cu1b** for 24 h at 37 °C. Additionally, cells were subjected to the vehicle control of 0.1% DMSO and the positive control of Dox (6 μM), and the percentage of remission/regeneration was calculated (Figure 18).

Figure 18 clearly shows that after 24 h of incubation, the DMSO vehicle control exhibited a complete remission of the wound (100%) (Figure S24). In contrast, complex **Cu1a** induced a much lower percentage of regeneration, with approximately 66% remission, and complex **Cu1b** a 69% remission when compared to the DMSO control (Figure 18), values compared with the antitumor drug Dox (68% cellular regeneration). These results suggest that both complexes **Cu1a** and **Cu1b** can inhibit cellular migration, adding their value as cytotoxic, cytostatic, and antimetastatic, which is important in a therapeutic context. Indeed, although the complexes have demonstrated the ability to inhibit cellular migration, it is crucial to consider that their use in advanced stages of tumors may face obstacles. The complexity of the tumor microenvironment, interactions with other cellular components, and conditions in *in vivo* models differ substantially from the 2D cellular conditions used.

These results align with findings in the literature where ruthenium (Ru(II)) and manganese (Mn(II)) metal complexes have the capability to decrease cellular remission, as reported previously.^54,73^

### Evaluation of In Vivo Angiogenic Potential

**Figure 19:**
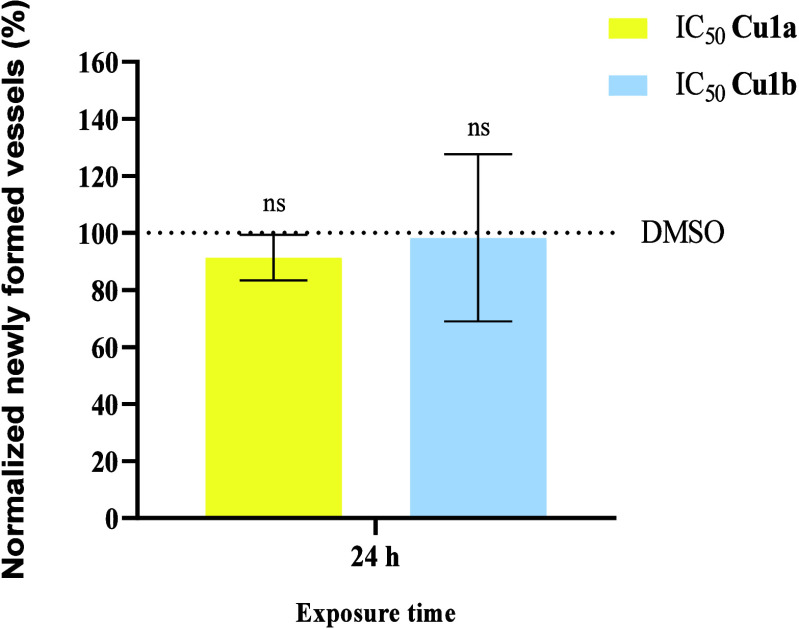
Evaluation of the angiogenic potential of Cu(II) complexes in chicken eggs exposed to complexes **Cu1a** and **Cu1b**, as well as 0.1% (v/v) DMSO in PBS 1× controls. The values were normalized to the number of tertiary veins obtained after exposure to the control (DMSO) and to the number of tertiary veins obtained in the CAM area corresponding to 0 h of incubation in the same embryo. The concentrations of the complexes used were equal to the IC_50_ concentrations of complexes **Cu1a** and **Cu1b**.

The *ex-ovo* Chorioallantoic Membrane (CAM) assay has become a preclinical model of choice for assessing the cytotoxicity of Cu(II) complexes and in vivo angiogenesis.^38^ The CAM is an extraembryonic membrane formed by mesodermal layers, highly vascularized despite being compromised, making it ideal for studying angiogenesis and antiangiogenesis in response to biomolecules and drugs.^97^ Therefore, the development of drugs with potential antiangiogenic properties represents a possibility for more effective cancer treatment. In these assays, highly vascularized regions surrounded by O-rings on the chorioallantoic membrane of chicken embryos were studied. The delimited areas were exposed to complexes **Cu1a** and **Cu1b**, as well as to the control of 0.1% (v/v) DMSO in PBS 1x. Eight embryos were used as biological replicates. Images were taken immediately after exposure to the complexes and control (0 h) and 24 h later. The values were normalized to the number of tertiary veins after exposure to the DMSO control and are represented in Figures 19 and S25.

Complexes **Cu1a** and **Cu1b** did not show significant pro- or antiangiogenic properties. Based on these results, we can conclude that the studied Cu(II) complexes do not interfere with the formation of new blood vessels (Figures 19 and S25).

Notably, none of the complexes induced the death of any of the embryos during the analyzed period, indicating the absence of in vivo toxicity. These results are similar to those obtained for other complexes, such as ruthenium (Ru(II)) complexes.^54^ Therefore, although exposure to the complexes led to a loss of viability in the HCT116-DoxR cell line, it did not trigger the death of the studied embryo. The lack of in vivo toxicity is a positive finding, especially considering the importance of evaluating the effects of the complexes in more complex biological systems than cell cultures. These results suggest that the studied complexes may have potential as therapeutic agents without causing significant adverse effects in vivo for the tested concentrations.

## Conclusions

Cu(II) complexes with terpys and dtpys substituted by *n*-naphtyl (*n* = 1, 2) and methoxy-naphtyl were synthesized, and their molecular structures were characterized via elemental analysis, HR-MS spectrometry, FT-IR and UV–vis spectroscopy, and single crystal X-ray analysis. The structural studies confirm that the geometry around the metal ion is expectedly square pyramidal. For the complexes with 1-naphtyl-substituted terpy and dtpy (**Cu1a** and **Cu2a**), the dihedral angle between the central pyridine ring and pendant substituent is in the range 40–70°, whereas 2-naphtyl-substituted complexes (**Cu1b**, **Cu1d, Cu2b** and **Cu2d**) are near coplanar.

The assessment of antiproliferative potential revealed that the most promising complexes were **Cu1a** and **Cu1b**, as they showed high antiproliferative potential in tumor cell lines while exhibiting low cytotoxicity in fibroblasts. Notably, among the three tumor cell lines, their antiproliferative potential was particularly highlighted in the HCT116-DoxR cell line with IC_50_ values of 0.24 and 0.29, respectively. As expected, in 3D spheroid models of HCT116-DoxR, the IC_50_ values for complexes **Cu1a** and **Cu1b** were higher than those obtained in 2D, approximately 50× and 116×, respectively, as reported in the literature, largely due to the increased complexity of the tumor microenvironment, approaching results expected in in vivo models. Indeed, incubation of 3D tumor spheroids with concentrations of 4.8 and 5.8 μM over 72 h and CellTox green agent led to increased death of HCT116-DoxR cells and a destabilization of the 3D structure. Understanding the mechanism of action of these complexes is crucial to comprehend how they affect tumor cells at the molecular level. Flow cytometry assays were conducted to analyze the cell death mechanisms activated by these complexes. It was observed that **Cu1a** and **Cu1b** complexes induce an increase in cells in early apoptosis, with virtually no necrosis, as well as autophagy. Furthermore, analysis of pro- and antiapoptotic protein levels, such as BAX and BCL-2, confirmed that these complexes trigger apoptosis through the intrinsic pathway, as well as the loss of mitochondrial membrane potential due to increased expression of the pro-apoptotic protein BAX. These findings are in accordance with the fact that **Cu1a** and **Cu1b** complexes can be internalized in the cells after 3 h and mostly accumulate in the mitochondria (around 30%) and cytosol (around 60%) and in a lower % in the nuclear fraction. These complexes are capable of inducing ROS through oxidative mechanisms via singlet oxygen, as described in the literature for other copper complexes. Their accumulation in those specific subcellular localizations such as cytosol, mitochondria, and nucleus their oxidative potential may trigger the induction of different types of cell death namely, autophagy, intrinsic apoptosis via BAX, and senescence due to genotoxic stress and cell cycle arrest. Besides their cytotoxic and cytostatic potential, we have also shown that both complexes have an antimetastatic potential, leading to a decrease in the ability of cell migration. Despite that these complexes did not show any significant pro- or antiangiogenic properties, as evidenced by a CAM assay, they did not show any *in vivo* toxicity, highlighting their suitability for additional preclinical investigations using alternative in vivo models and another future study before application and translation into clinical practice. Additionally, as both Cu(II) complexes are also accumulating in the cytosol, we have used bovine serum albumin as a model protein to further access the probability of Cu(II) complexes to further interact with cytosolic proteins. Moreover, the use of albumin as a model has also an additional interest as albumin is highly present in the human plasma, and this interaction can be further explored for drug delivery in vivo, providing information about the potential biodistribution and bioavailability of the complexes in living organisms. Considering this data collectively, the two copper complexes demonstrated promising antiproliferative capabilities in HCT116-DoxR cells.

## Experimental Section

### General Information

The CuCl_2_·2H_2_O was purchased from Sigma-Aldrich. Synthesis of ligands **L1a**–**d** and **L2a**–**d** was carried out according to the method described previously.^24,39−42^ All other commercially available reagents used for the synthesis were used as received. The reagent grade and spectroscopy grade solvents were used for synthesis and for spectroscopy studies, respectively.

The purity of the studied compounds is >95% that was confirmed by elemental analysis, UPLC, and HRMS. Additionally, for **Cu1a–b**, **Cu1d,** and **Cu2a–d,** molecular structures were confirmed by X-ray analysis.

### Physical Measurements

Infrared spectra were performed with the KBr technique on a Nicolet iS5 FT-IR spectrophotometer in the range of 4000–400 cm^–1^. Elemental analyses (C, H, N) were recorded on a Perkin–Elemer CHN–2400 analyzer. HR-ESI-MS analyses were performed on a maXis Impact Q-TOF mass spectrometer (Bruker Daltonics) or a Xevo G2 Q-TOF mass spectrometer (Waters Corporation, USA), both with an ESI ion source operating in positive-ion mode. Samples were diluted in LC-MS grade acetonitrile. The accurate mass and composition for the molecular ion adducts were calculated using Data Analysis 4.1 software (Bruker, Germany) or MassLynx software (Waters). UPLC was performed on a Waters Acquity UPLC system (Waters), with autosampler and diode-array detector (PDA) and a Waters Acquity UPLC BEH C8 column (2.1 mm × 100 mm, 1.7 μm particle size, temperature maintained at 30 °C). The mobile phase consisted of acetonitrile–water (90:10, v/v), while the samples were solubilized in 100% water. The flow rate was 0.30 mL/min, the injection volume was 2 μL and the duration of the run was 3 min. The eluted compounds were monitored in the range 190–400 nm. Electronic absorption spectra were carried out on an Evolution 220 UV–vis spectrophotometer in the range of 1000–250 nm in dimethyl sulfoxide and Dulbecco’s Modified Phosphate Buffered Saline (PBS, pH 7.4, Sigma-Aldrich) solutions (*c* = 2.5 × 10^–5^ mol/dm^3^). The photoluminescence spectra were performed on the Hitachi F-7000 spectrofluorometer.

### X-ray Studies

Single crystal X-ray diffraction measurements were conducted on an Oxford Diffraction Gemini A Ultra diffractometer with an Atlas CCD detector and Mo Kα radiation (λ = 0.71073 Å) at room temperature. Diffraction data collection, cell refinement, and data reduction were carried out using the CrysAlis^Pro^ software.^98^ The structure solution was performed with the use of Olex2 software^99^ and the intrinsic phasing method of the *SHELXT* package.^100^ The structure refinement was carried out by full-matrix least-squares method on *F*^2^ using SHELXL-2014.^101,102^ Non-hydrogen atoms were refined anisotropically, and hydrogen atoms were placed in calculated positions and refined with riding constraints: *d*(C–H) = 0.93 Å, *U*_iso_(H) = 1.2 *U*_eq_(C) (for aromatic) and *d*(C–H) = 0.96 Å, *U*_iso_(H) = 1.5 *U*_eq_(C) (for methyl and water). The methyl groups were allowed to rotate about their local 3-fold axis. In structures **Cu1d**, **Cu2a**, **Cu2b**, **Cu2c**, and **Cu2d**, where a reasonable solvent disorder model could not be built due to severe disorder of solvent molecules, the OLEX2 solvent mask command was employed.^44^ To prepare molecular graphics, the Mercury software package was used.^103^ Details of the crystallographic data collection, structural determination, and refinement for **Cu1a**, **Cu1b**, **Cu1d**, **Cu2a**, **Cu2b**, **Cu2c**, and **Cu2d** are given in Tables S2–S7, ESI.

### Preparation of Copper(II) Compounds of General Formula [CuCl_2_(L^*n*^)]

The copper(II) compounds were prepared according to the method described in ref (22). The 10 cm^3^ of methanolic solution of CuCl_2_·2H_2_O (0.1 g, 0.56 mmol) was added dropwise to an equimolar solution of ligand **L1a**–**d** and **L2a**–**d** (0.56 mmol) in methanol/dichloromethane mixture. The resulting reaction mixture was stirred at room temperature for 4 h and then evaporated in a hood at room temperature. After a few days, a precipitate was isolated by filtration and recrystallized from methanol.

(**Cu1a**) Yield: 85%. C_25_H_17_Cl_2_CuN_3_·2CH_3_OH: C: 58.12; H: 4.52; N: 7.53, exp. C: 57.91; H: 4.064; N: 7.063.

IR (KBr; cm^–1^): 3423 ν_(OH)_, 3044, 2923, 2850 ν_(ArH)_; 1603, 1552 ν_(C=N, C=C)_; 1474, 1416 δ_(C–CH out of the plane)_; 1247 ν_(C–N)_; 1160, 1105, 1020 δ_(C–CH in the plane)_; 869 δ_(C–C out of the plane)_, 790, 747 δ_(C–C out of the plane)_.

(**Cu1b**) Yield: 81%. C_25_H_17_Cl_2_CuN_3_·1.5H_2_O: C: 57.64; H: 3.87; N: 8.07, exp. C: 58.01; H: 3.864; N: 7.819.

IR (KBr; cm^–1^): 3417 ν_(OH)_, 3053, 2912, 2848 ν_(ArH)_; 1615, 1560 ν_(C=N, C=C)_; 1475, 1442, 1416 δ_(C–CH out of the plane)_; 1297, 1251 ν_(C–N)_; 1156, 1018 δ_(C–CH in the plane)_; 925, 868 δ_(C–C out of the plane)_, 817, 790, 753, 743 δ_(C–C out of the plane)_.

(**Cu1c**) Reported previously.^24^

(**Cu1d**) Yield: 78%. C_26_H_19_Cl_2_CuN_3_O · 0.5CH_3_OH: C: 58.95; H: 3.92; N: 7.78, exp. C: 59.026; H: 3.898; N: 7.719.

IR (KBr; cm^–1^): 3403, 3278 ν_(OH)_, 3051, 2984, 2918, 2842, 2815 ν_(ArH)_; 1604, 1560 ν_(C=N, C=C)_; 1476, 1433, 1397, 1378, 1342 δ_(C–CH out of the plane)_; 1280, 1211, 1166, 1125 ν_(C–N)_; 1019 δ_(C–CH in the plane)_; 927, 865 δ_(C–C out of the plane)_, 790δ_(C–C out of the plane)_.

(**Cu2a**) Yield: 86%. C_21_H_13_Cl_2_CuN_3_S_2_·1.5CH_3_OH: C: 48.78; H: 3.46; N: 7.58, exp. C: 49.04; H: 3.323; N: 7.365.

IR (KBr; cm^–1^): 3362 ν_(OH)_, 3125, 3028 ν_(ArH)_; 1603, 1545 ν_(C=N, C=C)_; 1487, 1450, 1356, 1329 δ_(C–CH out of the plane)_; 1250, 1194 ν_(C–N)_; 1019 δ_(C–CH in the plane)_; 905, 890 δ_(C–C out of the plane)_, 814, 787, 734δ_(C–C out of the plane)_.

(**Cu2b**) Yield: 75%. C_21_H_13_Cl_2_CuN_3_S_2_·H_2_O: C: 48.14; H: 2.89; N: 8.02, exp. C: 48.50; H: 3.034; N: 7.846.

IR (KBr; cm^–1^): 3422 ν_(OH)_, 3076, 2978, 2915, 2842 ν_(ArH)_; 1603, 1575 ν_(C=N, C=C)_; 1514, 1483, 1449, 1332 δ_(C–CH out of the plane)_; 1283, 1233, 1198 ν_(C–N)_; 1090, 1016 δ_(C–CH in the plane)_; 877, 833 δ_(C–C out of the plane)_, 774, 710δ_(C–C out of the plane)_.

(**Cu2c**) Yield: 81%. C_22_H_15_Cl_2_CuN_3_OS_2_·6H_2_O: C: 41.03; H: 4.23; N: 6.52, exp. C: 41.13; H: 4.09; N: 6.928.

IR (KBr; cm^–1^): 3422 ν_(OH)_, 3076, 2976, 2915, 2842 ν_(ArH)_; 1602, 1575 ν_(C=N, C=C)_; 1513, 1483, 1449, 1371, 1333 δ_(C–CH out of the plane)_; 1233, 1199 ν_(C–N)_; 1091, 1016, 991 δ_(C–CH in the plane)_; 877, 833 δ_(C–C out of the plane)_, 774, 711 δ_(C–C out of the plane)_.

(**Cu2d**) Yield: 84%. C_22_H_15_Cl_2_CuN_3_OS_2_ · CH_3_OH· 0.5H_2_O: C: 47.41; H: 3.36; N: 7.37, exp. C: 47.81; H: 2.966; N: 7.041.

IR (KBr; cm^–1^): 3423 ν_(OH)_, 3044, 2924, 2851 ν_(ArH)_; 1603, 1552 ν_(C=N, C=C)_; 1475, 1416 δ_(C–CH out of the plane)_; 1247 ν_(C–N)_; 1160, 1020 δ_(C–CH in the plane)_; 869 δ_(C–C out of the plane)_; 790, 747, δ_(C–C out of the plane)_.

### Biological Studies

#### Cell Culture and Maintenance

The HCT116 cancer cell line (colorectal carcinoma), A2780 cancer cell line (ovarian carcinoma), and the normal human primary dermal fibroblasts were obtained from the American Type Culture Collection (ATCC, Manassas, VA, USA). The Doxorubicin-resistant HCT116 cell line (HCT116-DoxR) was derived from the sensitive HCT116 cell line, as described by Pedrosa et al.^56^ The cell lines HCT116, HCT116-DoxR and fibroblasts were cultured in Dulbecco’s modified Eagle medium (DMEM) and A2780 was cultured in Roswell Park Memorial Institute (RPMI) medium. Both culture media were supplemented with 10% (v/v) fetal bovine serum (FBS) and 1% (v/v) Pen/Strep (Penicillin/Streptomycin) solution. HCT116-DoxR cell medium was additionally supplemented with 3.6 μM Doxorubicin (Dox) to maintain drug resistance. All media and supplements were purchased from Thermo Fischer Scientific (Waltham, Massachusetts, USA).

Cells were maintained in an incubator set at 37 °C with 5% (v/v) CO_2_ and 99% (v/v) relative humidity (SANYO CO_2_ Incubator, Electric Biomedical Co., Osaka, Japan) in 25/75 cm^2^ T-flasks (SPL Life Sciences, South Korea).

#### Cell Viability Assays in 2D Models

Cellular viability assays were performed using HCT116, HCT116-DoxR, and A2780 cancer cell lines as well as normal human primary fibroblasts. Cells were seeded at a density of 0.75 × 10^5^ cells/mL in 96-well plates and incubated at 37 °C with 5% (v/v) CO_2_ for 24 h. After this incubation period, the copper complexes were accurately weighed and immediately dissolved in 100% (v/v) dimethyl sulfoxide (DMSO). Subsequently, the complexes were diluted to the final concentration using a cell culture medium. The culture medium in each well was replaced with fresh medium containing varying concentrations of the complexes (ranging from 0.01 to 50 μM). Negative controls contained culture medium with 0.1% (v/v) DMSO, and positive controls contained 0.4 μM Dox.

After an additional 48 h of incubation under the same conditions, cellular viability was assessed using the CellTiter 96 Aqueous One Solution Cell Proliferation Assay kit (Promega, Madison, USA). Metabolically active cells contain mitochondrial dehydrogenases that can reduce 3-(4,5-dimethylthiazol-2-yl)-5-(3-carboxymethoxyphenyl)-2-(4-sulfophenyl)-2H-tetrazolium, the inner salt (MTS) to formazan. The absorbance of formazan, measured at 490 nm in a microplate reader (Tecan Infinite M200, Tecan, Männedorf, Switzerland), is directly proportional to the number of viable cells.^104^

The cell viability versus concentration graphics were analyzed using Prism 8 software (GraphPad) to determine the half-maximal inhibitory concentration (IC_50_) of each complex for the respective cell line. Additionally, the selective index (SI) for each complex was calculated by dividing the IC_50_ of human fibroblasts by the IC_50_ values of the tested cancer cell lines.

#### 3D Spheroid Formation and Cell Viability Assays

HCT116-DoxR cells were seeded at 5 × 10^4^ cells/mL in super low Attachment 96-well plates (NunclonSphera U-Shaped-Bottom Microplate, Thermo Fisher Scientific, Waltham, MA, USA) and incubated at 37 °C in a humidified atmosphere with 5% (v/v) CO_2_, to allow spheroids formation and growth.^60,105^ To perform the cell viability study, two methods were used: the MTS assay and the CellTox assay.

For MTS assay, HCT116DoxR spheroids grown for 6 to 8 days were used. On the sixth day, the culture medium was replaced with a medium containing copper complexes at desired concentrations. The spheroids were then incubated for 48 h at 37 °C under a humidified atmosphere with 5% (v/v) CO_2_. After the incubation period, the medium was replaced by a mixture containing the MTS reagent and DMEM medium (20:80). The spheroids were further incubated for 4 h and subsequently transferred the medium to a 96-well flat-bottom plate for analysis using the Tecan Infinite M200 microplate reader (Tecan, Männedorf, Switzerland).

For the CellTox assay, HCT116DoxR spheroids grown for 6 to 9 days were used. On the sixth day, the culture medium was replaced with a medium without *phenol red* containing copper complexes at 20× IC_50_ obtained for the 2D cell lines and CellToxGreen Cytotoxicity Assay, according to the manufacturer’s instructions. CellTox Green dye is an asymmetric cyanine dye that enters cells with compromised membrane integrity and binds to DNA, which enhances its fluorescence. Since it does not enter viable cells, the fluorescence obtained is directly proportional to cell death. Therefore, higher levels of fluorescence are associated with a higher number of death cells.^106^ As a negative control, spheroids were incubated with DMSO, under the same conditions. After 3, 24, 48, and 72 h of incubation, fluorescence images were acquired with Ti–U Eclipse inverted microscope (Nikon), with a FITC filter (excitation at 465–495 nm, in the blue region, and emission at 515–555 nm, in the green region).

The software ImageJ was used for fluorescence quantification in which the Corrected Total Cell Fluorescence (CTCF, eq 6) was determined. To normalize fluorescence by spheroids’ size, the CTCF values were divided by the area of the spheroids.
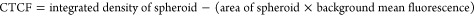
6

#### Stability Measurements

Copper complex stability in a biological medium was analyzed through UV–vis spectroscopy spanning a range from 220 to 800 nm. Complexes were dissolved in 100% (v/v) DMSO and subsequently diluted with RPMI medium (without *phenol red*) to achieve a final concentration of 50 μM. The measurements were conducted in a quartz cuvette with a 1 cm path length. Spectral analyses were obtained after 0, 3, 6, 24, and 48 h on the Evolution 300 UV–vis spectrophotometer (Thermo Fisher Scientific in Waltham, MA, USA).

#### ICP-AES (Inductively Coupled Plasma–Atomic Emission Spectrometry): Evaluation of Complex Internalization and Subcellular Localization

To evaluate the internalization and subcellular localization of copper(II) complexes in the HCT116-DoxR cell line, inductively coupled plasma-atomic emission spectroscopy (ICP-AES) was used. Cytosolic, mitochondrial, and nuclear cell fractions were obtained by using the Abcam Standard (ab109719) Cell Fractionation Kit. Cells were seeded at a cell density of 4 × 10^6^ cells/T-flask in 25 cm^2^ T-flasks. The cells were incubated for 24 h under the conditions mentioned before. After this time, the medium was replaced with a solution of complete DMEM containing 10x the IC_50_ concentrations of copper complexes or 0.1% (v/v) DMSO (vehicle control). The cells were incubated under the same conditions for 6 h. Later, the culture medium was collected, and cells were washed with PBS 1×, also recovered in 15 mL Falcon tubes. The recovered solutions were centrifuged at 800 × *g* for 5 min, and the supernatants were transferred to a new 15 mL tube. The cells in the wells were trypsinized with TrypLE Express and centrifuged at 500 × *g* for 5 min. The resulting supernatant was recovered, and the pellet was resuspended in buffer A to a cell density of 6.6 × 10^6^ cells/mL. The different cellular fractions were obtained by a series of centrifugations in specific buffers provided by the kit following the manufacturers’ instructions.

To evaluate the internalization of the complexes through time, the cells were incubated under the same conditions for 3 and 6 h. Later, the culture medium was collected, and cells were washed with PBS 1×, also recovered in 15 mL Falcon tubes. The recovered solutions were centrifuged at 800 × *g* for 5 min and the supernatants were transferred to a new 15 mL tube. The cells in the wells were trypsinized with TrypLETM Express and centrifuged at 500 × *g* for 5 min. Freshly prepared aqua regia was added to supernatants and cellular pellets, and every sample was incubated at RT overnight in a hood fume before the samples were analyzed. The quantification of the copper levels was evaluated by ICP-AES through a contracted service (Laboratório de Análises, serviço de espectroscopia de emissãoatómica, Departamento de Química, FCT-UNL).

#### Evaluation of Apoptosis Induction via Flow Cytometry

The Annexin V-FITC Apoptosis Detection Kit (catalog no. V13245, Invitrogen, USA) was used in this study for the induction of apoptosis analysis. HCT116-DoxR cells were seeded in 6-well plates at a cell density of 1 × 10^5^ cells/mL and incubated for 24 h, under the same conditions mentioned before. Then, the medium was replaced with fresh medium containing the IC_50_ concentrations of copper complexes or the negative control (0.1% (v/v) DMSO), and the two positive controls 6 μM Dox and 5 μM cisplatin (Cis) for 48 h. After this incubation period, the cells were washed with PBS 1× and trypsinized with TrypLE Express (Invitrogen). The supernatant was discarded, and the pellet was washed with PBS 1×. The cells were resuspended in 1× Annexin V-FITC binding buffer. Subsequently, the samples were incubated at room temperature (RT) with Alexa Fluor 488 annexin V and 100 μg/mL of propidium iodide (PI) in the dark for 15 min. After incubation, 1× Annexin V-FITC binding buffer was added and the samples were analyzed using an Attune Acoustic Focusing Flow Cytometer (Life Technologies, Carlsbad, USA). Results were processed with Attune Cytometric software.

#### Evaluation of Mitochondrial Membrane Potential (ΔΨ_m_) via Flow Cytometry

The mitochondrial membrane potential (ΔΨ_M_) was evaluated by using the JC-1 Mitochondrial Membrane Potential Assay Kit (Abnova Corporation, Walnut, California, USA). Initially, HCT116-DoxR cells were cultured in 6-well plates at a cell density of 1 × 10^5^ cells/mL and incubated for 24 h. After this incubation period, the medium was replaced with medium with the copper complexes at the IC_50_ concentration and incubated for 48 h. As a negative (vehicle) control, 0.1% (v/v) DMSO was used, while 6 μM Dox and 5 μM Cis were used as positive controls. Following incubation, the culture medium was removed, and cells were washed with PBS 1×, detached with TrypLE Express, and washed again with PBS 1×. Cells were later incubated for 20 min in the dark at 37 °C with a solution composed of *phenol red*-free DMEM containing 5% (v/v) FBS and the JC-1 probe. Subsequently, the samples were centrifuged, and the pellet was resuspended in *phenol red*-free DMEM with 5% (v/v) FBS. The samples were then analyzed in the Attune Acoustic Focusing Flow Cytometer (Life Technologies, Carlsbad, CA, USA) and results were processed with Attune Cytometric software.

#### Quantification of BAX and BCL-2 Protein Expression via Western Blot

HCT116-DoxR cells were seeded in a 25 cm^2^ T-flask at a density of 2 × 10^6^ cells/T-flask. After 24 h incubation, under the conditions mentioned before, the culture medium was replaced with a medium containing the IC_50_ concentrations of complexes Cu1a and Cu1b or 0.1% (v/v) DMSO (control). Cells were incubated for 48 h and later were washed and collected using cold PBS 1× and a cell scraper. The samples were centrifuged for 5 min at 700 × *g* (Sigma 3–16K Sartorius, Germany), and the resulting pellets were resuspended in 50 μL of fresh lysis solution (150 mM NaCl; 50 mM Trs-HCl pH = 8; 5 mM ethylenediaminetetraacetic acid, EDTA; 1× protease inhibitors, Complete ULTRA tablets, mini, eazypack, Roche; 1× phosphatase inhibitors, PhosStop, Roche; 2% NP-40, Thermo Fisher Scientific, Waltham, MA, USA; 0.1% 1,4-dithiothreitol (v/v), DTT, Amresco, USA; 1 mM phenylmethylsulfonyl fluoride, PMSF, Sigma, St. Louis, USA). Subsequently, the samples were stored for a minimum of 2 h at −80 °C.

After the storage period, the samples were submitted 5 ultrasound (Elma sonicator, D-78224 Singen/Htw, Germany) cycles on ice (2 min and 30 s on ultrasound followed by 1 min period on ice) and then centrifuged at 10,000 × *g* for 5 min. The supernatant was transferred to a 1.5 mL Eppendorf tube, and the total protein extracted was quantified with Pierce 660 nm Protein Assay kit (ThermoFisher Scientific, MA, USA).

In the SDS-PAGE analysis of BAX and BCL-2 proteins, 20 μg of protein was applied into a 10% polyacrylamide gel as well as NZYColour protein marker II and transferred to a 0.45 μm polyvinylidene fluoride (PVDF) membrane (GE Healthcare Life Sciences, Germany). To block nonspecific interactions, a blocking solution containing 5% (w/v) skim milk in TBST 1x buffer (50 mM Tris-HCl pH 7.5, 150 mM NaCl, and 0.1% (v/v) Tween 20) was utilized. The membrane was incubated for 1 h at room temperature with agitation. Subsequently, it was incubated again for 1 h with agitation in TBST 1x solutions containing 5% (w/v) nonfat milk and different primary antibodies (anti-Bax, dilution 1:5000, Abcam, United Kingdom; and anti-BCL-2, dilution 1:1000, Sigma, St. Louis, USA). After incubation, the membrane was washed 3 times with TBST 1× for 5 min each under constant agitation. The same procedure was repeated for the secondary antibodies (Anti-rabbit, 1:2000, Cell signaling; Antimouse, 1:3000, Cell signaling). To identify the protein bands, membranes were treated with Western Bright ECL substrate (Advansta, USA) for 5 min, and the film was exposed to the membrane in a dark room. Membranes were later incubated two times with Stripping buffer (0.1 M glycine, 20 mM magnesium acetate, 50 mM KCl, pH 2.0) during 10 and 20 min, respectively, under agitation. Then membranes were incubated as described above, but using β-actin antibody (1:5000; Sigma, St. Louis, USA) as the primary antibody (control for results normalization). Protein quantification was done by densitometry with ImageJ software.

#### Evaluation of Autophagy Induction by Flow Cytometry

Cell death via autophagy was evaluated using the Autophagy Assay Kit (ab139484; Abcam, Cambridge, United Kingdom), according to the manufacturers’ instructions. HCT116-DoxR cells were seeded at a density of 1 × 10^5^ cells/mL in 6-well plates for 24 h, under the same conditions described before. After this incubation period, the culture medium was replaced with a fresh medium containing the copper complexes under evaluation at their IC_50_ concentration. A 0.1% (v/v) DMSO solution was used as the negative (vehicle) control, and 6 μM Dox and 5 μM Cis were used as positive controls. The cells were incubated again for 48 h. Rapamycin (1500 nM) was also added as a positive control, and it was added 18 h before the end of the 48 h incubation. After this period, the supernatant was discarded, and each well was washed with PBS 1×. Subsequently, cells were detached using TrypLE Express and washed with *phenol red*-free DMEM medium containing 5% (v/v) FBS. Cells were incubated with Green Stain Solution in *phenol red*-free DMEM medium containing 5% (v/v) FBS for 30 min at RT. After this period, the samples were washed and then resuspended in Assay Buffer 1×. The samples were analyzed using an Attune Acoustic Focusing Flow Cytometer (Life Technologies, Carlsbad, USA), and the results were analyzed via the Attune Cytometric software.

#### Reactive Oxygen Species (ROS) Production by Flow Cytometry

The quantification of reactive oxygen species (ROS) production was performed using the probe dichlorofluorescein diacetate (H2DCF-DA) (molecular probes: reactive oxygen species (ROS) detection reagents, Invitrogen, USA). Briefly, HCT116-DoxR cells were seeded in six-well plates at a density of 1 × 10^5^ cells/mL and incubated for 24 h under the conditions mentioned before. After the incubation period, the medium was replaced with fresh medium containing the tested copper complexes at their IC_50_ concentrations. 0.1% (v/v) DMSO solution was used as the negative control, and 6 μM Dox, 5 μM Cis, and 42 μM Tertbutyl Hydroperoxide (TBHP) were used as positive controls. The cells were incubated again under the same conditions mentioned above for 48 h. Later, the culture medium was removed, cells were washed with PBS 1× and TrypLE Express to detach them and then they were washed again with PBS 1×. After that, cells were incubated in a solution of 10 μM H_2_DCF-DA in PBS 1x for 20 min at 37 °C. Subsequently, the samples were analyzed by the Attune Acoustic Focusing Flow Cytometer (Life Technologies, Carlsbad, USA), and the obtained data were processed by the respective software (Attune Cytometric software).

#### Cell Cycle Progression Analysis

HCT116-DoxR cells were cultured in six-well plates at a density of 1 × 10^5^ cells/mL and incubated at 37 °C, 5% (v/v) CO_2_, and 99% (v/v) relative humidity for 8 h. To ensure that cells were in the same phase of the cell cycle, they were subjected to a double thymidine block for synchronization. At the end of the incubation, the culture medium was replaced with 2 mM thymidine in complete medium, and cells were incubated for 16 h. After incubation, the thymidine-containing medium was replaced with a thymidine-free complete medium, and cells were incubated for an additional 8 h. After this time, a second thymidine block was applied, as described earlier. At the end of the incubation, the thymidine-containing medium was replaced with a fresh medium containing the cooper complexes at their IC_50_ concentration. A 0.1% (v/v) DMSO solution was used as vehicle control, while 6 μM Dox, and 5 μM Cis were used as positive controls. Cells were incubated again for 9, 12, 18, and 24 h. Immediately after exposure (0 h) and at the end of each incubation period, the medium was removed, cells detached with TrypLE Express, and centrifuged for 5 min at 650 × *g*, at 4 °C. The pellet was resuspended in cold PBS 1× and centrifuged at 3000 × *g* for 5 min, at 4 °C. Later, the pellet was resuspended in cold PBS 1×, and 1 mL of 80% (v/v) cold ethanol was added dropwise to each tube. The samples were stored at 4 °C for a minimum of 16 h and, after this time, the samples were centrifuged for 10 min at 7500 × *g* and 4 °C. The samples were then treated with 50 μg/mL RNase in PBS 1× and incubated for 30 min at 37 °C. Then, 100 μL of 25 μg/mL PI and 650 μL of 1× PBS were added to each sample. The DNA content was evaluated on an Attune acoustic focusing flow cytometer (Life Technologies, Carlsbad, USA), and the results were analyzed by the respective software (Attune Cytometric software).

#### Evaluation of Cellular Senescence

In this assay, a senescence assay kit (Beta Galactosidase, Fluorescence) (ab228562; Abcam, UK) was used. HCT116-DoxR cells were seeded in 24-well plates at a density of 5 × 10^5^ cells/well and incubated for 24 h, at the conditions mentioned before. After incubation, the culture medium was replaced with a medium containing the IC_50_ concentrations of copper complexes to be tested, and cells were incubated for an additional 48 h. Additionally, 0.1% (v/v) DMSO was used as a vehicle control, and 6 μM Dox and 5 μM Cis were used as positive controls. At the end of the incubation period, the medium was removed, and 500 μL of DMEM with 1.5 μL of Senescence dye was added to each well. The plates were incubated for an additional 1–2 h at 37 °C. Subsequently, the well contents were removed, and the cells were washed twice with Wash Buffer (provided by the kit). The cells were trypsinized with TrypLE Express and then centrifuged for 5 min at 500 × *g*. After centrifugation, the cells were washed with the wash buffer, centrifuged, and resuspended again in 500 μL of Wash Buffer. The samples were subsequently analyzed by an Attune acoustic focusing flow cytometer (Life Technologies, Carlsbad, USA), and the results were analyzed by the respective software (Attune Cytometric software).

#### Interaction of Cu(II) Complexes with Calf-Thymus DNA (ctDNA)

Ethidium bromide (EB) and calf-thymus DNA (ctDNA) were purchased from Sigma-Aldrich and Thermo Fisher Scientific, respectively. Prior to experiments, a solution containing 10 mg/mL of ctDNA in DNase-free and RNase-free distilled, deionized water was spectroscopically monitored at 260 nm and at 280 nm to determine the concentration and check the purity. The ratio *A*_260_/*A*_280_ equals 1.88, indicating that ctDNA was sufficiently free from protein.

The electronic spectra of complexes **Cu1a** and **Cu1b** were monitored in the absence and presence of DNA in the UV–vis region. Absorption titration experiments were conducted by maintaining the complex concentration constant (25 μM) and varying the concentration of ctDNA (0–100 μM). Blank ctDNA samples were used as a reference.

The ethidium bromide (EB) fluorescence displacement assay was carried out using a Hitachi F-7000 spectrofluorometer. An equimolar amount (50 μM) of EB solution was added to ctDNA solution at room temperature, followed by 2 h incubation in the dark. The compound was then titrated into the EB–ctDNA mixture, well mixed, and allowed to stand for 30 min. The concentrations of the complexes varied from 0 to 40 μM.

#### Interaction of Cu(II) Complexes with Plasmidic DNA (pDNA)

*Escherichia coli* transformed with plasmid DNA (pDNA) pUC18 was inoculated on an LB-agar plate (Luria–Bertani medium) (Applichem, Darmstadt, Germany) supplemented with ampicillin (100 μg/mL) (Bioline, London, UK). The inoculum was incubated for 24 h at 37 °C. Subsequently, the *E. coli* bacteria were inoculated into a liquid LB medium supplemented with ampicillin (100 μg/mL) and incubated for another 24 h at 37 °C with constant agitation. After incubation, pDNA was extracted by using the *NZYSpeedy Miniprep Kit* (NZYtech) following the manufacturers’ instructions.

Experiments were conducted with 100 ng of pUC18 DNA incubated with increasing concentrations of **Cu1a** and **Cu1b** complexes (5, 25, 50, 75, and 100 μM) or in their absence (pUC18 only or with 0.1% DMSO (v/v), both negative controls). For this assay, positive control was included, containing pUC18 DNA (100 ng) previously incubated with the *Hin*dIII enzyme to linearize the plasmid in the last 2 h of incubation. All samples were programmed to a final volume of 20 μL and incubated at 37 °C for 24 h in a buffer solution (5 mM Tris-HCl and 50 mM NaCl, pH 7.02). Electrophoresis was performed in 0.8% (w/v) agarose gel (NZYtech) in TAE 1× buffer at a constant voltage of 70 V for 80 min. The electrophoresis gel image was acquired using the Gel Doc EZ Imager (Bio-Rad), and band quantification was performed using ImageJ software.

#### Determination of the pDNA Cleavage

The pDNA (100 ng) was exposed to reactive oxygen species (ROS) scavenging agents, namely, sodium azide (NaN_3_) at 50 μM and hydrogen peroxide (H_2_O_2_) at 100 μM. All samples were prepared in a buffer solution (5 mM Tris-HCl, 50 mM NaCl, pH 7.02). Controls were also included where 100 ng of pUC18 was exposed to 0.1% (v/v) DMSO, and the activity of the *Hin*dIII restriction enzyme (linearized plasmid control) for 24 h. All the samples were adjusted to a final volume of 20 μL and incubated for 24 hs at 37 °C. The samples were analyzed through electrophoresis on 0.8 or 1.0% in TAE 1× buffer with 0.0015% Gel Red, and a constant current of 70 V was applied for 90 min. The electrophoresis gel image was acquired in Gel Doc EZ Imager (Bio-Rad) and band quantification was performed using ImageJ software.

#### Cell Migration Assay

Fibroblasts were seeded in 24-well plates at a cell density of 4 × 10^5^ cells/mL. The plates were incubated for 24 h under the same conditions described before until a confluent cell monolayer was obtained. After incubation, a scratch was made in the center of each well using a sterile 200 μL pipette tip, and the medium was replaced with medium containing the IC_50_ concentrations of the copper complexes or the respective controls of 0.1% (v/v) DMSO (vehicle control), 5 μM Cis, and 6 μM Dox, the latter two as positive controls. The cells were incubated at 37 °C for 48 h. The plates were photographed under an inverted microscope (Nikon TMS, Nikon Instruments, Tokyo, Japan) at 4× magnification (immediately after exposure to the complexes (0 h), 24, and 48 h following exposure). Using ImageJ software, the width of the scratch was measured, and based on these data, the percentage of remission of the scratch (associated with the migratory capacity of fibroblasts) was calculated.

#### In Vivo Toxicity and Angiogenic Potential

To assess the angiogenic potential of the copper complexes, an ex-ovo CAM (chick chorioallantoic membrane) *in vivo* model using chicken eggs was performed. Fertilized eggs were opened, and the embryos and blood vessels were stabilized for 24 h in an incubator at 37 °C in perforated individual weighing boxes. After incubation, 4 silicone O-rings were placed on areas with a high number of blood vessels. Each O-ring received PBS 1x with a concentration equal to the IC_50_ concentrations of the copper complexes or 0.1% (v/v) DMSO vehicle control. The area limited by each O-ring was photographed immediately after the addition of the complexes and after 24 and 48 h of incubation at 37 °C. The images were captured with a digital USB microscope camera (Opti-Tekscope OT-V1). Image analysis was performed using ImageJ software.

The *ex-ovo* CAM assay fulfills the Directive 2010/63/EU of the European Parliament for protection of animal models for scientific purposes.

#### Interaction of Cu(II) Complexes with Bovine Serum Albumin (BSA)

All solutions used to perform the BSA interaction assays were diluted in filtered buffer (10 mM phosphate buffer and 150 mM NaCl) pH 7.0, with BSA at a fixed final concentration of 20 μM and the concentrations of the complexes varying from 10 to 100 μM. Control solutions were also prepared, namely, a solution containing only BSA, a solution consisting of BSA + DMSO, and a solution consisting of DMSO only. Solutions were analyzed after 24 h of incubation at 37 °C. UV–visible absorption spectra were acquired using the Evolution 300 UV–vis (Thermo Fisher Scientific, Waltham, MA, USA) in the wavelength range between 245 and 500 nm in quartz cuvettes. The fluorescence of the samples mentioned was analyzed using a Cary Eclipse fluorimeter (VARIAN, California, USA). The BSA molecule was excited at 278 nm (maximum absorption peak), and emission was recorded between 290 and 500 nm. The setup was set at a slow speed (120 nm/min) using 5 nm slits. The absorbance value of the buffer containing DMSO (at the respective percentages) was subtracted from that of each sample.

#### Statistical Analysis

All results were expressed as mean ± SEM of at least two independent biological assays, each obtained by technical duplicates, unless otherwise specified. One-way ANOVA or Student’s *t*-test was used to determine statistical significance (*p* < 0.05) using the GraphPad Prism 8 software (GraphPad Software Inc., San Diego, CA, USA).

## Supporting Information Available

X-ray data, MS and FT-IR spectra of compounds and ligands, electronic absorption spectra, stability and solubility in DMSO, PBS and RPMI, cell viability of A2780, HCT116, HCT116-DoxR, fibroblast cell lines after exposure to different concentrations of complexes, ligands, doxorubicin, and cisplatin, Western blot assay, DNA and BSA binding studies, pDNA cleavage mechanism, and fibroblast cell migration assay (PDF)
Molecular formula strings (CSV)
Spheroid formation till the 6th day of growth (MP4)

## Accession Codes

Accession codes crystallographic data for **Cu1a**, **Cu1b**, **Cu1d**, **Cu2a**, **Cu2b**,**Cu2c**, and **Cu2d** were deposited with the Cambridge Crystallographic Data Center. CCDC Numbers: 2124050 (**Cu1a**), 2150148 (**Cu1b**), 2124051 (**Cu1d**), 2124052 (**Cu2a**), 2124054 (**Cu2b**), 2361049 (**Cu2c**), and 2124053 (**Cu2d**). The authors will release the atomic coordinates upon article publication. Copies of this information may be obtained free of charge from the Director, CCDC, 12 Union Road, Cambridge CB2 1EZ, U.K. (Fax: +44 1223 336033; Email: deposit@ccdc.cam.ac.uk or www.ccdc.cam.ac.uk)

## Abbreviations

ΔΨ_m_: mitochondrial membrane potential
A2780: ovarian carcinoma cell line
BSA: bovine serum albumin
CAM: chick chorioallantoic membrane
Cis: cisplatin
DCF: 2′,7′-dichlorofluorescein
DMEM: Dulbecco’s modified Eagle’s medium
Dox: doxorubicin
dtpy: 2,6-bis(thiazol-2-yl)pyridine
EB: ethidium bromide
FBS: fetal bovine serum
FITC: fluorescein isothiocyanate
H2DCF-DA: 2′,7′-dichlorohydrofluorescein diacetate
HCT116: colorectal carcinoma cell line
HCT116-DoxR: doxorubicin-resistant colorectal carcinoma cell line
HSA: human serum albumin
ICP-AES: inductively coupled plasma-atomic emission spectrometry
JC-1: 5,5,6,6′-tetrachloro-1,1′,3,3′ tetraethylbenzimidazolcarbocyanine iodide
PBS: phosphate buffered saline
Pen: penicillin
Phe: phenylalanine
PI: propidium iodide
PS: phosphatidylserine
PVDF: polyvinylidene fluoride
py: pyridine
ROS: reactive oxygen species
RPMI: Roswell Park Memorial Institute
SE: supercoiled form
SI: selectivity index
SPY: square-pyramid
Strep: streptomycyn
TBHP: *tert*-butyl hydroperoxide
TBY: trigonalbipyramid
terpy: 2,2′:6′,2″-terpyridine
TME: tumor microenvironment
Trp: tryptophan
Tyr: tyrosine
UV–vis: ultraviolet–visible
