# Supplementary material for: Optimization of Antiproliferative Properties of Triimine Copper(II) Complexes
Source: J Med Chem. 2024 Nov 4;67(21):19475–502. doi: 10.1021/acs.jmedchem.4c01806 (PMC11571215; doi:10.1021/acs.jmedchem.4c01806)
Supplement: Supplementary file 1 — jm4c01806_si_001.pdf [file jm4c01806_si_001.pdf]

Electronic Supporting Information

Optimization of antiproliferative properties of triimine copper(II) complexes

Katarzyna Choroba[a], Bartosz Zowiślok[a], Sławomir Kula[a], Barbara Machura[a], Anna M. Maroń[a,*], Karol Erfurt[b], Cristiana Marques[c,d], Sandra Cordeiro[c,d], Pedro V. Baptista[c,d], Alexandra R. Fernandes[c,d,*]

[a] Institute of Chemistry, University of Silesia, Szkolna 9, 40-006 Katowice, Poland

* corresponding author: Anna M. Maroń (anna.maron@us.edu.pl)

[b] Department of Chemical Organic Technology and Petrochemistry, Silesian University of Technology, Krzywoustego 4, 44-100 Gliwice, Poland

[c]Associate Laboratory i4HB - Institute for Health and Bioeconomy, NOVA School of Science and Technology, NOVA University Lisbon, 2819-516 Caparica, Portugal

[d] UCIBIO, Departamento de Ciências da Vida, NOVA School of Science and Technology, Campus de Caparica, 2829-516 Caparica, Portugal

*Corresponding author: Alexandra R. Fernandes (ma.fernandes@fct.unl.pt)

Table of Contents

Electronic Supporting Information S1

Table of Contents S2

1. Bioactivity of Copper(II) Compounds With 2,2′:6′,2″-Terpyridine Derivatives S6

 Table S1. Bioactivity of [CuX_2(4′-R-terpy)] (X = Cl, Br). S6

2. General Characterization S26

 Figure S1. HR-MS of copper(II) complexes. S32

 Figure S2. FT-IR spectra of studied Cu(II) compounds with reference to corresponding ligands. S36

 Figure S3. UPLC spectra of compounds Cu1a–Cu1d. S38

 Figure S4. Comparison of the UV-Vis spectra of aqueous solutions of Cu1a-d (black, c = 25 μM or 50μM) and the spectra extracted from the UPLC detector (green). S39

3. Crystallography S40

 Table S2. Crystal data and structure refinement. S40

 Table S3. Selected bond lengths [Å] and angles [°]. S42

 Table S4. Short intra- and intermolecular hydrogen bonds. S43

 Table S5. Short π•••π interactions. S44

 Table S6. Y–X•••Cg(J)(π-ring) interactions. S45

 Table S7. Ring-metal interactions Cg•••Me S45

 Table S8.Selected structural parameters in square pyramidal Cu(II) complexes with 4´-R-terpy and 4-R-dtpy S46

 Figure S5.Perspective view showing intermolecular interactions in Cu1a. S47

4. UV-Vis Spectra and Stability in DMSO and PBS S48

 Figure S6.Stability studies of Cu1a-b, Cu1d and Cu2a-d compounds in DMSO and PBS solutions. S54

 Figure S7. Electronic absorption spectra of compounds Cu1a, b and d and Cu2a-d (black line) in comparison to the corresponding organic ligands (red line) in DMSO solution (c – 25μM) ; inset plots: electronic absorption spectra of copper complexes in concentrated DMSO solution (c – 1mM) (green line). S55

 Figure S8a. Electronic absorption spectra of compounds Cu2a in DMSO (c – 25μM, black line), CH_3CN (c – 25μM, light blue), EtOH (c – 25μM, blue) with comparison to the corresponding organic ligands (red line) in DMSO solution (c – 25μM), inset plots: comparison of Cu2a and Cu1a absorption spectra in EtOH solutions (c – 25μM). S56

Figure S8b. Electronic absorption spectra of compounds Cu2b-d in DMSO (c – 25μM, black line), CH_3CN (c – 25μM, light blue), EtOH (c – 25μM, blue) with comparison to the corresponding organic ligands (red line) in DMSO solution (c – 25μM), inset plots: comparison of Cu2b-d and Cu1b-d absorption spectra in EtOH solutions (c – 25μM)..... S57

Table S9. Electronic absorption maxima of complexes Cu1a-b, c and Cu2a-d................ S58

5. Cell Viability Assays: HCT116, HCT116-DoxR, A2780 and Fibroblasts S59

Figure S9.Cell viability of HCT116 tumour cell line after 48 h of exposure to various concentrations of Cu(II) complexes. 0.1% (v/v) DMSO was used as a vehicle control (corresponds to the dot line). The values presented are the mean ± SEM of two independent biological assays, and statistical significance was evaluated in relation to the DMSO control using the Student t-test and One-Way ANOVA (* p-value ≤ 0.05; ** p-value ≤ 0.005; *** p-value ≤ 0.0005; **** p-value ≤ 0.0001). S59

Figure S10.Cell viability of HCT116-DoxR tumour cell line after 48 h of exposure to various concentrations of complexes. 0.1% (v/v) DMSO was used as a vehicle control. The values presented are the mean ± SEM of two independent biological assays, and statistical significance was evaluated in relation to the DMSO control using the Student t-test and One-Way ANOVA (* p-value ≤ 0.05; ** p-value ≤ 0.005; *** p-value ≤ 0.0005; **** p-value ≤ 0.0001).. S60

Figure S11.Cell viability of A2780 tumour cell line after 48 h of exposure to various concentrations of complexes. 0.1% (v/v) DMSO was used as a control vehicle. The values presented are the mean ± SEM of two independent biological assays, and statistical significance was evaluated in relation to the DMSO control using the Student t-test and One-Way ANOVA (* p-value ≤ 0.05; ** p-value ≤ 0.005; *** p-value ≤ 0.0005; **** p-value ≤ 0.0001).. S61

Figure S12. Cell viability of the healthy human Fibroblasts after 48 h of exposure to various concentrations of complexes. 0.1% (v/v) DMSO was used as vehicle control. The values presented are the mean ± SEM of two independent biological assays, and statistical significance was evaluated in relation to the DMSO control using the Student's t-test and One-Way ANOVA (* p-value ≤ 0.05; ** p-value ≤ 0.005; *** p-value ≤ 0.0005; **** p-value ≤ 0.0001).. S62

Figure S13.Viability of HCT116 cancer cell line after exposure to different concentrations of doxorubicin (A) and cisplatin (B) for 48 hours. Viability of A2780 cancer cell line after exposure to different concentrations of doxorubicin (C) and cisplatin (D) for 48 hours. Viability of Fibroblasts after exposure to different concentrations of doxorubicin (E) and cisplatin (F). 0.1% (v/v) DMSO was used as the vehicle control. Data are expressed as the mean ± SEM of three biological assays. S63

Figure S14.Cell viability of HCT116-DoxR tumour cell line after 48 h of exposure to various concentrations of ligands. 0.1% (v/v)DMSO was used as a vehicle control. The values presented correspond to the mean ± SEM of two independent biological assays, and statistical significance was assessed in relation to the DMSO control using the T-Student

method and One-Way ANOVA (* p-value ≤ 0.05; ** p-value ≤ 0.005; *** p-value ≤ 0.0005; **** p-value ≤ 0.0001). S64

Figure S15. HCT116DoxR spheroid with 6 days of growth. Scale of 200 μm. See also video for spheroid formation till the 6th day of growth. S65

Video S15. Spheroid formation till the 6th day of growth. S65

Figure S16. Cell viability in HCT116-DoxR 3D spheroids after exposure to DMSO (vehicle control) or complexes Cu1a and Cu1b and incubation with CellToxTM Green dye for 3, 24, 48 and 72 h. Fluorescence microscopy images of HCT116-DoxR spheroids exposed to (A) DMSO, (B) complex Cu1a and (C) complex Cu1b. Images represent three independent biological assays (each with at least 3 spheroids. Scale of 200 μm. S66

6. Stability and Solubility of Complexes Cu1a and Cu1b S67

Figure S17.Evaluation of the stability and solubility of complexes Cu1a ((A), (B) and (C)) and Cu1b ((D), (E) and (F)) by UV-Vis spectroscopy. Absorption spectra 50 μM of complexes Cu1a and Cu1b in RPMI culture medium (previously diluted in DMSO) at different incubation times: 0, 3, 6, 24, and 48 h, at 37°C. S67

7. Western Blot Assay - BAX and BCL-2Protein Expression S68

Figure S18.Western Blot bands utilized to quantify the expression of proteins BCL-2 and BAX in HCT116-DoxR cells after exposure to the respective negative control (DMSO) or complexes Cu1a and Cu1b. S68

8. ctDNA Interaction Studies S69

Figure S19. Stern-Volmer plot for EB-DNA adduct treated with Cu1a S69

Figure S20. Stern-Volmer plot for EB-DNA adduct treated with Cu1b S69

Figure S21. Wolfe–Shimmer's plot for Cu1a treated with increased amount of DNA S70

Figure S22.Wolfe–Shimmer's plot for Cu1b treated with increased amount of DNA S70

9. pDNA Cleavage Mechanisms S71

Figure S23. Ratio of the intensities of circular, linear and supercoiled isoforms.(A) and (B) show the quantification of the interaction of Cu(II) complexes with pDNA at increasing concentrations of the complexes (5, 25, 50, 75 and 100 μM).(C) e (D)- quantification of samples subjected to the NaN_3 sequestering agent and the H_2O_2 cleavage positive control, respectively. Incubated at 37 ºC for a period of 24 hours. S71

10. Fibroblast Cell Migration Assay S72

Figure S24. Representative images of the wound-healing assay in fibroblasts after incubation for 0 and 24 h. Inverted microscope with 4x magnification. S72

11. Evaluation of In-Vivo Angiogenic Potential S73

Figure S25. Evaluation of the complexes potentiality to modulate angiogenesis. Green channel of the same image used for counting the number of veins after 24 h exposure to (A) DMSO, (B) IC_{50} concentrations of complex Cu1a and (C) IC_{50}concentrations of complex Cu1b. Inverted microscope with 4x magnification. S73

12. Interaction with Bovine Serum Albumin (BSA) S73

Figure S26. UV-Visible absorption spectra depict the interaction between 20 μM of BSA exposed to variable concentrations of complexes (A) Cu1a and (B) Cu1b. Different interaction ratios were observed after a 24 h incubation period at 37 °C. The spectrum is normalized concerning the absorbance of the individual BSA sample to facilitate the interpretation of the interaction effects. S74

Figure S27. The fluorescence intensity spectrum represents the interaction between BSA and the complexes Cu1a and Cu1b. The BSA concentration is fixed at 20 μM, while the complex concentrations vary between 10-100 μM. The assays were conducted after a 24 h incubation at 37 °C. This spectrum is normalized based on the fluorescence of the individual protein sample to facilitate the interpretation of the interaction's influence. S76

References S77

Abbreviations S79

1. Bioactivity of Copper(II) Compounds With 2,2′:6′,2″-Terpyridine Derivatives

Table S1. Bioactivity of [CuX_2(4′-R-terpy)] (X = Cl, Br).

Type of the R substituent and X atom	IC50 (μM)/cell line) 2D	IC50 (μM)/cell line) 3D	Interaction with DNA	BSA/HSA binding	DNA cleavage	ROS generation
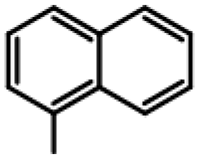 X = Cl	0.28 /HCT116<br>0.24 /HCT116-DoxR<br>0.53 /A2780<br>9.26 / Fibroblast	10.10 /HCT116-DoxR 3D spheroids	FID EB<br>(K_{app}=1.75×10^5 M^{-1})<br><br>UV-Vis titration<br>(K_b = 1.10×10^6 M^{-1})	BSA binding<br>UV-Vis titration<br>(hypochromism)	pDNA (pUC18), gel electrophoresis<br><br>increase in the circular form (N) and decrease of the supercoiled isoform (SC) after treatment with increasing concentrations (5, 25, 50, 75, and 100 μM)	staining with H2DCHF-DA in HCT116-DoxR cells<br><br>ROS production with values 1.42x higher than the DMSO control
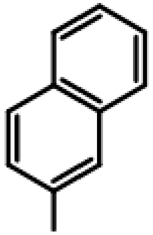 X = Cl	0.21 /HCT116<br>0.29 /HCT116-DoxR<br>0.57 /A2780<br>13.30 /Fibroblast	33.86 /HCT116-DoxR 3D spheroids	FID EB<br>(K_{app} = 2.35×10^6 M^{-1})<br><br>UV-Vis titration<br>(K_b = 0.85×10^6 M^{-1})	BSA binding<br>UV-Vis titration<br>(hypochromism)	pDNA (pUC18), gel electrophoresis<br><br>decrease in supercoiled form and increase in the intensity of the circular isoform at concentrations of 25 and 50 μM	staining with H2DCHF-DA in HCT116-DoxR cells<br><br>ROS production with values 1.48x, higher than the DMSO control

Type of the R substituent and X atom	Internalization	Apoptosis	Mitochondrial membrane potential	Western blot (protein expression)	Autophagy	Cell cycle progression	Cellular senescence	Cell migration	In vivo assays	Ref
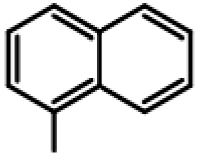<br>X = Cl	internalization rate ~81%(3h)<br><br>internalization rate ~81% (6h)	a cell survival rate of 23.1% with most cells in early apoptosis (55.2%), 21.7% in late apoptosis, and no necrosis	depolarization with a ratio of 0.79	BAX/BCL-2 1.80x higher than the control	2x-fold significant increase in autophagic vesicles	A delay in the cell cycle at S phase after 9h, in the G2/M after 12 h and a delay in the cell cycle at G0/G1 at 18 h<br><br>cytostatic effect beyond their cytotoxic effect	~2x higher quantity of senescent cells than DMSO control	66% of remission compared to the DMSO control	CAM assay<br><br>no significant pro- or anti-angiogenic properties	this work
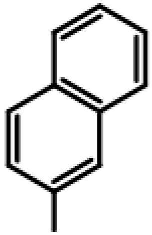<br>X = Cl	internalization rate ~65% (3h)<br><br>internalization rate ~62% (6h)	33,1% of cells in early apoptosis and 6.9% in late apoptosis	depolarization with a ratio of 0.88	BAX/BCL-2 1.65x higher than the control	2x-fold significant increase in autophagic vesicles		~1.5x higher quantity of senescent cells than DMSO control	69% remission when compared to the DMSO control		this work

Type of the R substituent and X atom	IC50 (µM)/cell line) 2D	IC50 (µM)/cell line) 3D	Interaction with DNA	BSA/HSA binding	DNA cleavage	ROS generation	Internalization	Apoptosis	Mitochondrial membrane potential	Ref
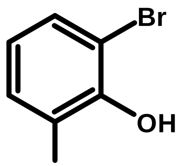<br>X = Cl	1.24 /MGC80-3<br>0.95 /T-24<br>1.19 /NCI-H460<br>1.09 /HeLa<br>1.00 /SK-OV-3<br>0.83 /BEL-7402<br>1.01 /HepG2<br>2.25 /HL-7702	—	—	—	—	staining with DCHF-DA<br><br>overproduction of intracellular ROS	BEL-7402 (Cu contents increased from 0.952 to 53.132 ± 6.120 µg per 10^6 cells)<br><br>Normal cellsHL-7702(Cu contents increased from 0.814 to 42.676 ± 5.280 µg per 10^6 cells)<br><br>The increase of Cu content in the mitochondria from 1.567 to 9.280 µg per 10^6 cells after treatment with complex<br><br>No obvious differences in the content of copper in the membrane fraction and nucleus fraction	Increasing of apoptotic cell from 3.41% (control group) to 55.8% after treatment with complex (1.6µM)<br><br>Caspase-3/9 activation	The deacrease of MMP of the BEL-7402 cells after treating with complex (0.8 µM)<br><br>In comparison with the control group (1.88%), the percentages of the decrease were 24.0%, 36.7%, and 60.0% for complex (0.4, 0.8 and 1.6 µM).	1
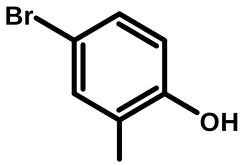<br>X = Cl	1.37 /MGC80-3<br>0.93 /T-24<br>1.18 /NCI-H460<br>1.15 /HeLa<br>1.00 /SK-OV-3<br>0.74 /Bel-7402<br>0.84 /HepG2<br>2.79 /HL-7702	—	—	—	—	staining with DCHF-DA<br><br>overproduction of intracellular ROS	—	The 60.6%of the apoptotic cells after treatment with complex (1.4 µM)	The same effect on the MMP of the BEL-7402 cells	1

Type of the R substituent and X atom	Western blot (protein expression)	Autophagy	Cell cycle progression	Cellular senescence	Cell migration	In vivo assays	Ref
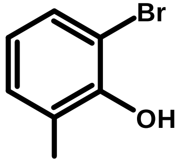 X = Cl	a) The expression levels of Chk1, Chk2, Cdc25A, Cyclin E and CDK2 inhibited and the expression levels of the p53 and p21 proteins increased with the addition of the complexes, when compared with the control group. The arrest of the BEL-7402cells in the G1 phase by inhibiting the activity of cyclinE-CDK2 and increasing the expression levels of p53 and p21, thus inhibiting cell proliferation. b) The levels of Apaf-1, Bax and cytochrome-c increased, the expression of Bcl-2 decreased. Induction of apoptosis via the mitochondria-mediated pathway	—	The percentage of cells in the G1 phase increased from48.92% to 71.25% after treatment with complex (0.8 μM).	—	—	Mouse xenograft model with human BEL-7402 liver cancer cells	1
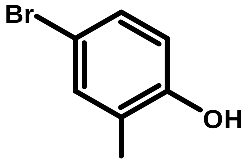 X = Cl	a) similar to that of complex 1 (Chk1, Chk2, Cdc25A, Cyclin E, p53, CDK2and p21 proteins) b) similar to that of complex 1 (Apaf-1, Bax, Bcl-2 and cytochrome-c)	—	The percentages of cells in the G1 phase increased from about 51.0% (control) to 75.23% after treatment with complex(1.4μM).	—	—	—	1

Type of the R substituent and X atom	IC50 (μM)/cell line) 2D	IC50 (μM)/cell line) 3D	Interaction with DNA	BSA/HSA binding	DNA clevage	ROS generation	Internalization	Apoptosis	Mitochondrial membrane potential	Western blot (protein expression)	Autophagy	Cell cycle progression	Cellular senescence	Cell migration	In vivo assays	Ref
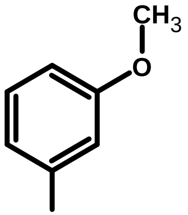<br>X = Cl	5.34/HeLa<br>2.72/HepG2<br>5.32/Bel-7402	—	UV-Vis titration ($K_b = 2.1×10^6$)<br><br>FID EB ($K_{app} = 4.5×10^6$)<br><br>CV	—	pDNA (pBR322), gel electrophoresis<br><br>ability of cleaving plasmid at about 30 μM	DCHF-DA assay<br><br>98.3% of ROS overexpressing cells	—	The microphotographs of AO/EB stained HeLa cells<br><br>Condensation of nuclei in the cells after24 h may suggest apoptosis	—	—	—	A significant increase in G0/G1 phase at 24 h. The raising of percentage of cells in G0/G1 phase from 47% in control to 61% after incubation for 24 h	—	—	—	2
	0.687/MCF-7<br>0.537/HT-29<br>0.236/CT-26<br>0.740/GM-5657	—	—	—	—	H2DCF-DA assay in HCT116 cells	Primarily accumulated in the cytoplasm of the cancer cells<br><br>Small amounts of complex in the nucleus, mitochondria, and the lysosomes	Enhanced survival of the cells after the incubation with Z-VAD-FMK; cell death mechanism primarily driven by apoptosis.<br><br>Cell death by apoptosis using the caspase 3/7 pathway	—	—	—	—	—	—	—	3

Type of the R substituent and X atom	IC50 (μM)/cell line) 2D	IC50 (μM)/cell line) 3D	Interaction with DNA	BSA/HSA binding	DNA clevage	ROS generation	Internalization	Apoptosis	Mitochondrial membrane potential	Western blot (protein expression)	Autophagy	Cell cycle progression	Cellular senescence	Cell migration	In vivo assays	Ref
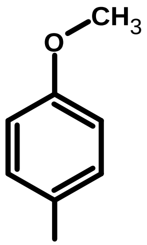<br>X = Cl	8.98/HeLa<br>4.13/HepG2<br>8.35/Bel-7402	—	UV-Vis titration (K_b = 5.8×10^6)<br><br>FID EB (K_{app} = 6.7×10^6)<br><br>CV	—	pDNA (pBR322), gel electrophoresis<br><br>ability of cleaving plasmid at about 30 μM	DCHF-DA assay<br><br>92.0% of ROS overexpressing cells	—	The microphotographs of AO/EB stained HeLa cells<br><br>Condensation of nuclei in the cells after24 h	—	—	—	A significant increase in G0/G1 phase at 24 h.<br><br>the raising of percentage of cells in G0/G1 phase from 47% in control to 65% after incubation for 24 h	—	—	—	2
	0.555/A549<br>0.507/Bel-7402<br>0.259/Eca-109<br>0.565/HeLa<br>0.578/MCF-7	—	CD<br>intercalation	—	—	—	—	—	—	—	—	—	—	—	—	4
	1.02 /HCT116<br>2.54 /A2780<br><0.3 /Fibroblasts	—	—	—	—	H2DCF-DA assay in HCT116 cells	—	—	—	—	—	—	—	—	—	5
	1.159 /MCF-7<br>3.620 /HT-29<br>1.247 /CT-26<br>2.761 /GM-5657	—	—	—	—	H2DCF-DA assay in HCT116 cells	—	—	—	—	—	—	—	—	—	3

Type of the R substituent and X atom	IC50 (µM)/cell line) 2D	IC50 (µM)/cell line) 3D	Interaction with DNA	BSA/HSA binding	DNA clevage	ROS generation	Internalization	Apoptosis	Mitochondrial membrane potential	Western blot (protein expression)	Autophagy	Cell cycle progression	Cellular senescence	Cell migration	In vivo assays	Ref
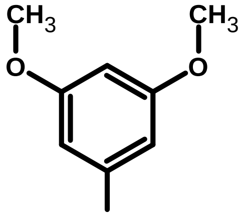<br><br>X = Cl	3.03/HeLa<br>3.91/HepG2<br>6.21/Bel-7402	—	UV-Vis titration<br>(K_b = 1.9×10^6)<br><br>FID EB<br>(K_{app} = 4.7×10^6)<br><br>CV	—	pDNA (pBR322), gel electrophoresis<br><br>ability of cleaving plasmid at about 30 µM	DCHF-DA assay<br><br>99.3% of ROS overexpressing cells	—	The microphotographs of AO/EB stained HeLa cells<br><br>Condensation of nuclei in the cells after24 h	—	—	—	A significant increase in G0/G1 phase at 24 h.<br><br>the raising of percentage of cells in G0/G1 phase from 47% in control to 56% after incubation for 24 h	—	—	—	2
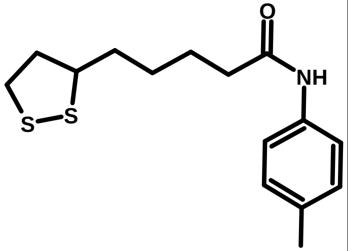<br><br>X = Cl	4.95/ MCF-7<br>3.25/ HeLa	—	FID EB<br>(K_{app} = 2.85×10^7)<br><br>CD	—	pDNA(pBR322) in the absence of oxidizing/reducing agents or any other additives.<br><br>DNA cleavage as low as ~12.5 µM	singlet oxygen scavengers (sodium azide and L-histidine) completely stopped the plasmid cleavage	—	Morphological changes in cell nucleus after 48 h incubation of 1 with HeLa and MCF-7 cells stained with Hoechst 33,258	—	—	—	For control, 85–90% of cell distributed atG1 phase of cell cycle,<br><br>Cells treated with complex distributed maximum at the sub-G1peak	—	—	—	6

Type of the R substituent and X atom	IC50 (μM)/cell line) 2D	IC50 (μM)/cell line) 3D	Interaction with DNA	BSA/HSA binding	DNA clevage	ROS generation	Internalization	Apoptosis	Mitochondrial membrane potential	Western blot (protein expression)	Autophagy	Cell cycle progression	Cellular senescence	Cell migration	In vivo assays	Ref
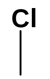<br>X = Cl	0.90/A2780<br>3.34/A2780cis<br>1.44/HeLa	—	electrostatic interaction/ external DNA-binding mode<br><br>FID EB<br>(K_{SV}= 0.90 ± 0.13×10^3)<br><br>UV-Vis titration<br>(K_b = 1.96 ± 0.64×10^6)	—	pBR322, agarose gel electrophoresis<br><br>Capable of efficiently cleaving DNA at a c = 20 μM, for which Form I is totally converted to mostly Form II and to some Form III	—	—	Assay with Annexin V-FITC<br><br>The rate of late apoptosis in HeLa cells treated with compound was 14.57%, a value close to that of cisplatin.<br>The ratio of early apoptotic cells decreased in treatment with the copper compound.	—	—	—	—	—	—	—	7
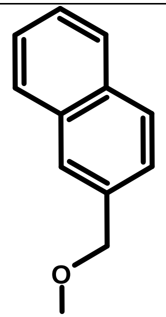<br>X = Cl	0.59/A2780<br>0.79/A2780cis<br>0.35/HeLa	—	electrostatic interaction/ external DNA-binding mode<br><br>FID EB<br>(K_{SV}= 5.96 ± 0.94×10^3)<br><br>UV-Vis titration<br>(K_b = 1.05 ± 0.02×10^6)	—	pBR322, agarose gel electrophoresis<br><br>Some alteration of the DNA structure/conformation is only seen from c > 80 μM	—	—	Assay with Annexin V-FITC<br><br>The rate of late apoptosis in HeLa cells treated with compound was 21.5%.<br>The ratio of early apoptotic cells decreased in treatment with the copper compound.	—	—	—	—	—	—	—	7

Type of the R substituent and X atom	IC50 (μM)/cell line) 2D	IC50 (μM)/cell line) 3D	Interaction with DNA	BSA/HSA binding	DNA clevage	ROS generation	Internalization	Apoptosis	Mitochondrial membrane potential	Western blot (protein expression)	Autophagy	Cell cycle progression	Cellular senescence	Cell migration	In vivo assays	Ref
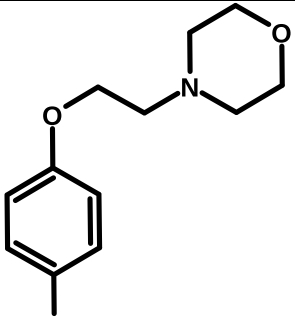<br>X = Cl	1.62/HL-60<br>3.59/BGC-823<br>2.28/KB<br>0.63/Bel-7402	—	—	—	—	—	—	—	—	—	—	—	—	—	—	8
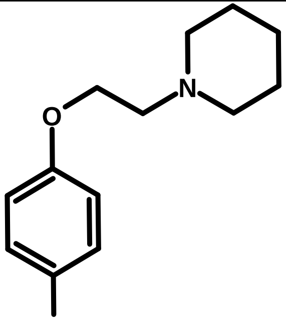<br>X = Cl	3.40/HL-60<br>3.73/BGC-823<br>2.37/KB<br>1.09/Bel-7402	—	partial intercalation through the planar terpyridine moiety and groove binding through the piperidine or copper(II)-bis(2-pyridylmethyl)amine side arms (either by electrostatic or covalent interactions)<br><br>UV-Vis titration<br>(K_b = 1.35±0.11×10^4)<br><br>FID TO and Hoechst 33258	—	supercoiled pUC19 DNA<br>Low cleaving properties<br><br>13% cleavage of supercoiled pUC19 DNA with c = 100 μM	—	—	—	—	—	—	—	—	—	—	8,9

Type of the R substituent and X atom	IC50 (µM)/cell line) 2D	IC50 (µM)/cell line) 3D	Interaction with DNA	BSA/HSA binding	DNA cleavage	ROS generation	Internalization	Apoptosis	Mitochondrial membrane potential	western blot (protein expression)	Autophagy	Cell cycle progression	Cellular senescence	Cell migration	In vivo assays	Ref
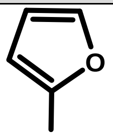<br>X = Cl	17.3 /HCT116<br>2.9 /A2780<br>20.5 /A549<br>43 / MCF-7<br>59.5 /Fibroblasts	—	—	—	—	—	—	Hoechst 33258 staining of A2780 cells after 48 h of exposure to the IC_{50} of complexes<br><br>Reduction in the number of stained cells compared to control cells and the nuclear condensation and fragmentation characteristic for apoptosis	—	—	—	—	—	—	—	10
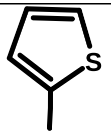<br>X = Cl	2.1 /HCT116<br>> 100/A2780<br>4.2 /A549<br>> 100/ MCF-7<br>> 100/Fibroblasts	—	—	—	—	—	—		—	—	—	—	—	—	—	10
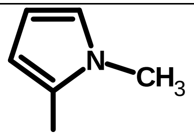<br>X = Cl	> 100/HCT116<br>> 100/A2780<br>> 100/A549<br>> 100/ MCF-7<br>> 100/Fibroblasts	—	—	—	—	—	—		—	—	—	—	—	—	—	10
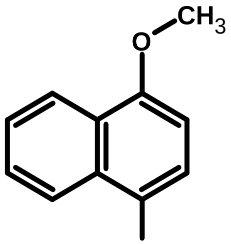<br>X = Cl	0.40 /HCT116<br>0.95 /A2780<br>32.78 /Fibroblasts	—	—	—	—	H2DCF-DA assay in HCT116 cells	—	Hoechst 33258 staining of HCT116 cells after 48 h of exposure to the IC_{50} of complex<br><br>Reduction in the number of stained cells compared to control cells and the nuclear condensation and fragmentation characteristic of apoptosis<br><br>Annexin V-FITC/PI assay<br><br>High levels of apoptosis even when compared to doxorubicin	—	—	—	—	—	—	—	5

Type of the R substituent and X atom	IC50 (µM)/cell line) 2D	IC50 (µM)/cell line) 3D	Interaction with DNA	BSA/HSA binding	DNA cleavage	Internalization	Apoptosis	Mitochondrial membrane potential	western blot (protein expression)	Autophagy	Cell cycle progression	Cellular senescence	Cell migration	In vivo assays	Ref
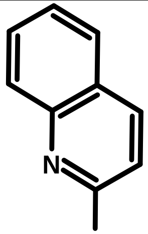<br>X = Cl	0.3 ± 0.09/HCT116<br>0.5 ± 0.08/A2780<br>9.4 ± 0.02/Fibroblasts	—	FID EB intercalation<br>(K_{app} = 1.10 ×10^6)	—	—	—	—	—	—	—	—	—	—	—	11
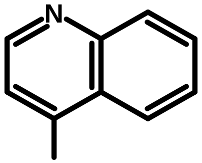<br>X = Cl	0.3 ± 0.06/HCT116<br>0.5 ± 0.09/A2780<br>> 50/Fibroblasts	—	FID EB intercalation<br>(K_{app} = 8.68 ×10^6)	—	—	—	Increase of cells in apoptosis compared to the control.<br>Apoptotic figures increase ~124% in cells treated with IC_{50} concentration.	—	—	—	—	—	—	—	11

Type of the R substituent and X atom	IC50 (µM)/cell line) 2D	IC50 (µM)/cell line) 3D	Interaction with DNA	BSA/HSA binding	DNA cleavage	ROS generation	Internalization	Apoptosis	Mitochondrial membrane potential	western blot (protein expression)	Autophagy	Cell cycle progression	Cellular senescence	Cell migration	In vivo assays	Ref
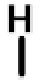<br>X = Cl	3.2 /MCF-7<br>2.7 /HT-29<br>2.3 /CT-26<br>3.4 /GM-5657	—	—	—	—	H2DCF-DA assay in HCT116 cells	—	—	—	—	—	—	—	—	—	3
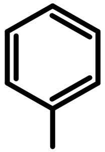	1.1 /MCF-7<br>1.5 /HT-29<br>1.3 /CT-26<br>2.1/GM-5657	—	—	—	—	H2DCF-DA assay in HCT116 cells	—	—	—	—	—	—	—	—	—	3
X = Cl	0.858/A549<br>0.661/Bel-7402<br>0.279/Eca-109<br>0.760/HeLa<br>1.355/MCF-7	—	intercalation	—	—	—	—	—	—	—	—	—	—	—	—	4
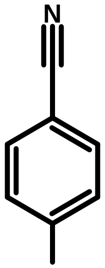<br>X = Cl	0.343/A549<br>1.525/Bel-7402<br>0.775/Eca-109<br>1.055/HeLa<br>1.355/MCF-7	—	intercalation	—	—	—	—	—	—	—	—	—	—	—	—	4

Type of the R substituent and X atom	IC50 (μM)/cell line) 2D	IC50 (μM)/cell line) 3D	Interaction with DNA	BSA/HSA binding	DNA cleavage	ROS generation	Internalization	Apoptosis	Mitochondrial membrane potential	western blot (protein expression)	Autophagy	Cell cycle progression	Cellular senescence	Cell migration	In vivo assays	Ref
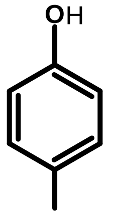<br>X = Cl	0.177/A549<br>1.924/Bel-7402<br>0.352/Eca-109<br>0.788/HeLa<br>0.996/MCF-7	—	intercalation	—	—	—	—	—	—	—	—	—	—	—	—	4
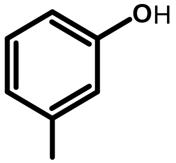<br>X = Cl	0.271/A549<br>1.782/Bel-7402<br>0.433/Eca-109<br>0.437/HeLa<br>0.351/MCF-7	—	intercalation	—	—	—	—	—	—	—	—	—	—	—	—	4
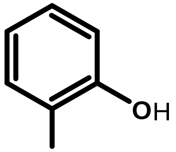<br>X = Cl	0.231/A549<br>1.271/Bel-7402<br>0.584/Eca-109<br>1.078/HeLa<br>0.374/MCF-7	—	intercalation	—	—	—	—	—	—	—	—	—	—	—	—	4

Type of the R substituent and X atom	IC50 (μM)/cell line) 2D	IC50 (μM)/cell line) 3D	Interaction with DNA	BSA/HSA binding	DNA cleavage	ROS generation	Internalization	Apoptosis	Mitochondrial membrane potential	western blot (protein expression)	Autophagy	Cell cycle progression	Cellular senescence	Cell migration	In vivo assays	Ref
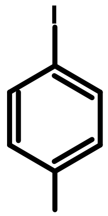 X = Cl	0.839/A549<br>0.860/Bel-7402<br>1.049/Eca-109<br>1.454/HeLa<br>0.276/MCF-7	—	intercalation	—	—	—	—	—	—	—	—	—	—	—	—	4
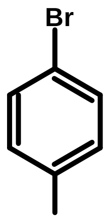 X = Cl	1.165/A549<br>0.585/Bel-7402<br>0.777/Eca-109<br>1.347/HeLa<br>0.216/MCF-7	—	intercalation	—	—	—	—	—	—	—	—	—	—	—	—	4
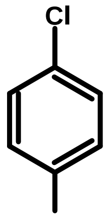 X = Cl	0.658/A549<br>0.611/Bel-7402<br>1.015/Eca-109<br>1.438/HeLa<br>0.738/MCF-7	—	intercalation	—	—	—	—	—	—	—	—	—	—	—	—	4

Type of the R substituent and X atom	IC50 (μM)/cell line) 2D	IC50 (μM)/cell line) 3D	Interaction with DNA	BSA/HSA binding	DNA cleavage	ROS generation	Internalization	Apoptosis	Mitochondrial membrane potential	western blot (protein expression)	Autophagy	Cell cycle progression	Cellular senescence	Cell migration	In vivo assays	Ref
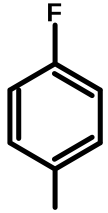 X = Cl	1.155/A549<br>0.571/Bel-7402<br>0.777/Eca-109<br>0.912/HeLa<br>0.255/MCF-7	—	intercalation	—	—	—	—	—	—	—	—	—	—	—	—	4
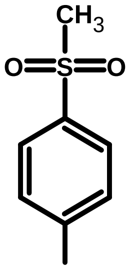 X = Cl	0.533/A549<br>0.727/Bel-7402<br>0.312/Eca-109<br>1.425/HeLa<br>1.365/MCF-7	—	intercalation	—	—	—	—	—	—	—	—	—	—	—	—	4
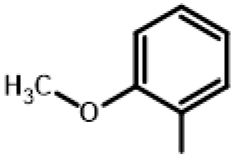 X = Cl	0.542 /MCF-7<br>0.964 /HT-29<br>0.630 /CT-26<br>0.841 /GM-5657	—	—	—	—	H2DCF-DA assay in HCT116 cells	—	—	—	—	—	—	—	—	—	3

Type of the R substituent and X atom	IC50 (μM)/cell line) 2D	IC50 (μM)/cell line) 3D	Interaction with DNA	BSA/HSA binding	DNA cleavage	ROS generation	Internalization	Ref
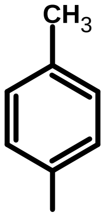 X = Br	1.9 /MDA-MB-231<br>2.1 /A549<br>2.0 /HeLa	—	UV-Vis titration<br>(K_b = 6.36×10^5)<br>hypochromism<br><br>FID EB<br>(K_{app} = 1.25×10^{12})<br><br>Viscosity<br>viscosity of DNA increased<br><br>intercalation	BSA binding<br>fluorescence quenching<br>(K_{bin} = 5.25×10^4)<br>moderate binding to BSA	pDNA (pBR322), gel electrophoresis<br><br>DNA cleavage at c = 35 μM	H2DCF-DA assay	—	12

Type of the R substituent and X atom	Apoptosis	Mitochondrial membrane potential	Western blot (protein expression)	Autophagy	Cell cycle progression	Cellular senescence	Cell migration	In vivo assays	Ref
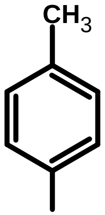 X = Br	HE staining<br>Hoechst 33342 staining<br>morphological changes and nuclear chromatin condensation<br><br>Annexin V-FITC/PI staining,<br>10.52% apoptosis rate<br><br>Caspase 3 activation	—	—	—	The population of cells in S and G2/M phases declined, while G0/G1 phase increased<br><br>Cell cycle arrest<br>at the G0/G1 phase	—	—	—	12

Type of he R substituent and X atom	IC50 (µM)/cell line) 2D	IC50 (µM)/cell line) 3D	Interaction with DNA	BSA/HSA binding	DNA cleavage	ROS generation	Internalization	Apoptosis	Ref
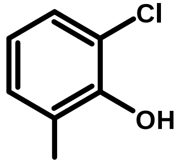 X = Br	5.3 /HL-7702<br>3.9 /NCI-H460<br>4.7 /T-24<br>5.2 /SK-OV-3	—	—	—	—	level of ROS in T-24 cells was higher when treated with complexes.	increase of the content of Cu in T-24 cells, highest increase (~27x) in mitochondria	Annexin V/PI staining Increase of apoptotic cells from 4.94% to 8.73% after 48h incubation with of 3 µM of complex<br><br>release of Ca^{2+} ions<br><br>Caspase-3/9 activation	13
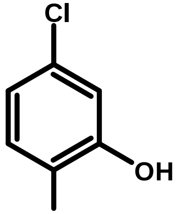 X = Br	5.2 /HL-7702<br>2.5 /NCI-H460<br>1.9 /T-24<br>2.2 /SK-OV-3	—	—	—	—	level of ROS in T-24 cells was higher when treated with complexes.	increase of the content of Cu in T-24 cells, highest increase (~39x) in mitochondria	Annexin V/PI staining Increase of apoptotic cells from 4.94% to 25.8% after 48h incubation with of 3 µM of complex<br><br>release of Ca^{2+} ions<br><br>Caspase-3/9 activation	13
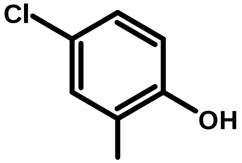 X = Br	5.7 /HL-7702<br>3.3 /NCI-H460<br>3.0 /T-24<br>2.7 /SK-OV-3	—	—	—	—	level of ROS in T-24 cells was higher when treated with complexes.	increase of the content of Cu in T-24 cells, highest increase (~30x) in mitochondria	Annexin V/PI staining Increase of apoptotic cells from 4.94% to 13.05% after 48h incubation with of 3 µM of complex<br><br>release of Ca^{2+} ions<br><br>Caspase-3/9 activation	13

Type of the R substituent and X atom	Mitochondrial membrane potential	Western blot (protein expression)	Autophagy	Cell cycle progression	Cellular senescence	Cell migration	In vivo assays	Ref
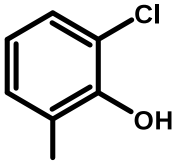 X = Br	decrease of MMP17.8% after 48h incubation with of 3 μM of complex	expression levels of Bcl-2, Chk1,Chk2, Cdc25A, Cyclin D, CDK2, CDK4 and CDK6 decreased; p53 and Bax increased	—	The percentage of cells in the G1 phase increased from 36.34% to 65.23% after treatment with complex (3μM).<br><br>Cell cycle arrest in the G1 phase	—	—	mouse xenograft model inoculated with T-24 cells<br><br>no significant toxicity up to 30mg/kg/day<br><br>inhibition of tumor growth	13
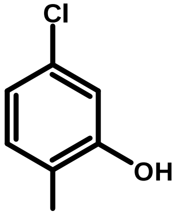 X = Br	decrease of MMPby 40.6% after 48h incubation with of 3 μM of complex	expression levels of Bcl-2, Chk1,Chk2, Cdc25A, Cyclin D, CDK2, CDK4 and CDK6 decreased; p53 and Bax increased	—	The percentage of cells in the G1 phase increased from 36.34% to 75.46% after treatment with complex (3μM).<br><br>Cell cycle arrest in the G1 phase	—	—	mouse xenograft model inoculated with T-24 cells<br><br>no significant toxicity<br><br>inhibition of tumor growth	13
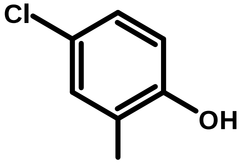 X = Br	decrease of MMPby 23.8% after 48h incubation with of 3 μM of complex	expression levels of Bcl-2, Chk1,Chk2, Cdc25A, Cyclin D, CDK2, CDK4 and CDK6 decreased; p53 and Bax increased	—	The percentage of cells in the G1 phase increased from 36.34% to 67.51% after treatment with complex (3μM).<br><br>Cell cycle arrest in the G1 phase	—	—	mouse xenograft model inoculated with T-24 cells<br><br>no significant toxicity<br><br>inhibition of tumor growth	13

Type of the R substituent and X atom	IC50 (μM)/cell line) 2D	IC50 (μM)/cell line) 3D	Interaction with DNA	BSA/HSA binding	DNA cleavage	ROS generation	Internalization	Apoptosis	Ref
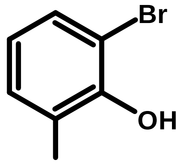<br>X = Br	4.6 /NCI-H460<br>3.2 /T-24<br>2.4 /SK-OV-3<br>15.9 /HL-7702	–	–	–	–	level of ROS in T-24 cells was higher when treated with complexes.	increase of the content of Cu in T-24 cells, from 9.16 ± 0.60 μg to 39.6 ± 3.80 μg/10^6 cells at the concentration of 2.9 μM, highest increase in mitochondria	Annexin V/PI staining<br>Increase of apoptotic cells from 5.22% to 14.96% after 48h incubation with of 2.9μM of complex<br><br>release of Ca^{2+} ions<br><br>Caspase-3/9 activation	14
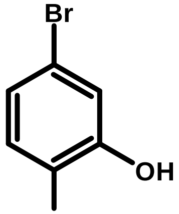<br>X = Br	4.4 /NCI-H460<br>3.3 /T-24<br>4.0 /SK-OV-3<br>14.7 /HL-7702	–	–	–	–	level of ROS in T-24 cells was higher when treated with complexes.	increase of the content of Cu in T-24 cells, highest increase in mitochondria	Annexin V/PI staining<br>Increase of apoptotic cells from 5.22% to 12.65% after 48h incubation with of 2.9μM of complex<br><br>release of Ca^{2+} ions<br><br>Caspase-3/9 activation	14
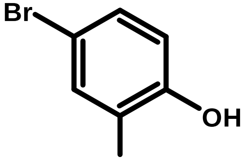<br>X = Br	3.2 /NCI-H460<br>2.9 /T-24<br>3.4 /SK-OV-3<br>13.7 /HL-7702	–	–	–	–	level of ROS in T-24 cells was higher when treated with complexes.	increase of the content of Cu in T-24 cells, from 9.16 ± 0.60 μg to 41.12 ± 3.60 μg/10^6 cells at the concentration of 2.9 μM, highest increase in mitochondria	Annexin V/PI staining<br>Increase of apoptotic cells from 5.22% to 20.87% after 48h incubation with of 2.9μM of complex<br><br>release of Ca^{2+} ions<br><br>Caspase-3/9 activation	14

Type of the R substituent and X atom	Mitochondrial membrane potential	Western blot (protein expression)	Autophagy	Cell cycle progression	Cellular senescence	Cell migration	In vivo assays	Ref
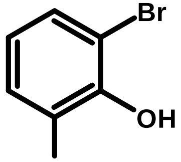 X = Br	mitochondrial membrane of the T-24cells was affected by complex, resulting in increased permeability<br><br>decrease of MMP	expression levels of Bcl-2, CHK2, CDK4, CDK6 and Cyclin D decreased; p53 and Bax increased	–	The percentage of T-24 cells in the G1 phase increased from40.83% to 59.74% after treatment with complex (2.9µM).<br><br>Cell cycle arrest in the G1 phase	–	–	–	14
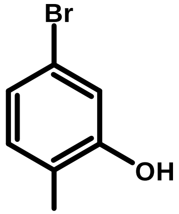 X = Br	mitochondrial membrane of the T-24cells was affected by complex, resulting in increased permeability<br><br>decrease of MMP	expression levels of Bcl-2,CHK2, CDK4, CDK6 and Cyclin D decreased; p53 and Bax increased	–	The percentage of T-24 cells in the G1 phase increased from40.83% to 53.66% after treatment with complex (2.9µM).<br><br>Cell cycle arrest in the G1 phase	–	–	–	14
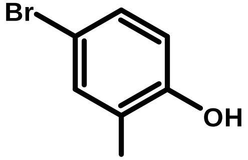 X = Br	mitochondrial membrane of the T-24cells was affected by complex, resulting in increased permeability<br><br>decrease of MMP	expression levels of Bcl-2, CHK2, CDK4, CDK6 and Cyclin D decreased; p53 and Bax increased	–	The percentage of T-24 cells in the G1 phase increased from40.83% to 65.24% after treatment with complex (2.9µM).<br><br>Cell cycle arrest in the G1 phase	–	–	mouse xenograft model inoculated with T-24 cells<br><br>no significant toxicity up to 30mg/kg/day<br><br>tumor-suppressive effect, slightly less effective than that of cisplatin	14

2. General Characterization

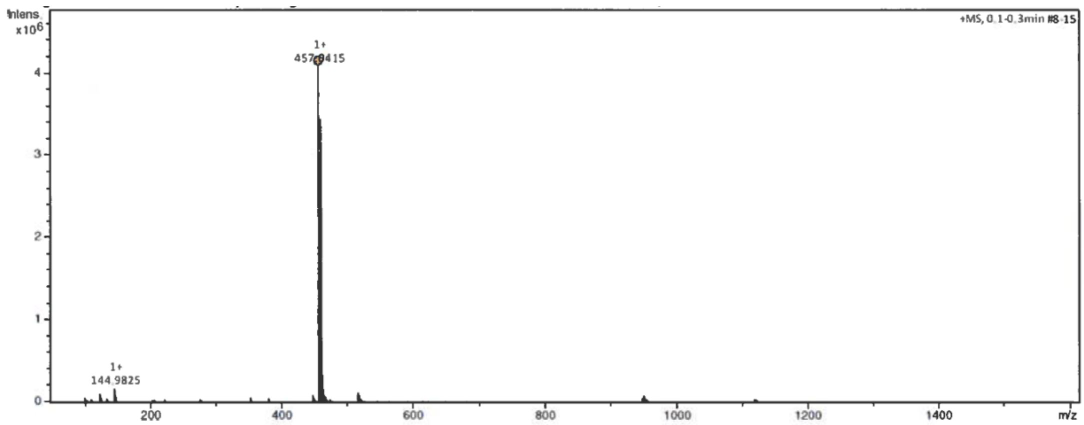

Meas. m/z	#	Ion Formula	m/z	err [ppm]	mSigma	# Sigma	Score	rdb	e— Conf	N-Rule
457.0415	1	**C25H17ClCuN3**	457.0401	**3.0**	26.9	1	100.00	18.0	odd	ok

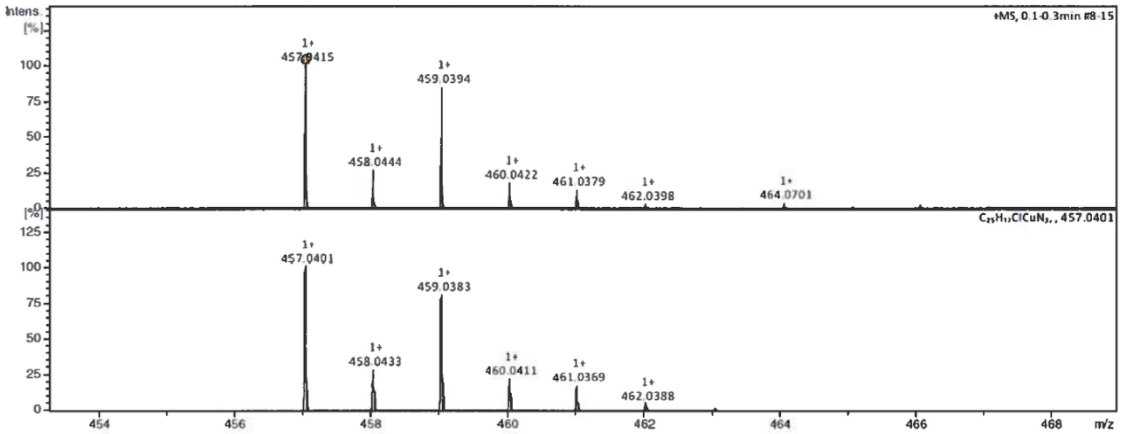

Cu1a

HR-MS (ESI): calcd for $C_{25}H_{17}ClCuN_3^+$ $[M-Cl]^+$ 457.0401 found 457.0415.

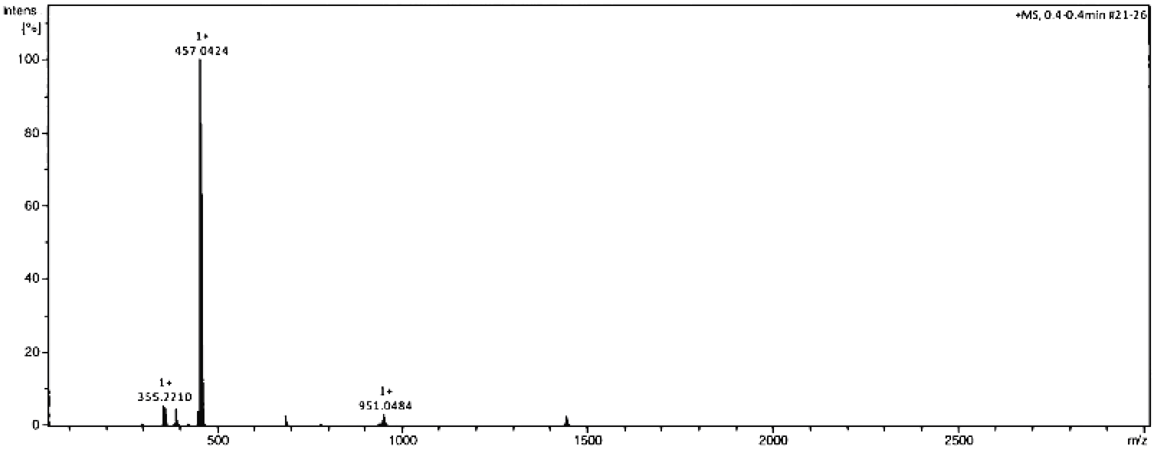

Meas. m/z	#	Ion Formula	m/z	err [ppm]	mSigma	# Sigma	Score	rdb	e— Conf	N-Rule
457.0424	1	C25H17ClCuN3	457.0401	5.0	13.3	1	100.00	18.0	odd	ok

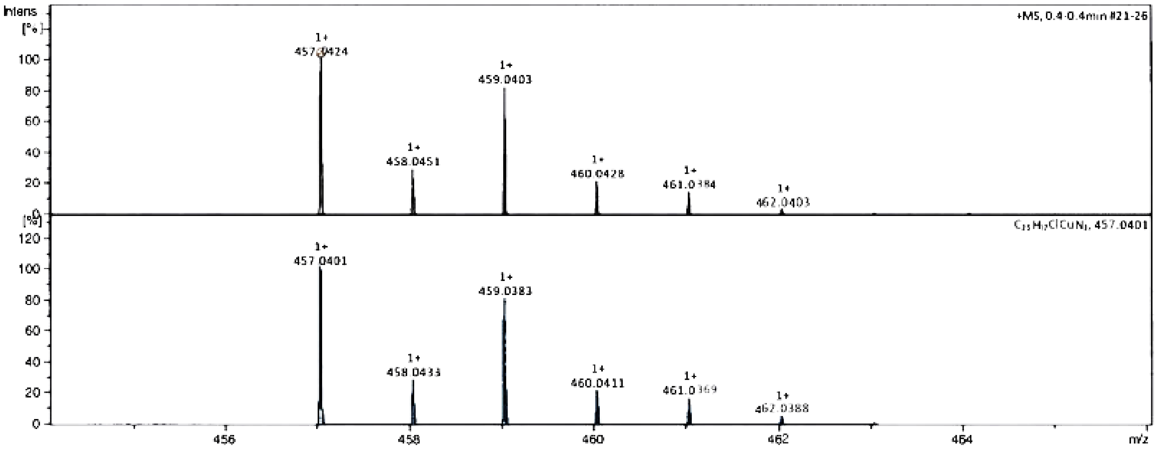

Cu1b

HR-MS (ESI): calcd for $C_{25}H_{17}ClCuN_3^+$ [M-Cl]$^+$ 457.0401 found 457.0424.

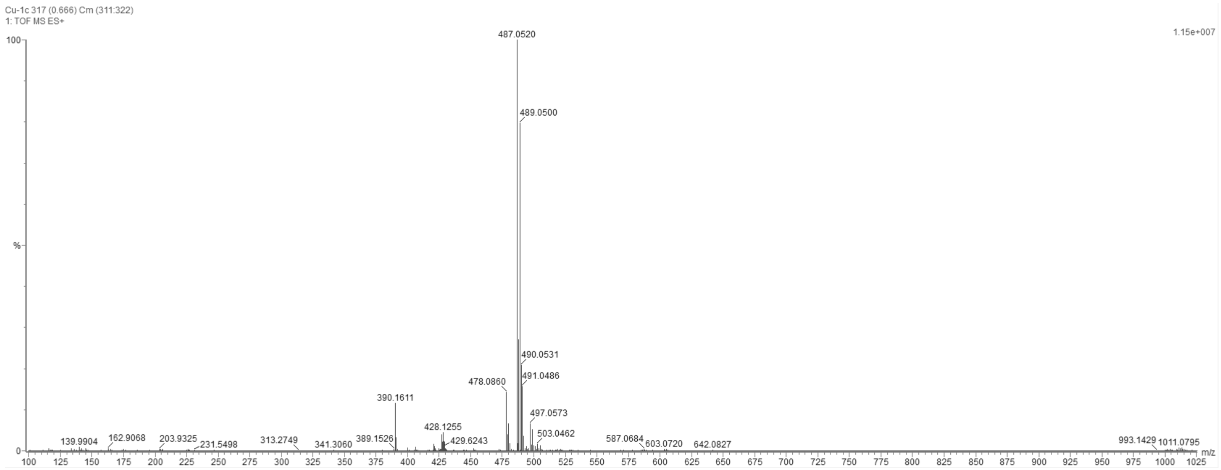

Mass	RA	Calc. Mass	mDa	PPM	DBE	Formula	i-FIT	i-FIT Norm	Fit Conf %	C	H	N	O	Cl	Cu
487.0520	100.00	487.0513	0.7	1.4	18.5	C26 H19 N3 O Cl Cu	559.1	n/a	n/a	26	19	3	1	1	1
489.0500	79.74	489.0436	6.4	13.1	13.5	C23 H22 N3 O Cl2 Cu	536.5	n/a	n/a	23	22	3	1	2	1

Cu1c

HR-MS (ESI): calcd for $C_{26}H_{19}ClCuN_3O^+$ $[M-Cl]^+$ 487.0520 found 487.0513.

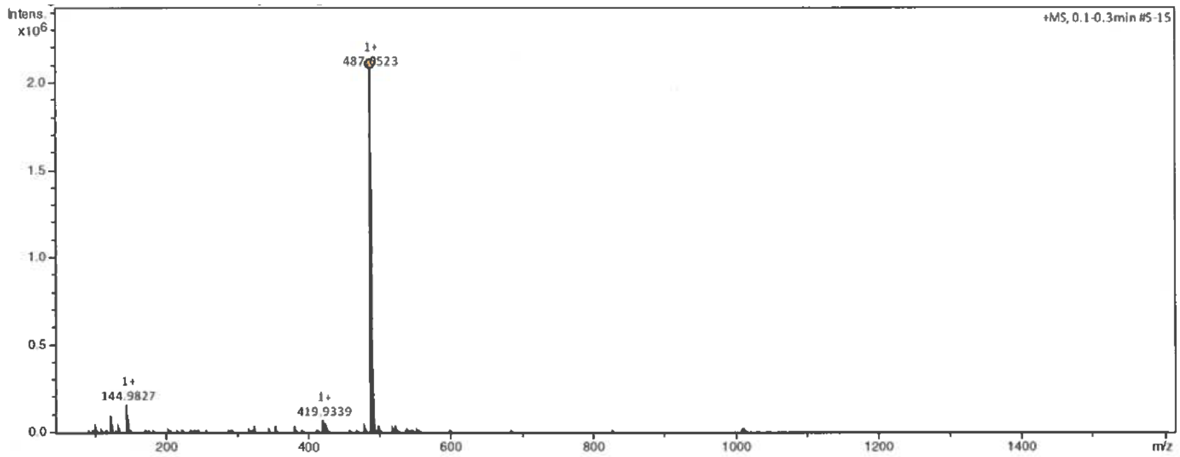

Meas. m/z	#	Ion Formula	m/z	err [ppm]	mSigma	# Sigma	Score	rdb	e— Conf	N-Rule
487.0523	1	**C26H19ClCuN3O**	487.0507	**3.3**	41.1	1	100.00	18.0	odd	ok

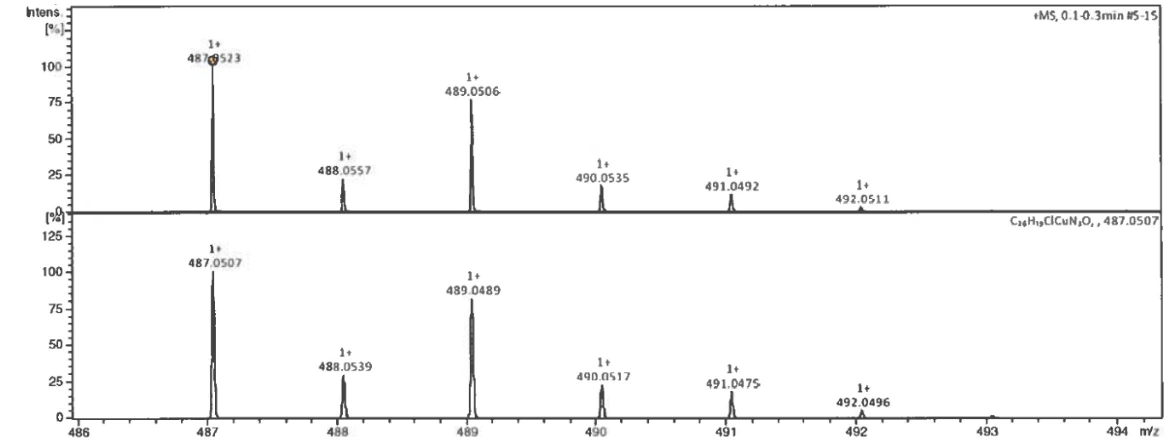

Cu1d

HR-MS (ESI): calcd for $C_{26}H_{19}ClCuN_3O^+$ $[M-Cl]^+$ 487.0507 found 487.0523.

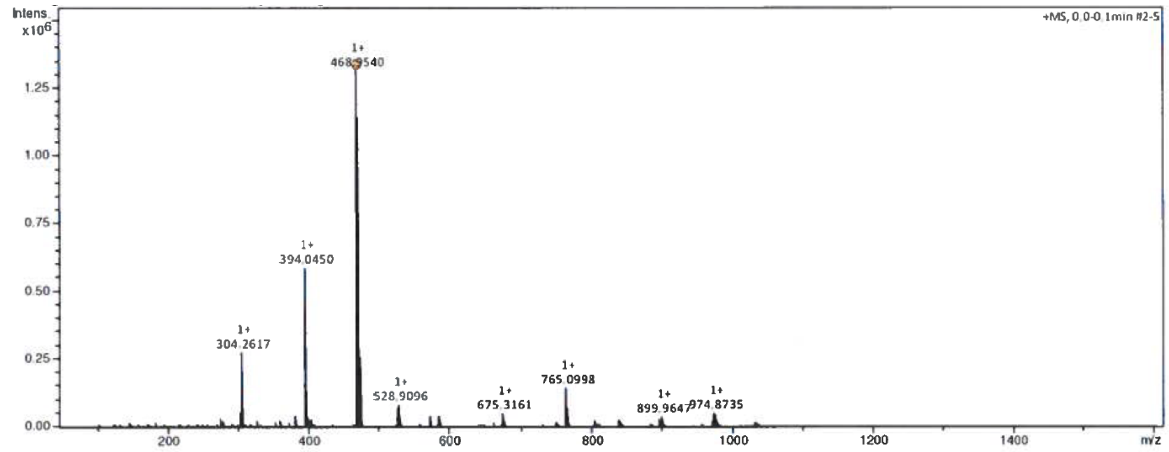

Meas. m/z	#	Ion Formula	m/z	err [ppm]	mSigma	# Sigma	Score	rdb	e— Conf	N-Rule
468.9540	1	**C21H13ClCuN3S2**	468.9530	**2.1**	24.4	1	100.00	16.0	odd	ok

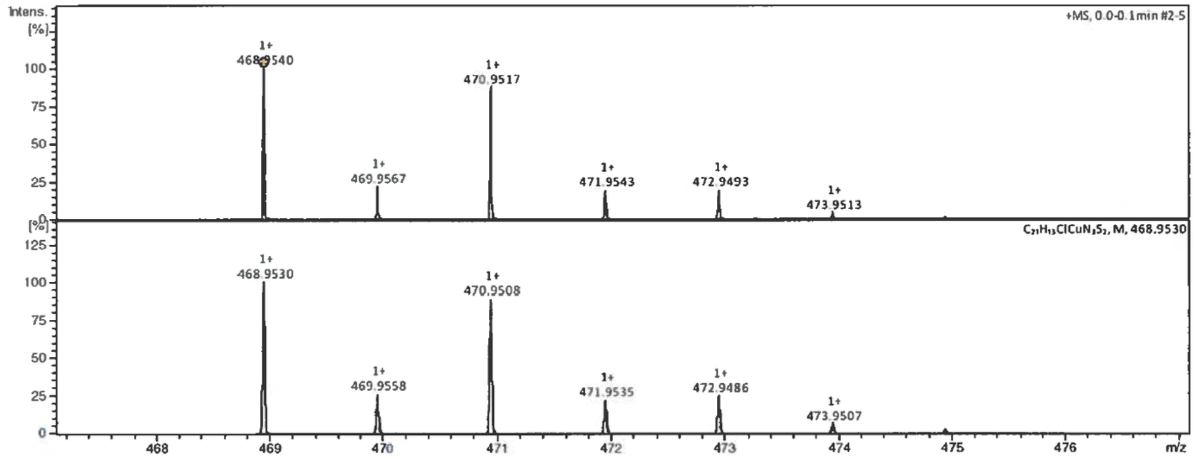

Cu2a

HR-MS (ESI): calcd for $C_{21}H_{13}ClCuN_3S_2^+$ [M-Cl]$^+$ 468.9530 found 468.9540.

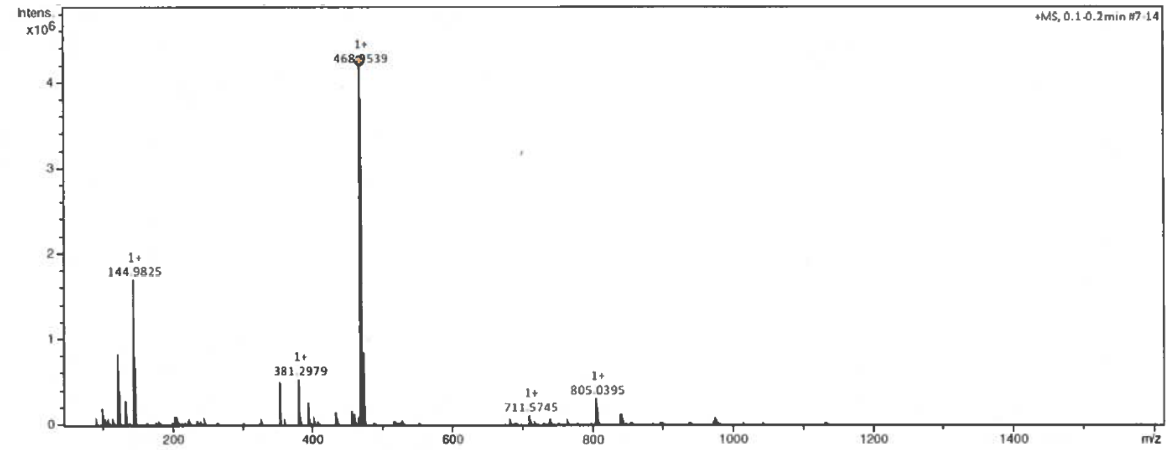

Meas. m/z	#	Ion Formula	m/z	err [ppm]	mSigma	# Sigma	Score	rdb	e— Conf	N-Rule
468.9539	1	**C21H13ClCuN3S2**	468.9530	**1.9**	22.4	1	100.00	16.0	odd	ok

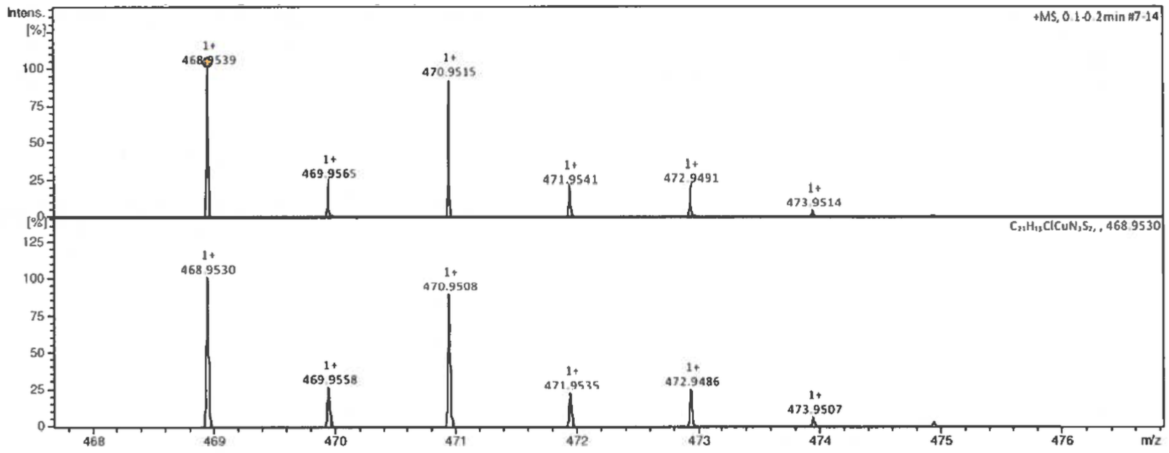

Cu2b

HR-MS (ESI): calcd for $C_{21}H_{13}ClCuN_3S_2^+$ $[M-Cl]^+$ 468.9530 found 468.9539.

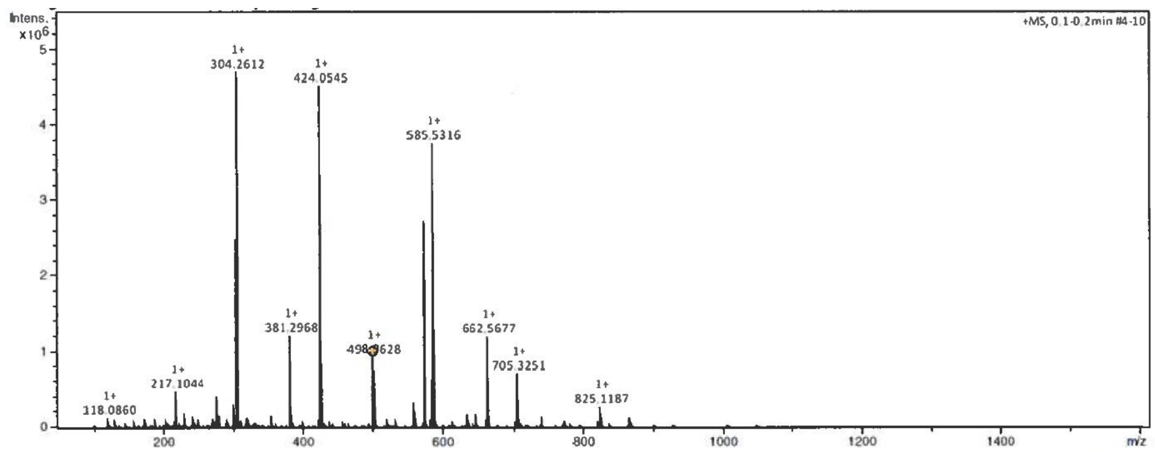

Meas. m/z	#	Ion Formula	m/z	err [ppm]	mSigma	# Sigma	Score	rdb	e— Conf	N-Rule
498.9628	1	**C22H15ClCuN3OS2**	498.9636	**-1.5**	53.1	1	100.00	16.0	odd	ok

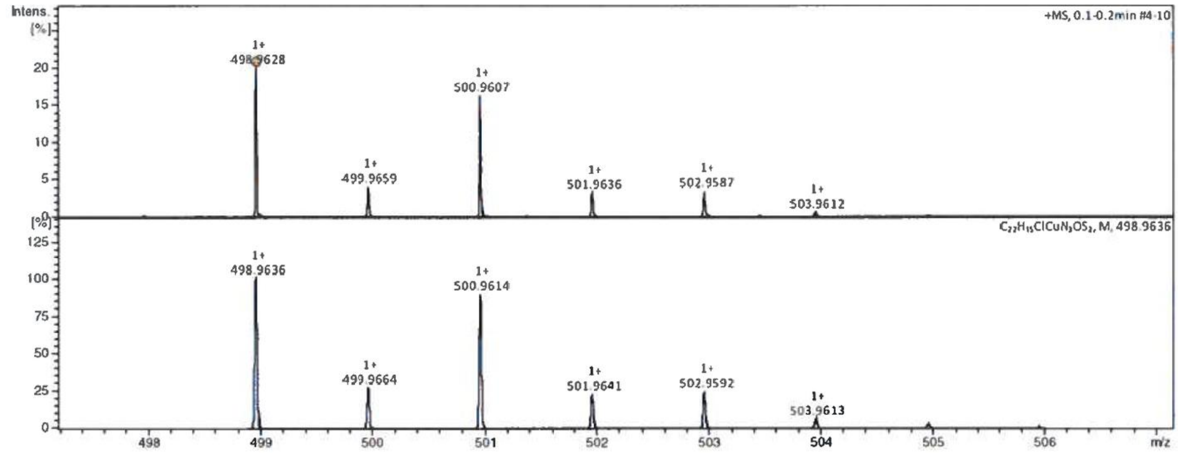

Cu2c

HR-MS (ESI): calcd for $C_{22}H_{15}ClCuN_3OS_2^+$ $[M-Cl]^+$ 498.9636 found 498.9628.

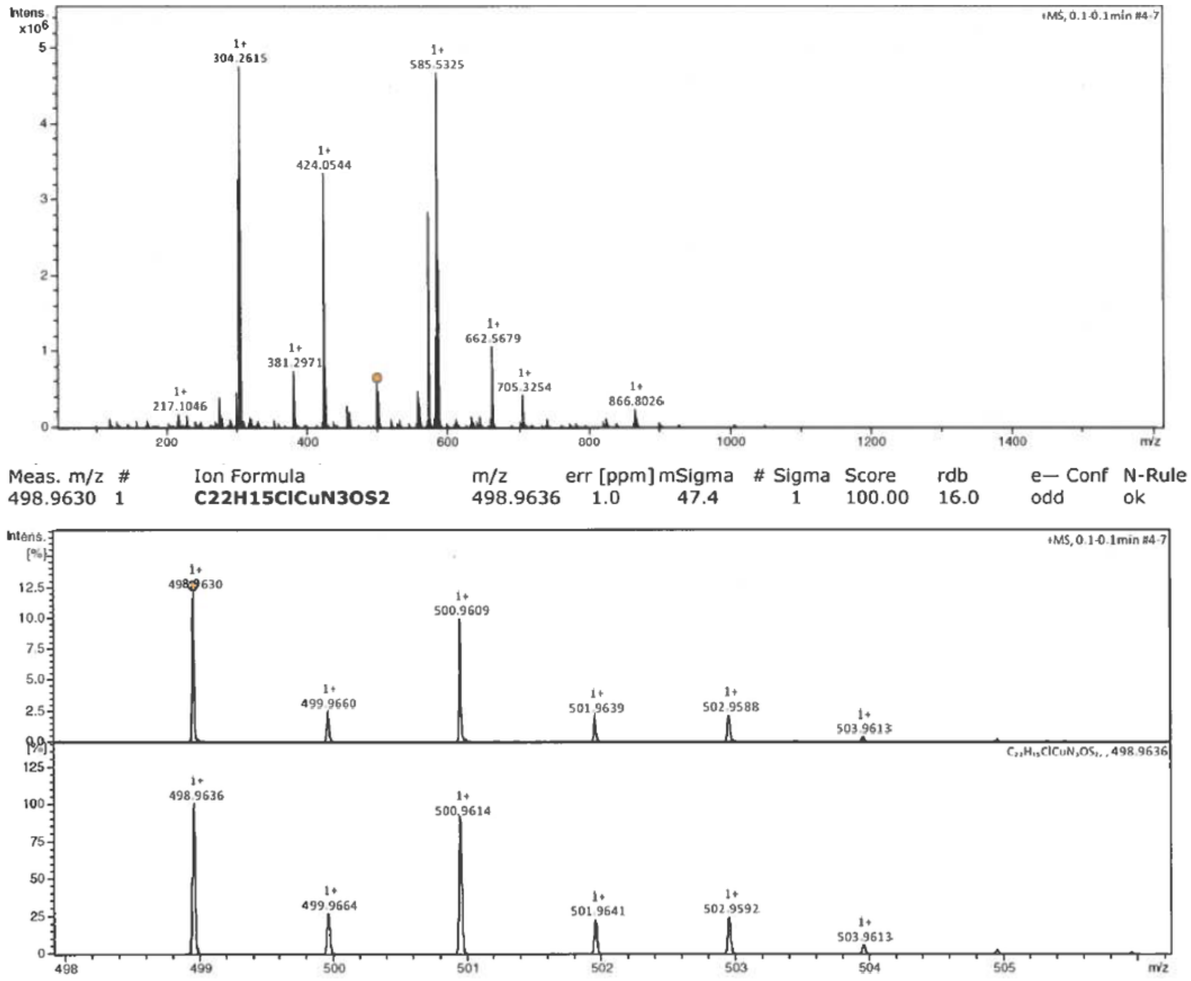

Cu2d

HR-MS (ESI): calcd for $C_{22}H_{15}ClCuN_3OS_2^+$ $[M-Cl]^+$ 498.9636 found 498.9630.

Figure S1. HR-MS of copper(II) complexes.

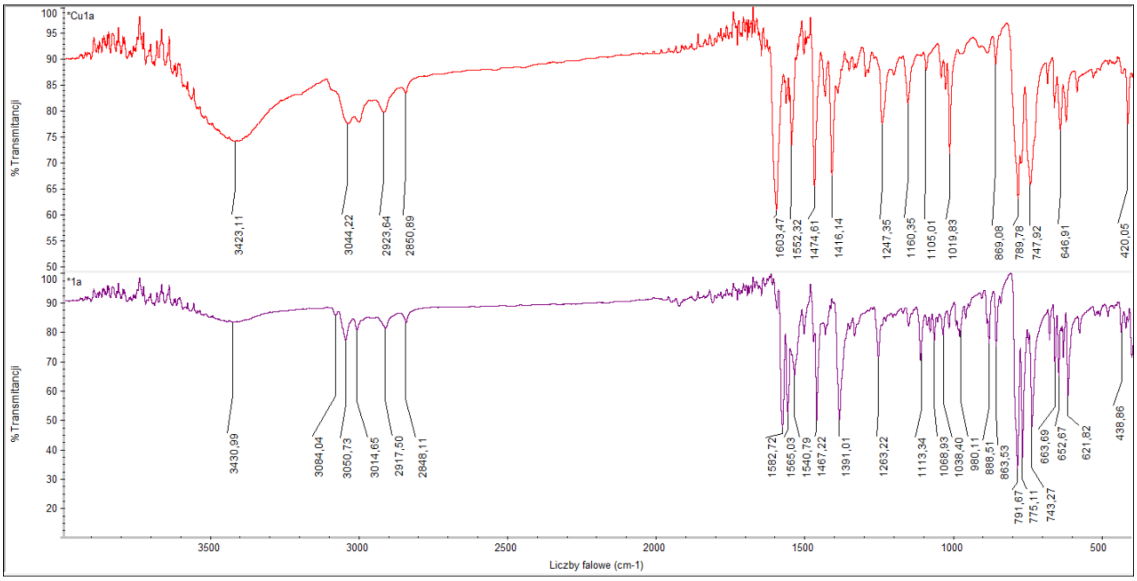
*Cu1a
*1a
% Transmitancji
% Transmitancji
3423,11
3044,22
2923,64
2850,89
1603,47
1552,32
1474,61
1416,14
1247,35
1160,35
1105,01
1019,83
869,08
789,78
747,92
646,91
420,05
3430,99
3084,04
3050,73
3014,65
2917,50
2848,11
1582,72
1565,03
1540,79
1467,22
1391,01
1263,22
1113,34
1068,93
1038,40
980,11
888,51
863,53
791,67
775,11
743,27
663,69
652,67
621,82
438,86
Liczby falowe (cm-1)

Cu1a

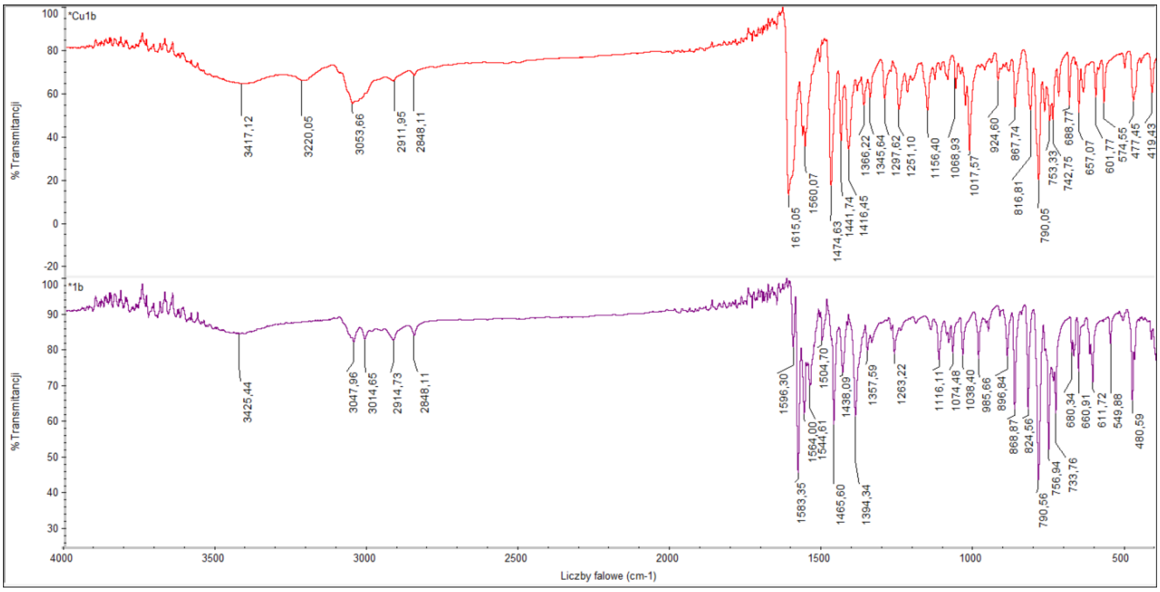
*Cu1b
*1b
% Transmitancji
% Transmitancji
3417,12
3220,05
3053,66
2911,95
2848,11
1615,05
1560,07
1474,63
1441,74
1416,45
1366,22
1345,64
1297,62
1251,10
1156,40
1068,93
1017,57
924,60
867,74
816,81
790,05
753,33
742,75
688,77
657,07
601,77
574,55
477,45
419,43
3425,44
3047,96
3014,65
2914,73
2848,11
1596,30
1583,35
1564,00
1544,61
1504,70
1465,60
1438,09
1394,34
1357,59
1263,22
1116,11
1074,48
1038,40
985,66
896,84
868,87
824,56
790,56
756,94
733,76
680,34
660,91
611,72
549,88
480,59
Liczby falowe (cm-1)

Cu1b

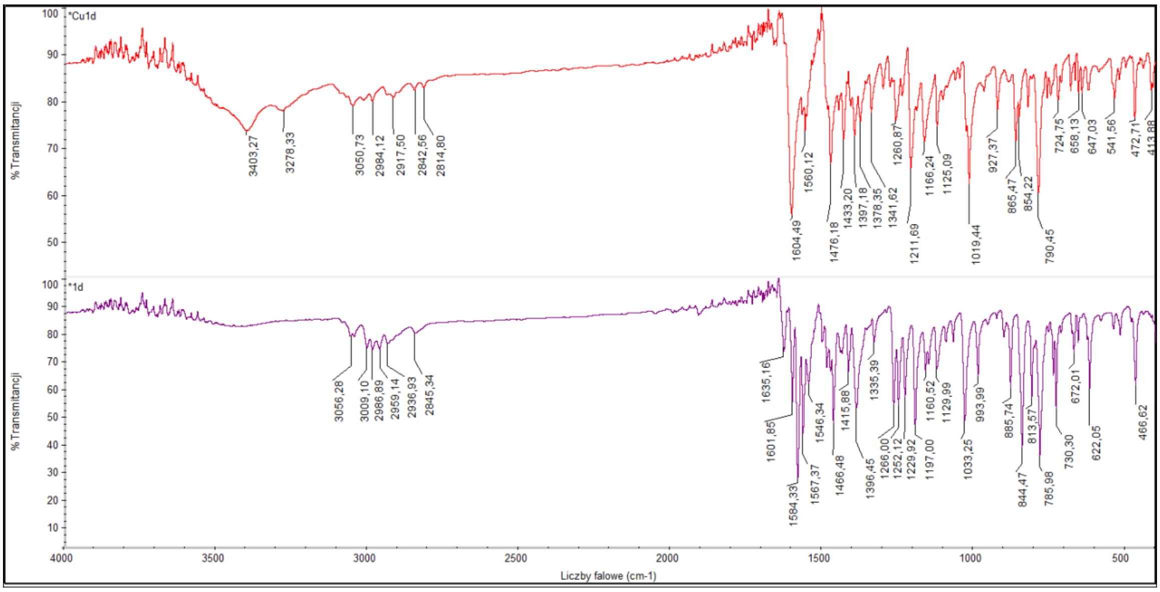
*Cu1d
*1d
% Transmitancji
Liczby falowe (cm-1)
4000
3500
3000
2500
2000
1500
1000
500
3403,27
3278,33
3050,73
2984,12
2917,50
2842,56
2814,80
1604,49
1560,12
1476,18
1433,20
1397,18
1378,35
1341,62
1260,87
1211,69
1166,24
1125,09
1019,44
927,37
865,47
854,22
790,45
724,75
658,13
647,03
541,56
472,71
413,88
3056,28
3009,10
2986,89
2959,14
2936,93
2845,34
1635,16
1601,85
1584,33
1567,37
1546,34
1466,48
1415,88
1396,45
1335,39
1266,00
1252,12
1229,92
1197,00
1160,52
1129,99
1033,25
993,99
885,74
844,47
813,57
785,98
730,30
672,01
622,05
466,62

Cu1d

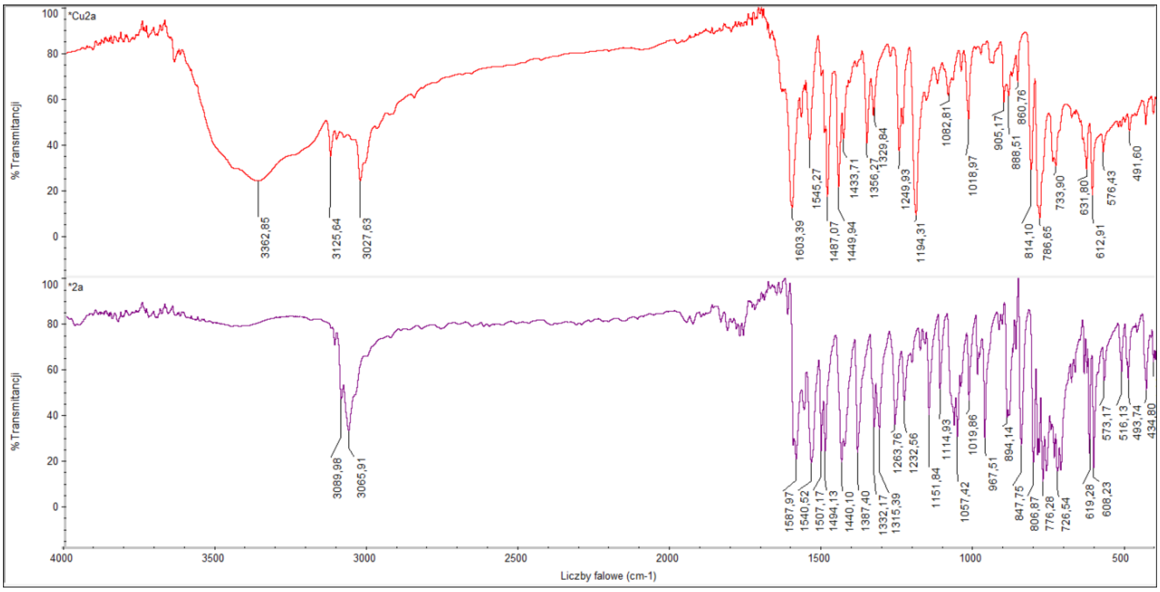
*Cu2a
*2a
% Transmitancji
Liczby falowe (cm-1)
4000
3500
3000
2500
2000
1500
1000
500
3362,85
3125,64
3027,63
1603,39
1545,27
1487,07
1449,94
1433,71
1356,27
1329,84
1249,93
1194,31
1082,81
1018,97
905,17
888,51
860,76
814,10
786,65
733,90
631,80
612,91
576,43
491,60
3089,98
3065,91
1587,97
1540,52
1507,17
1494,13
1440,10
1387,40
1332,17
1315,39
1263,76
1232,56
1151,84
1114,93
1057,42
1019,86
967,51
894,14
847,75
806,87
776,28
726,54
619,28
608,23
573,17
516,13
493,74
434,80

Cu2a

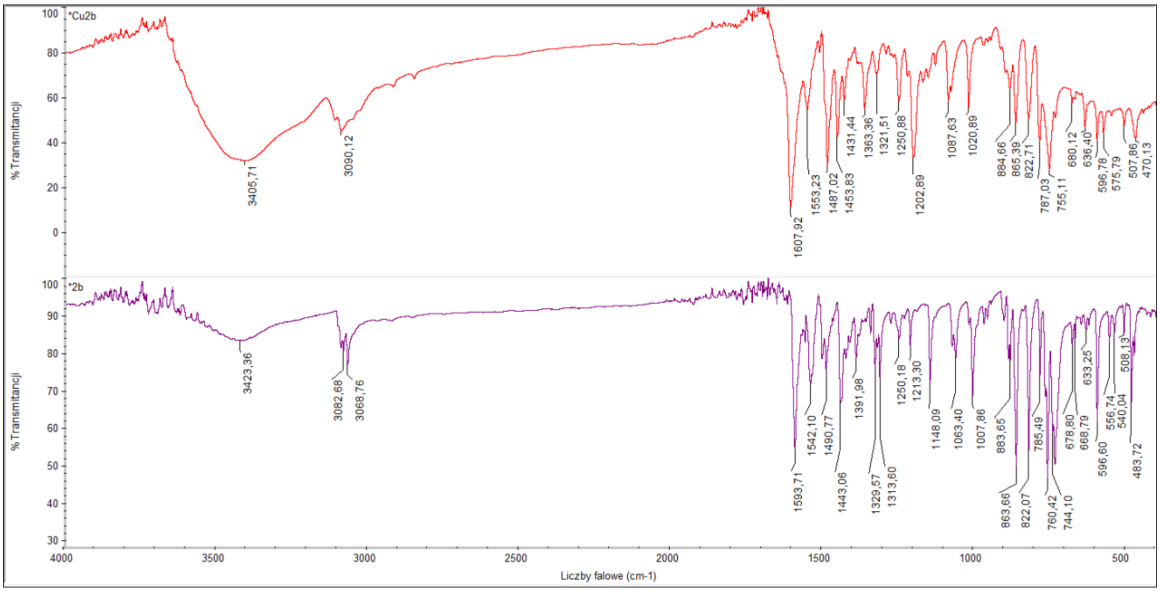
*Cu2b
*2b
% Transmitancji
Liczby falowe (cm-1)
3405,71
3090,12
1607,92
1553,23
1487,02
1453,83
1431,44
1363,36
1321,51
1250,88
1202,89
1087,63
1020,89
884,66
865,39
822,71
787,03
755,11
680,12
636,40
596,78
575,79
507,86
470,13
3423,36
3082,68
3068,76
1593,71
1542,10
1490,77
1443,06
1391,98
1329,57
1313,60
1250,18
1213,30
1148,09
1063,40
1007,86
883,65
863,66
822,07
785,49
760,42
744,10
678,80
668,79
633,25
596,60
556,74
540,04
508,13
483,72

Cu2b

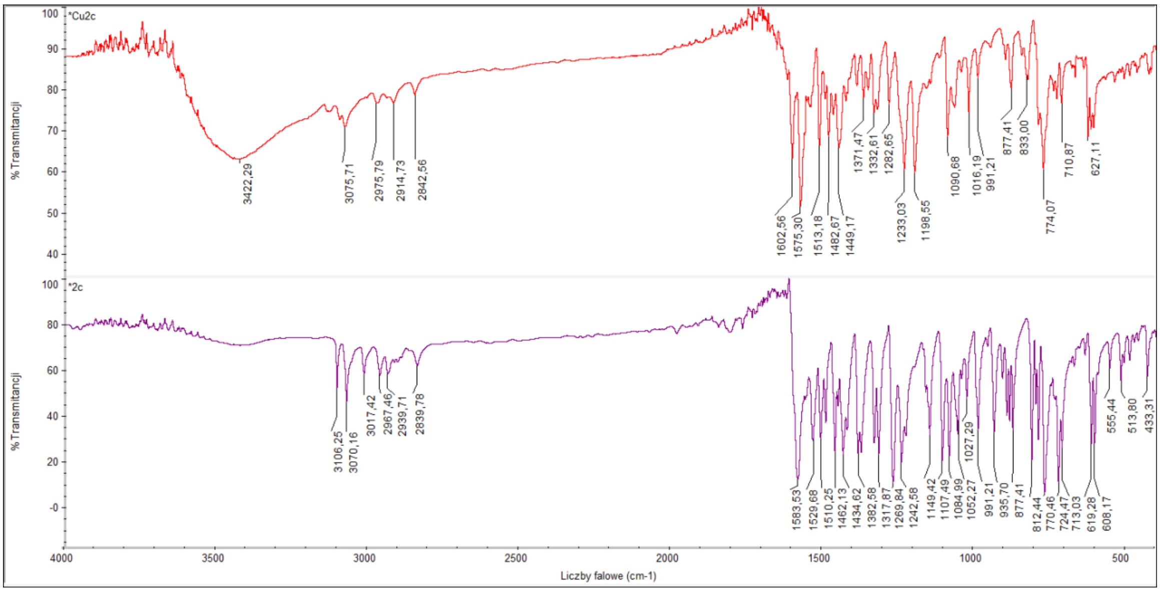
*Cu2c
*2c
% Transmitancji
Liczby falowe (cm-1)
3422,29
3075,71
2975,79
2914,73
2842,56
1602,56
1575,30
1513,18
1482,67
1449,17
1371,47
1332,61
1282,65
1233,03
1198,55
1090,68
1016,19
991,21
877,41
833,00
774,07
710,87
627,11
3106,25
3070,16
3017,42
2967,46
2939,71
2839,78
1583,53
1529,68
1510,25
1462,13
1434,62
1382,58
1317,87
1269,84
1242,58
1149,42
1107,49
1084,99
1052,27
1027,29
991,21
935,70
877,41
812,44
770,46
724,47
713,03
619,28
608,17
555,44
513,80
433,31

Cu2c

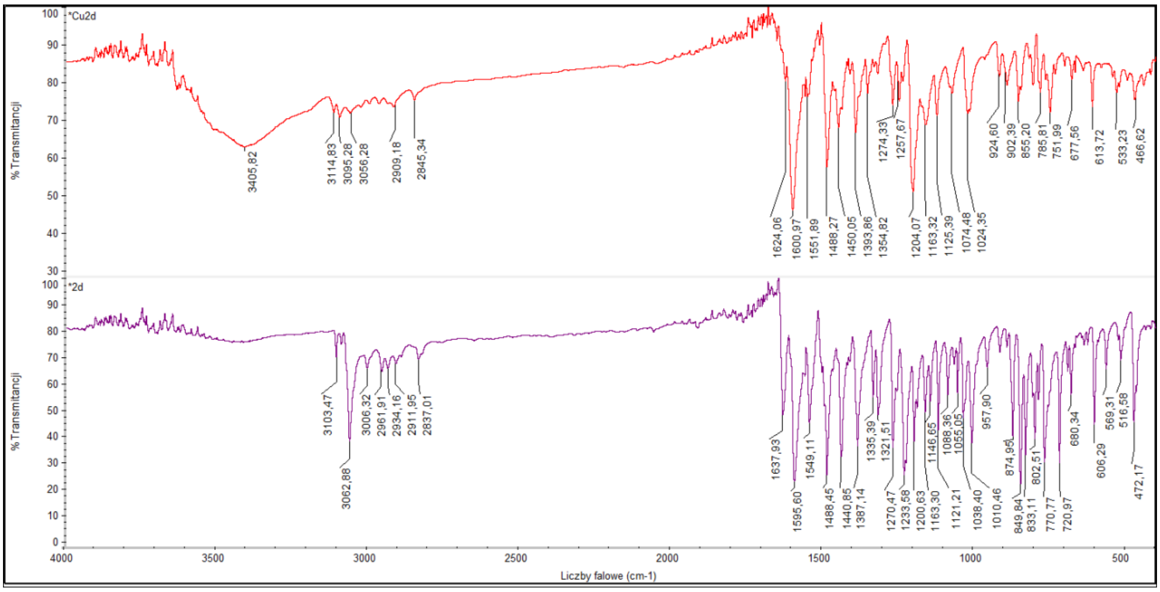

Cu2d

Figure S2. FT-IR spectra of studied Cu(II) compounds with reference to corresponding ligands.

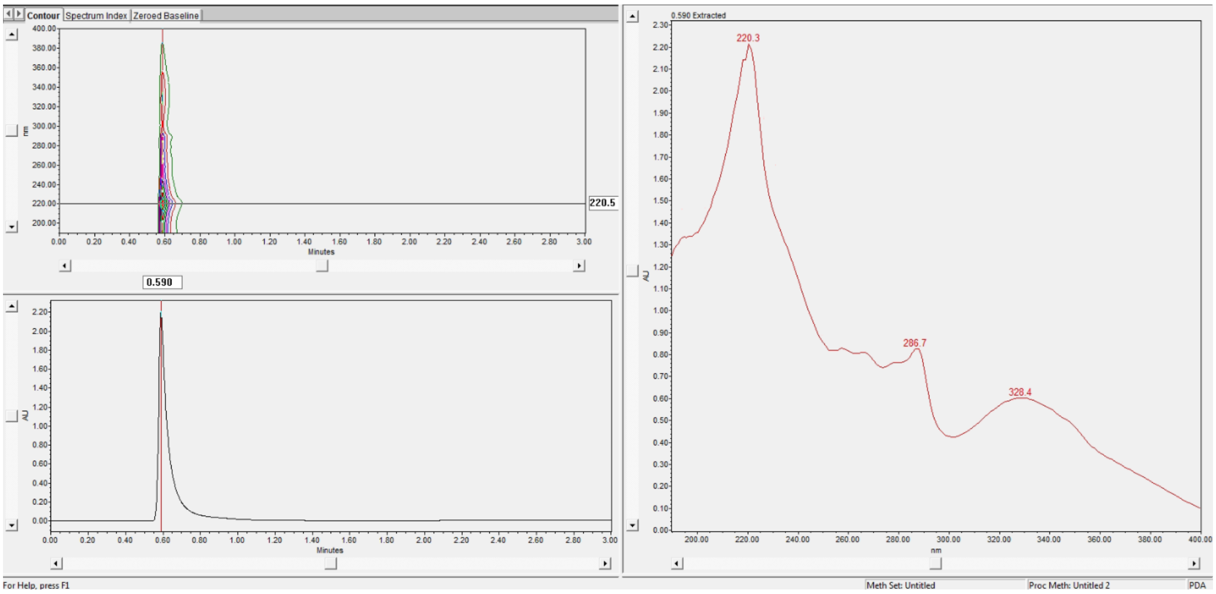
Contour
Spectrum Index
Zeroed Baseline
220.5
0.590
Minutes
AU
0.590 Extracted
220.3
286.7
328.4
nm
For Help, press F1
Meth Set: Untitled
Proc Meth: Untitled 2
PDA

Cu1a

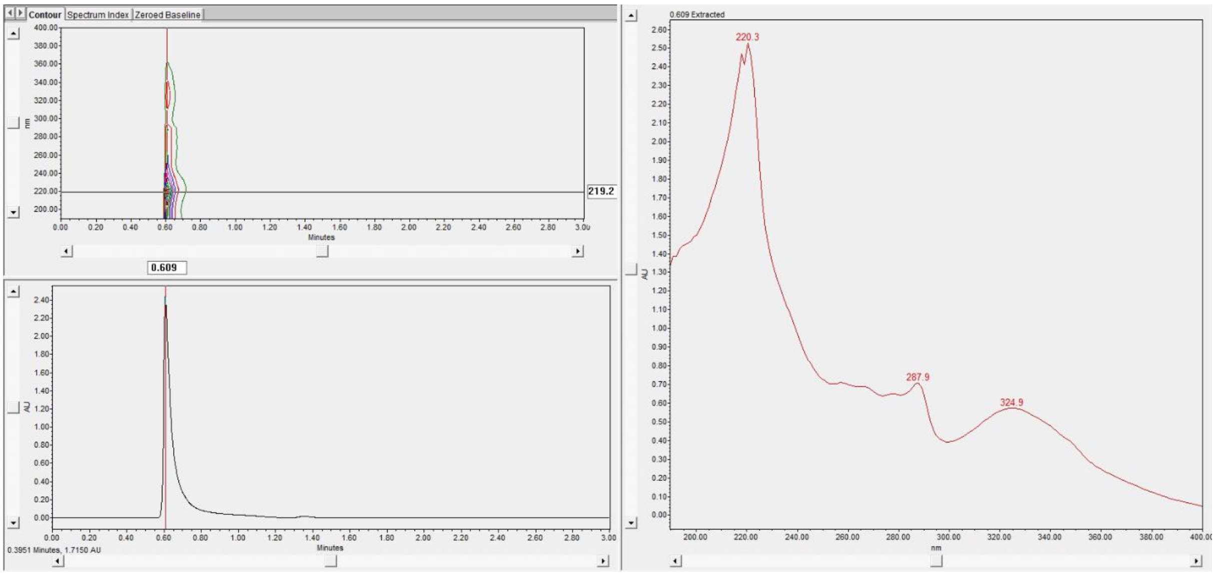
Contour
Spectrum Index
Zeroed Baseline
219.2
0.609
Minutes
AU
0.3951 Minutes, 1.7150 AU
0.609 Extracted
220.3
287.9
324.9
nm

Cu1b

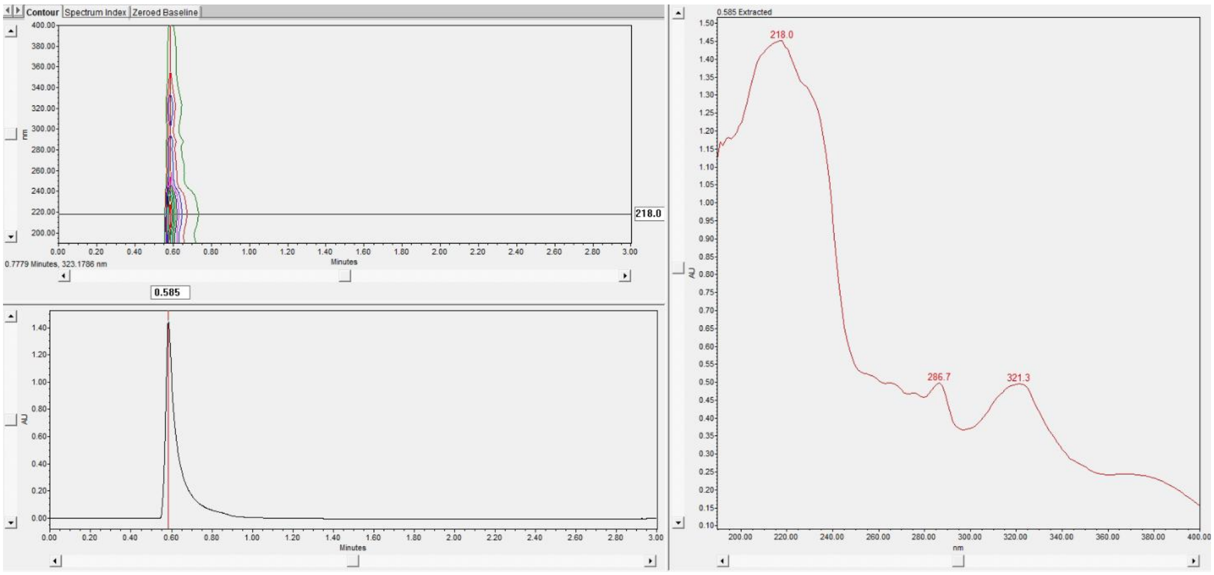

Cu1c

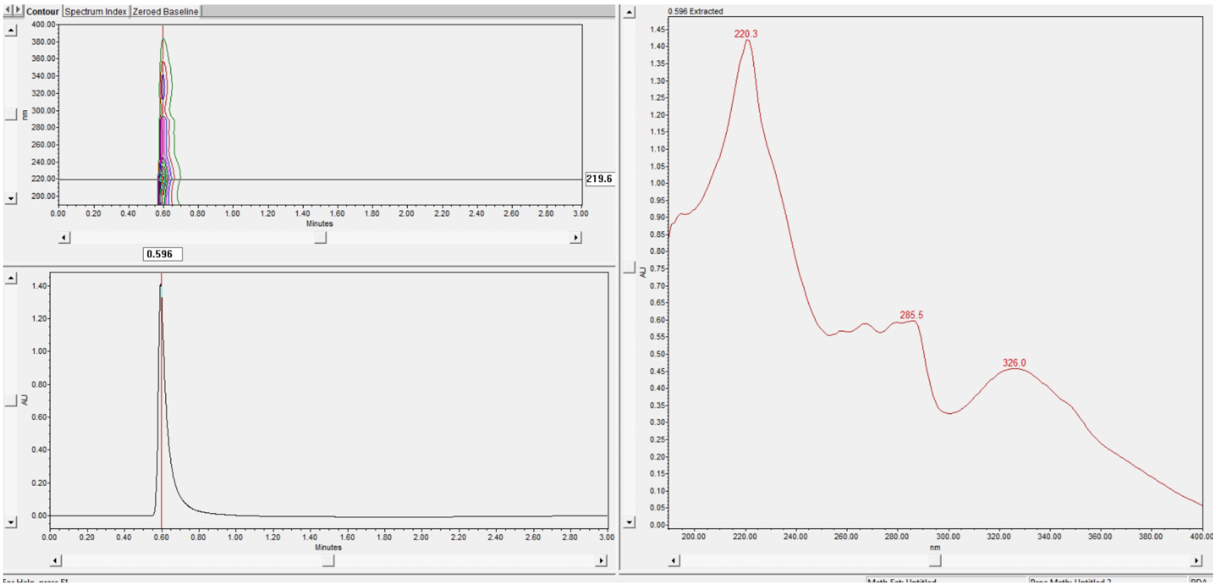

Cu1d

Figure S3. UPLC spectra of compounds Cu1a–Cu1d.

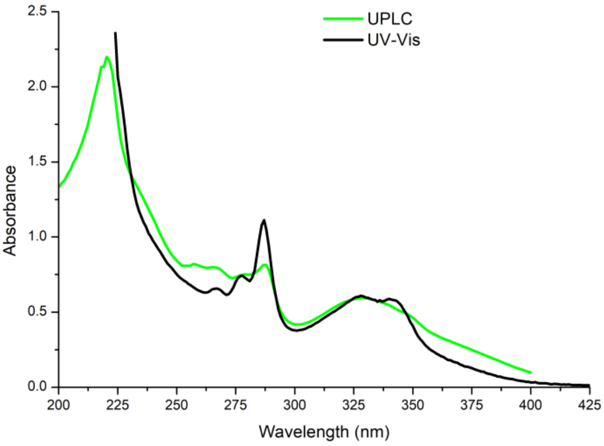

Cu1a, c = 50 µM

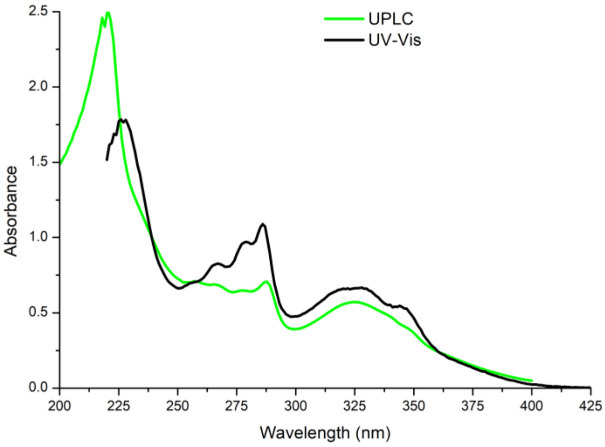

Cu1b, c = 50 µM

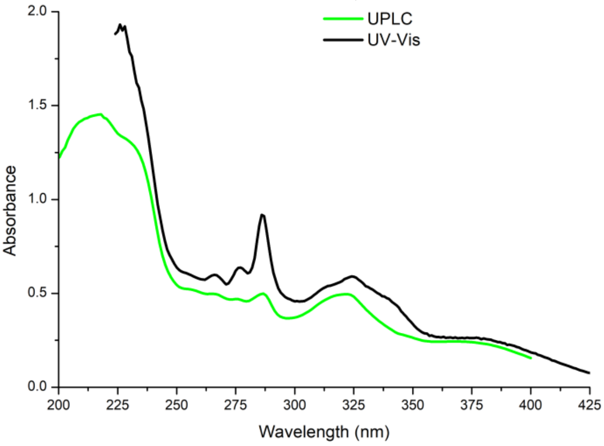

Cu1c, c = 50 µM

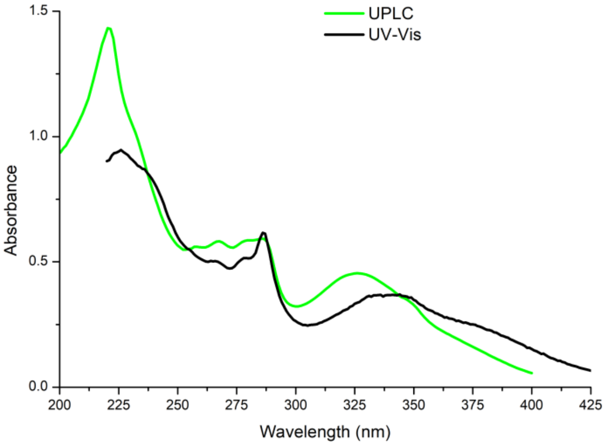

Cu1d, c = 25 µM

Figure S4. Comparison of the UV-Vis spectra of aqueous solutions of Cu1a-d (black, c = 25 µM or 50µM) and the spectra extracted from the UPLC detector (green).

3. Crystallography

Table S2. Crystal data and structure refinement.

Complex	Cu1a	Cu1b	Cu1d	Cu2a	Cu2b	Cu2c	Cu2d
Empirical formula	$C_{27}H_{25}Cl_2CuN_3O_2$	$C_{25}H_{17}Cl_2CuN_3$	$C_{26}H_{19}Cl_2CuN_3O$	$C_{21}H_{13}Cl_2CuN_3S_2$	$C_{21}H_{13}Cl_2CuN_3S_2$	$C_{44}H_{40}Cl_4Cu_2N_6O_7S_4$	$C_{22}H_{15}Cl_2CuN_3OS_2$
Formula weight	557.94	493.85	523.88	505.90	505.90	1173.65	535.93
Temperature [K]	295.0(2)	295.0(2)	295.0(2)	295.0(2)	295.0(2)	295.0(2)	295.0(2)
Wavelength [Å]	0.71073	0.71073	0.71073	0.71073	0.71073		0.71073
Crystal system	Monoclinic	Monoclinic	Monoclinic	Orthorhombic	Triclinic	Monoclinic	Monoclinic
Space group	$P2_1/c$	$P2_1/n$	$P2_1/c$	$Pca2_1$	$P\bar{1}$	$P2_1/c$	$P2_1/c$
	a = 8.2681(8)	a = 8.0676(3)	a = 8.5085(6)	a = 16.2077(6)	a = 10.3420(3)	a = 7.9113(4)	a = 9.6212(7)
	b = 38.594(3)	b = 24.7212(11)	b = 11.4177(7)	b = 11.4867(5)	b = 11.7862(4)	b = 23.6960(16)	b = 14.3807(9)
Unit cell dimensions [Å,°]	c = 8.3885(7)	c = 10.8646(5)	c = 24.5174(14)	c = 24.4275(10)	c = 21.3075(8)	c = 25.8626(9)	c = 18.3170(13)
					α = 104.219(3)		
	β = 108.027(9)	β = 104.772(5)	β = 91.109(6)		β = 96.464(3)	β = 96.976(4)	β = 91.785(7)
					γ = 102.085(3)		
Volume [$Å^3$]	2545.4(4)	2095.23(16)	2381.4(3)	4547.7(3)	2424.74(15)	4812.5(4)	2533.1(3)
Z	4	4	4	8	4	4	4
Density (calculated) [Mg/m^3]	1.456	1.566	1.461	1.478	1.386	1.620	1.405
Absorption coefficient [mm-1]	1.098	1.316	1.165	1.391	1.305	1.338	1.256
F(000)	1148	1004	1068	2040	1020	2394	1084
Crystal size [mm]	0.345 × 0.287 × 0.041	0.370 × 0.079× 0.070	0.269 × 0.093 × 0.034	0.166 × 0.126 × 0.106	0.211 × 0.074 × 0.046	0.214 × 0.152 × 0.121	0.560 × 0.050 × 0.050
θ range for data collection [°]	3.34 to 25.05	3.60 to 25.05	3.40 to 25.05	3.50 to 25.05	3.49 to 25.05	3.41 to 25.15	3.60 to 25.20
	$-9 \leq h \leq 9$	$-8 \leq h \leq 9$	$-10 \leq h \leq 10$	$-16 \leq h \leq 19$	$-12 \leq h \leq 12$	$-9 \leq h \leq 8$	$-11 \leq h \leq 11$
Index ranges	$-45 \leq k \leq 45$	$-24 \leq k \leq 29$	$-13 \leq k \leq 11$	$-13 \leq k \leq 11$	$-14 \leq k \leq 14$	$-22 \leq k \leq 28$	$-17 \leq k \leq 16$
	$-9 \leq l \leq 9$	$-12 \leq l \leq 12$	$-28 \leq l \leq 29$	$-27 \leq l \leq 29$	$-25 \leq l \leq 22$	$-30 \leq l \leq 26$	$-21 \leq l \leq 16$
Reflections collected	4474	9067	4186	7423	8554	8590	4542
Independent reflections	3824 (R_{int} = 0.0774)	3702 (R_{int} = 0.0289)	3258 (R_{int} = 0.0321)	6525 (R_{int} = 0.0382)	5781 (R_{int} = 0.0621)	6422 (R_{int} = 0.0433)	3568 (R_{int} = 0.0336)
Completeness to 2θ	99.7%	99.7%	99.5%	99.7%	99.7%	99.6%	99.6%
Min. and max. transm.	0.543 and 1.000	0.764 and 1.000	0.793 and 1.000	0.445 and 1.000	0.812 and 1.000	0.627 and 1.000	0.632 and 1.000

Data / restraints / parameters	4474 / 0 / 320	3702 / 0 / 280	4186 / 0 / 299	7423 / 1 / 523	8554 / 0 / 523	8590 / 0 / 606	4542 / 0 / 281
Goodness-of-fit on F^2	1.286	1.040	1.045	1.058	0.922	1.098	1.059
Final R indices [I>2σ(I)]	R_1 = 0.1004<br>wR_2 = 0.1894	R_1 = 0.0362<br>wR_2 = 0.0818	R_1 = 0.0351<br>wR_2 = 0.0828	R_1 = 0.0359<br>wR_2 = 0.0818	R_1 = 0.0433<br>wR_2 = 0.0904	R_1 = 0.0622<br>wR_2 = 0.1280	R_1 = 0.0445<br>wR_2 = 0.1179
R indices (all data)	R_1 = 0.1119<br>wR_2 = 0.1932	R_1 = 0.0491<br>wR_2 =0.0866	R_1 = 0.0512<br>wR_2 = 0.0887	R_1 = 0.0454<br>wR_2 = 0.0855	R_1 = 0.0670<br>wR_2 = 0.0982	R_1 = 0.0890<br>wR_2 = 0.1365	R_1 = 0.0583<br>wR_2 = 0.1239
Largest diff. peak and hole [e $Å^{-3}$]	0.72 and -0.62	0.28 and -0.30	0.29 and -0.28	0.44 and -0.23	0.29 and -0.51	0.76 and -0.64	0.64 and -0.34
CCDC number	2124050	2150148	2124051	2124052	2124054	2361049	2124053

Table S3. Selected bond lengths [Å] and angles [°].

Bond lengths [Å]							
**Complex**	**Cu1a**	**Cu1b**	**Cu1d**	**Cu2a**	**Cu2b**	**Cu2c**	**Cu2d**
**M = Cu**							
M(1)–Cl(1)	2.221(2)	2.2196(8)	2.2160(8)	2.2022(17)	2.2142(10)	2.2438(14)	2.2075(10)
M(1)–Cl(2)	2.643(2)	2.4919(8)	2.6063(7)	2.4935(16)	2.5280(10)	2.4363(16)	2.5220(10)
M(1)–N(1)	2.036(6)	2.065(2)	2.036(2)	2.057(5)	2.039(3)	2.076(4)	2.034(3)
M(1)–N(2)	1.946(6)	1.947(2)	1.9390(19)	1.964(5)	1.974(3)	1.978(4)	1.975(3)
M(1)–N(3)	2.033(6)	2.069(2)	2.038(2)	2.051(5)	2.056(3)	2.043(4)	2.057(3)
M(2)–Cl(3)				2.2053(19)	2.2209(10)	2.2409(14)	
M(2)–Cl(4)				2.5063(17)	2.5215(11)	2.4359(16)	
M(2)–N(4)				2.074(5)	2.045(3)	2.037(4)	
M(2)–N(5)				1.973(5)	1.974(3)	1.976(4)	
M(2)–N(6)				2.025(5)	2.056(3)	2.065(4)	
**Bond angles [º]**							
**Complex**	**Cu1a**	**Cu1b**	**Cu1d**	**Cu2a**	**Cu2b**	**Cu2c**	**Cu2d**
Cl(1)–M(1)–Cl(2)	103.98(8)	104.85(3)	103.03(3)	102.42(6)	106.87(3)	106.17(6)	104.03(4)
N(1)–M(1)–Cl(1)	99.31(18)	99.08(7)	100.02(6)	99.11(16)	99.92(9)	99.41(11)	99.35(9)
N(1)–M(1)–Cl(2)	92.58(19)	93.10(6)	95.70(6)	100.09(14)	93.49(9)	93.44(12)	91.69(8)
N(2)–M(1)–Cl(1)	169.51(19)	162.10(7)	168.15(6)	163.49(14)	160.14(8)	155.55(13)	162.23(8)
N(2)–M(1)–Cl(2)	86.52(18)	93.03(7)	88.82(6)	94.07(14)	92.99(8)	98.26(13)	93.69(8)
N(2)–M(1)–N(1)	80.0(2)	78.77(9)	78.94(8)	78.54(19)	78.13(11)	77.94(16)	78.49(11)
N(2)–M(1)–N(3)	79.5(2)	78.81(9)	78.92(8)	78.62(18)	78.81(11)	78.67(17)	78.44(11)
N(3)–M(1)–Cl(1)	99.75(18)	98.98(7)	99.33(6)	99.01(15)	98.33(8)	97.18(12)	99.48(9)
N(3)–M(1)–Cl(2)	91.66(19)	98.57(6)	94.90(6)	94.57(14)	97.63(8)	101.01(13)	99.88(8)
N(3)–M(1)–N(1)	158.8(2)	155.18(8)	155.19(8)	153.59(18)	154.85(10)	154.00(16)	154.76(11)
Cl(3)–M(2)–Cl(4)				104.73(6)	104.99(4)	103.69(6)	
N(4)–M(2)–Cl(3)				99.69(15)	99.19(9)	97.17(12)	
N(4)–M(2)–Cl(4)				93.11(13)	95.08(9)	102.64(13)	
N(5)–M(2)–Cl(3)				162.54(14)	158.46(9)	153.90(13)	
N(5)–M(2)–Cl(4)				92.72(14)	96.55(9)	102.36(12)	
N(5)–M(2)–N(4)				78.41(19)	78.53(11)	78.53(17)	
N(5)–M(2)–N(6)				78.5(2)	78.29(11)	78.29(16)	
N(6)–M(2)–Cl(3)				98.19(17)	99.61(8)	98.42(11)	
N(6)–M(2)–Cl(4)				101.69(14)	94.87(8)	93.77(12)	
N(6)–M(2)–N(4)				153.02(19)	155.65(11)	153.96(16)	

Table S4. Short intra- and intermolecular hydrogen bonds.

D–H•••A	D–H [Å]	H•••A [Å]	D–A [Å]	D–H•••A [°]
**Cu1a**				
O(1)–H(1A)•••Cl(2)	0.82	2.31	3.098(12)	161.0
O(2)–H(2A)•••Cl(2)	0.82	2.28	3.086(10)	166.0
C(2)–H(2)•••Cl(1)[a]	0.93	2.82	3.749(9)	173.0
C(4)–H(4)•••O(2)[b]	0.93	2.56	3.486(11)	173.0
C(7)–H(7)•••O(2)[b]	0.93	2.42	3.344(11)	176.0
**Cu1b**				
C(3)–H(3)•••Cl(2)[c]	0.93	2.82	3.540 (3)	135.0
C(25)–H(25)•••Cl(2)[d]	0.93	2.78	3.673(3)	161.0
**Cu1d**				
C(2)–H(2)•••O(1)[e]	0.93	2.44	3.322(4)	158.0
C(4)–H(4)•••Cl(2)[f]	0.93	2.78	3.691(3)	166.0
C(15)–H(15)•••Cl(1)	0.93	2.82	3.383(3)	120.0
C(25)–H(25)•••Cl(2)[f]	0.93	2.70	3.608(3)	164.0
**Cu2a**				
C(2)–H(2)•••Cl(4)	0.93	2.70	3.520(7)	148.0
C(10)–H(10)•••Cl(3)[g]	0.93	2.72	3.547(6)	148.0
C(18)–H(18)•••Cl(3)[h]	0.93	2.76	3.613(7)	153.0
C(23)–H(23)•••Cl(1)[i]	0.93	2.77	3.577(8)	146.0
C(23)–H(23)•••Cl(2)[i]	0.93	2.82	3.507(9)	132.0
C(31)–H(31)•••Cl(2)	0.93	2.75	3.666(7)	168.0
**Cu2b**				
C(2)–H(2)•••Cl(3)	0.93	2.81	3.412(5)	124.0
C(7)–H(7)•••Cl(2)[j]	0.93	2.81	3.712(4)	164.0
C(23)–H(23)•••Cl(2)[j]	0.93	2.71	3.626(4)	168.0
C(42)–H(42)•••Cl(4)[k]	0.93	2.82	3.718(4)	161.0
**Cu2c**				
O(3)–H(3A)•••O(4)	0.85	2.02	2.812(12)	154.0
O(4)–H(4A)•••O(5)[d]	0.85	2.01	2.844(11)	167.0
O(4)–H(4B)•••O(7)	0.85	2.18	2.962(14)	153.0
O(5)–H(5B)•••Cl(2)	0.85	2.43	3.223(8)	155.0
O(64)–H(6B)•••O(7)[l]	0.85	2.45	3.120(16)	136.0
C(13)–H(13)•••Cl(3)	0.93	2.68	3.604(5)	174.0
C(17)–H(17)•••O(1)	0.93	2.40	2.726(8)	100.0
C(35)–H(35)•••Cl(1)	0.93	2.78	3.699(5)	170.0
C(39)–H(39)•••O(2)	0.93	2.38	2.712(8)	101.0
**Cu2d**				
C(7)–H(7)•••Cl(2)[m]	0.93	2.79	3.697(3)	164.0

Symmetry codes: (a) = 2-x,1-y,1-z; (b) = x,y,-1+z; (c) = 2-x,1-y,2-z; (d) = -1+x,y,z; (e) = -1+x,3/2-y,-1/2+z, (f) = -x,1-y,1-z; (g) = 1/2+x,1-y,z; (h) = x,1+y,z; (i) = -1/2+x,-y,z; (j) = -x,1-y,-z; (k) = 1-x,-y,1-z; (l) = -1-x,1/2+y,1/2-z; (m) = 1-x,-y,-z.

Table S5. Short π•••π interactions.

Cg(I)•••Cg(J)	Cg(I)•••Cg(J) [Å]	α [°]	β [°]	γ [°]	Cg(I)–Perp [Å]	Cg(J)–Perp [Å]
**Cu1a**<br>no interactions detected						
**Cu1b**<br>Cg3 is the centroid of atoms N(1)/C(1)/C(2)/C(3)/C(4)/C(5); Cg5 is the centroid of atoms N(3)/C(11)/C(12)/C(13)/C(14)/C(15); Cg6 is the centroid of atoms C(16)/C(17)/C(18)/C(19)/C(24)/C(25)						
Cg(3)•••Cg(3)[a]	3.6975(17)	0.00(14)	25.7	25.7	3.3323(12)	3.3322(12)
Cg(3)•••Cg(5)[b]	3.8139(17)	7.12(14)	24.7	27.6	3.3787(12)	3.4648(12)
Cg(5)•••Cg(6)[c]	3.9977(18)	8.21(14)	23.5	31.5	3.4073(12)	3.6674(12)
**Cu1d**<br>Cg4 is the centroid of atoms N(2)/C(6)/C(7)/C(8)/C(9)/C(10); Cg6 is the centroid of atoms C(16)/C(17)/C(18)/C(19)/C(24)/C(25)						
Cg(4)•••Cg(6)[d]	3.6523(14)	1.55(12)	20.9	22.2	3.3804(10)	3.4120(10)
**Cu2a**<br>Cg3 is the centroid of atoms S(1)/C(2)/C(1)/N(1)/C(3); Cg10 is the centroid of atoms S(3)/C(23)/C(22)/N(4)/C(24); Cg4 is the centroid of atoms S(2)/C(9)/N(3)/C(11)/C(10); Cg11 is the centroid of atoms S(4)/C(30)/N(6)/C(32)/C(31)						
Cg(3)•••Cg(10)[e]	3.794(4)	6.3(3)	28.1	23.6	3.476(3)	3.346(3)
Cg(4)•••Cg(10)[f]	3.990(4)	3.8(3)	36.2	32.3	3.372(3)	3.221(3)
Cg(4)•••Cg(11)[e]	3.817(4)	3.7(3)	25.6	29.3	3.328(3)	3.442(2)
**Cu2b**<br>Cg4 is the centroid of atoms S(2)/C(9)/N(3)/C(11)/C(10); Cg5 is the centroid of atoms N(2)/C(4)/C(5)/C(6)/C(7)/C(8); Cg6 is the centroid of atoms C(12)/C(13)/C(14)/C(15)/C(20)/C(21); Cg7 is the centroid of atoms C(15)/C(16)/C(17)/C(18)/C(19)/C(20); Cg12 is the centroid of atoms S(4)/C(30)/N(6)/C(32)/C(31); Cg13 is the centroid of atoms N(5)/C(25)/C(26)/C(27)/C(28)/C(29); Cg14 is the centroid of atoms C(33)/C(34)/C(35)/C(36)/C(41)/C(42); Cg15 is the centroid of atoms C(36)/C(37)/C(38)/C(39)/C(40)/C(41)						
Cg(4)•••Cg(5)[g]	3.9432(19)	7.53(17)	26.5	24.5	3.5885(13)	3.5302(15)
Cg(5)•••Cg(6)[h]	3.631(2)	7.80(16)	18.7	21.3	3.3827(13)	3.4389(14)
Cg(5)•••Cg(7)[h]	3.688(2)	7.71(17)	21.1	23.5	3.3833(13)	3.4410(16)
Cg(12)•••Cg(13)[i]	3.8350(18)	3.15(16)	24.7	27.8	3.3929(14)	3.4841(13)
Cg(13)•••Cg(14)[j]	3.637(2)	1.87(16)	20.7	21.2	3.4760(13)	3.4881(16)
Cg(13)•••Cg(15)[j]	3.729(2)	0.86(17)	4.6	4.3	3.4648(13)	3.4634(12)
**Cu2c**<br>Cg3 is the centroid of atoms S(1)/C(1)/C(2)/N(1)/C(3); Cg5 is the centroid of atoms N(2)/C(4)/C(5)/C(6)/C(7)/C(8); Cg6 is the centroid of atoms C(12)/C(13)/C(14)/C(15)/C(16)/C(21); Cg7 is the centroid of atoms C(16)/C(17)/C(18)/C(19)/C(20)/C(21); Cg11 is the centroid of atoms S(3)/C(23)/C(24)/N(4)/C(25); Cg12 is the centroid of atoms S(4)/C(31)/N(6)/C(33)/C(32); Cg13 is the centroid of atoms N(5)/C(26)/C(27)/C(28)/C(29)/C(30); Cg14 is the centroid of atoms C(34)/C(35)/C(36)/C(37)/C(38)/C(43); Cg15 is the centroid of atoms C(38)/C(39)/C(40)/C(41)/C(42)/C(43)						
Cg(3)•••Cg(12)[c]	3.669(3)	0.8(2)	26.3	25.8	3.3026(19)	3.288(2)
Cg(3)•••Cg(13)[f]	3.686(3)	7.4(2)	31.4	24.7	3.3489(19)	3.147(2)
Cg(5)•••Cg(12)[f]	3.707(3)	6.5(2)	25.2	31.1	3.174(2)	3.355(2)
Cg(5)•••Cg(15)[k]	3.916(3)	21.7(3)	28.2	18.9	3.704(2)	3.452(2)
Cg(6)•••Cg(14)[l]	3.813(3)	10.0(3)	18.6	16.8	3.651(2)	3.615(2)
Cg(7)•••Cg(13)[k]	3.881(3)	20.6(3)	19.5	22.2	3.594(2)	3.658(2)
Cg(11)•••Cg(11)[m]	3.495(3)	0.0(3)	10.6	10.6	3.434(2)	3.435(2)
**Cu2d**<br>Cg4 is the centroid of atoms S(2)/C(9)/N(3)/C(11)/C(10); Cg5 is the centroid of atoms N(2)/C(4)/C(5)/C(6)/C(7)/C(8); Cg6 is the centroid of atoms C(12)/C(13)/C(14)/C(15)/C(20)/C(21); Cg7 is the centroid of atoms C(15)/C(16)/C(17)/C(18)/C(19)/C(20)						
Cg(4)•••Cg(5)[n]	3.9531(19)	7.88(16)	21.8	28.2	3.4838(13)	3.6715(13)
Cg(5)•••Cg(6)[o]	3.7490(18)	5.44(15)	27.4	22.0	3.4753(13)	3.3290(13)
Cg(5)•••Cg(7)[o]	3.7131(19)	5.45(16)	19.3	20.5	3.4784 (13)	3.5039(13)

α = dihedral angle between Cg(I) and Cg(J); Cg(I)–Perp = perpendicular distance of Cg(I) on ring J; Cg(J)–Perp = perpendicular distance of Cg(J) on ring I; β = angle between Cg(I)→Cg(J) vector and normal to ring I; γ = angle between Cg(I)→Cg(J) vector and normal to plane J

symmetry codes: (a)= 2-x,1-y,2-z; (b) = 2-x,1-y,1-z; (c) = 1+x,y,z; (d) = 1-x,1-y,1-z; (e) = x,-1+y,z; (f) = x,y,z; (g) = -x,1-y,-z; (h) = 1-x,1-y,-z; (i) = 1-x,-y,1-z; (j) = 2-x,-y,1-z; (k) = x,3/2-y,-1/2+z; (l) = -1+x,3/2-y,-1/2+z; (m) = -x,2-y,1-z; (n) = 1-x,-y,-z; (o) = -x,-y,-z;

Table S6. Y–X•••Cg(J)(π-ring) interactions.

Y–X(I)•••Cg(J)	X(I)•••Cg(J) [Å]	X–Perp [Å]	γ [°]	Y–X(I)•••Cg(J) [°]
**Cu1a**<br>Cg7 is the centroid of atoms C(20)/C(21)/C(22)/C(23)/C(24)/C(25)				
C(27)–H(27C)•••Cg(7)[a]	2.90	-2.81	14.50	130.00
**Cu1b**<br>Cg4 is the centroid of atoms N(2)/C(6)/C(7)/C(8)/C(9)/C(10)				
Cu(1)–Cl(1)•••Cg(4)[b]	3.6614(14)	-3.350	23.79	119.61(4)
**Cu1d**<br>no interactions detected				
**Cu2a**<br>Cg13 is the centroid of atoms N(5)/C(25)/C(26)/C(27)/C(28)/C(29)				
Cu(1)–Cl(1)•••Cg(13)[c]	3.625(3)	-3.463	17.22	95.22(8)
**Cu2b**<br>Cg7 is the centroid of atoms C(15)/C(16)/C(17)/C(18)/C(19)/C(20); Cg4 is the centroid of atoms S(2)/C(9)/N(3)/C(11)/C(10)				
C(37)–H(37)•••Cg(7)[d]<br>Cu(2)–Cl(3)•••Cg(4)[e]	2.73<br>3.9409(19)	-2.68<br>3.780	10.14<br>16.41	152.00<br>145.06(5)
**Cu2c**<br>Cg4 is the centroid of atoms S(2)/C(9)/N(3)/C(10)/C(11); Cg11 is the centroid of atoms S(3)/C(23)/C(24)/N(4)/C(25);				
Cu(1)–Cl(2)•••Cg(4)[f]<br>Cu(2)–Cl(4)•••Cg(11)[g]	3.834(3)<br>3.833(3)	-3.487<br>3.555	24.57<br>21.99	166.45(6)<br>166.27(7)
**Cu2d**<br>no interactions detected				

γ = angle between X(I)→Cg(J) vector and normal to plane J.
Symmetry codes: (a) = x,y,1+z; (b) = 2-x,1-y,1-z; (c) = x,1+y,z; (d) = 1+x,y,1+z; (e) = x,-1+y,z; (f) = 1+x,y,z; (g) = -1+x,y,z

Table S7. Ring-metal interactions Cg•••Me

Cg(I)•••Me(J)	Cg(I)•••Me(J) [Å]	Me(J)–Perp [Å]	β [°]
**Cu1a**<br>no interactions detected			
**Cu1b**<br>no interactions detected			
**Cu1d**<br>Cg7 is the centroid of atoms C(19)/C(20)/C(21)/C(22)/C(23)/C(24)			
Cg(7)•••Cu(1)[a]	3.755	3.742	4.75
**Cu2a**<br>Cg4 is the centroid of atoms S(2)/C(9)/N(3)/C(11)/C(10)			
Cg(4)•••Cu(1)[b]	3.904	-3.672	19.87
**Cu2b**<br>no interactions detected			
**Cu2c**<br>Cg3 is the centroid of atoms S(1)/C(1)/C(2)/N(1)/C(3); Cg12 is the centroid of atoms S(4)/C(31)/N(6)/C(33)/C(32)			
Cg(3)•••Cu(2)[c]<br>Cg(12)•••Cu(1)[c]	3.803<br>3.800	-3.479<br>3.459	23.80<br>24.48
**Cu2d**<br>no interactions detected			

Me(J)–Perp = perpendicular distance of Me(J) on ring I; β = angle Cg(I)→Me(J) vector and normal to ring I
Symmetry codes: (a) = 1-x,1-y,1-z; (b) = x,1+y,z; (c) = x,y,z.

Table S8. Selected structural parameters in square pyramidal Cu(II) complexes with 4´-R-terpy and 4-R-dtpy

**terpy**				**Ref**	**dtpy**				**Ref**
**Compound**	**Cu-Cl$_{axial}$ [Å]**	**τ_5**	**<R-terpy**		**Compound**	**Cu-Cl$_{axial}$ [Å]**	**τ_5**	**<R-dtpy**	
Cu1a	2.643(2)	0.178	48.24	this work	Cu2a	2.4935(16)	0.158<br>0.165	58.94<br>69.94	this work
Cu1b	2.4919(8)	0.115	3.96	this work	Cu2b	2.5280(10)<br>2.5215(11)	0.089<br>0.046	7.70<br>1.36	this work
Cu1c	2.4704(9)	0.01	30.02	[5]	Cu2c	2.4363(16)<br>2.4359(16)	0.025<br>0.002	39.14<br>36.90	this work
Cu1d	2.6063(7)	0.216	1.55	this work	Cu2d	2.5220(10)	0.126	5.45	this work
[$CuCl_2$(terpy)]	2.4700(3)	0.03	—	[15]	[$CuCl_2$(dtpy)]	2.4460(8)	0.02	—	[17]
[$CuCl_2$(terpy)]·H_2O	2.565(2)	0.08	—	[16]					
[$CuCl_2$(Ph-terpy)]	2.4410(7)	0.00	30.39	[18]	[$CuCl_2$(Ph-dtpy)]	2.466(1)<br>2.460(1)	0,07	5.16<br>9.95	[20]
[$CuCl_2$(Ph-terpy)]·2H_2O	2.505(2)	0.05	9.16	[19]					
[$CuCl_2$(Ph-terpy)]·CH_3OH	2.4491(9)<br>2.556(1)	0.05<br>0.11	27.08<br>27.15						
[$CuCl_2$(4´-furan-terpy)]	2.6007(8)	0.13	4.62	[10]	[$CuCl_2$(4-furan-dtpy)]	2.4782(8)	0.08	4.02	[10]
[$CuCl_2$(4´-thiophene-terpy)]	2.5631(13)	0.07	5.13	[10]	$CuCl_2$(4-thiophene-dtpy)]	2.4643(10)	0.06	4.52	[10]
[$CuCl_2$(4´-N-methylpyrrole-terpy)]*	2.4894(8)<br>2.4008(8)*	0.08<br>0.46*	19.12<br>11.82*	[10]	[$CuCl_2$(4-N-methylpyrrole-terpy)]	2.5796(14)	0.12	14.72	[10]
[$CuCl_2$(4´-(2-quinoline)-terpy]	2.5300(7)	0.09	7.92	[11]	[$CuCl_2$(4-(2-quinoline)-dtpy]	2.4966(13)	0.09	13.91	[11]
[$CuCl_2$(4´-(4-quinoline)-terpy)]	2.5050(15)	0.06	53.95	[11]	[$CuCl_2$(4-(4-quinoline)-dtpy)]	2.4332	0.00	49.10	[11]

* trigonal bipyramidal (instead of square pyramidal) geometry of Cu(II) center

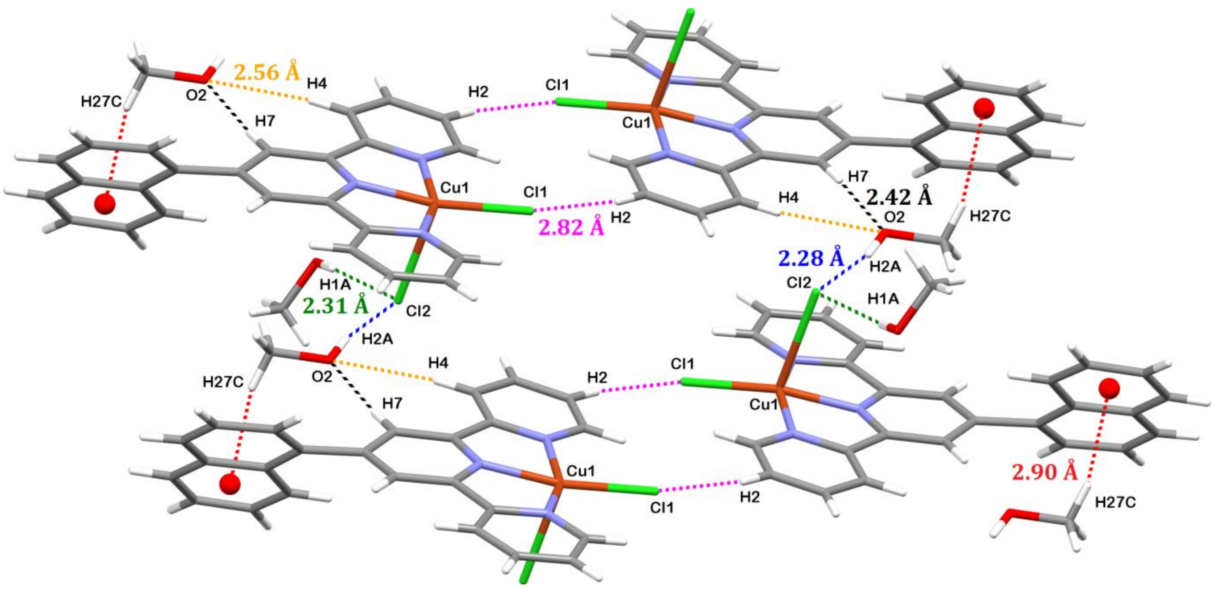

Figure S5. Perspective view showing intermolecular interactions in Cu1a.

4. UV-Vis Spectra and Stability in DMSO and PBS

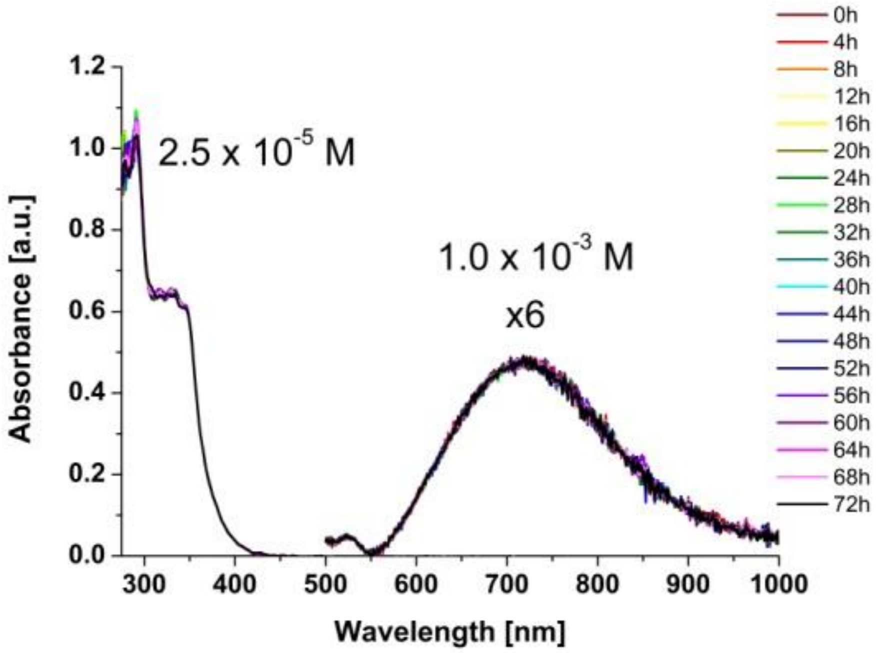

DMSO

Cu1a

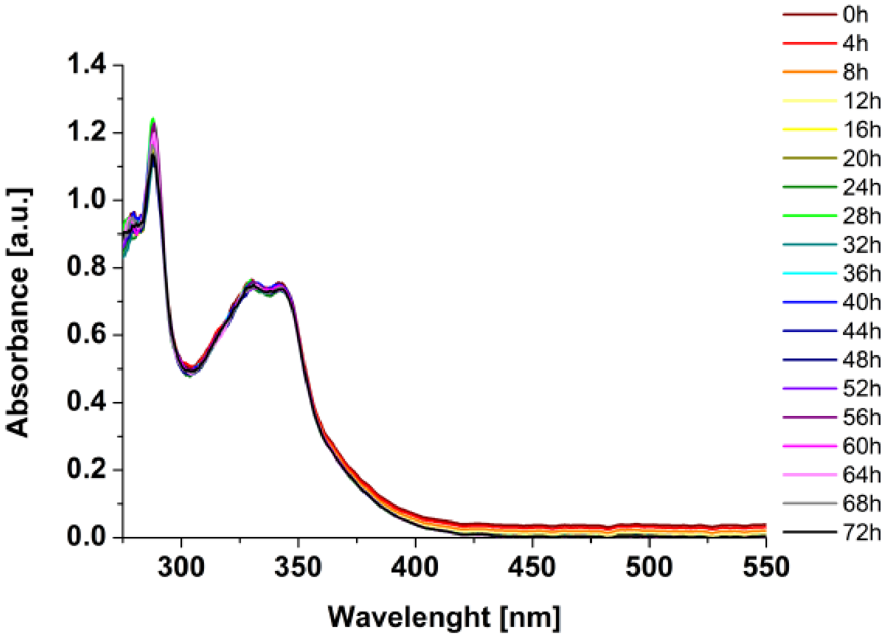

PBS

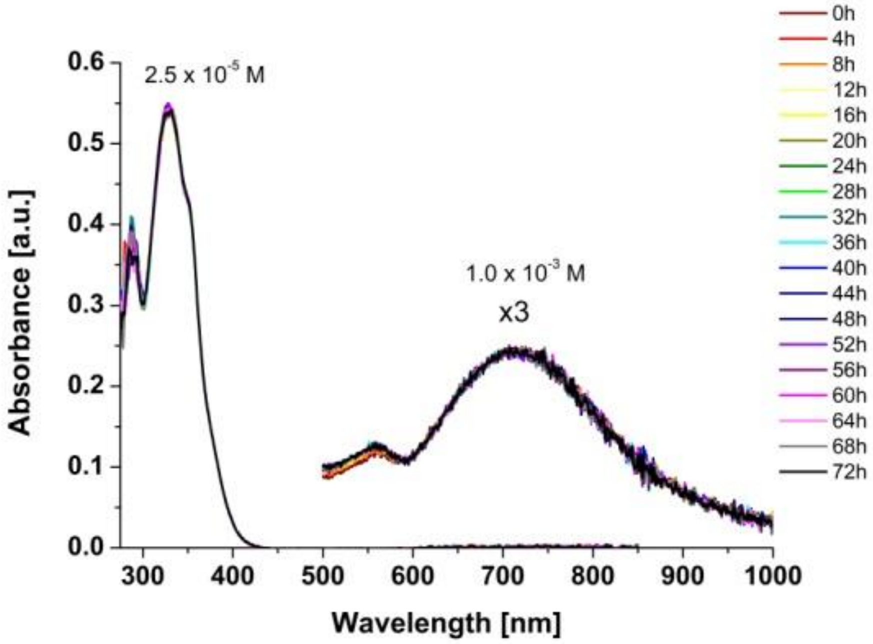

DMSO

Cu1b

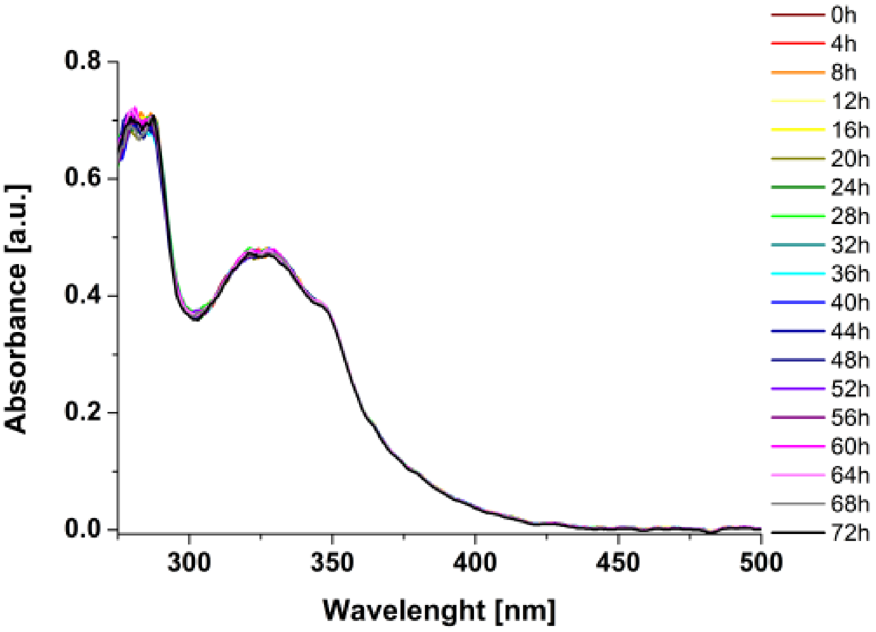

PBS

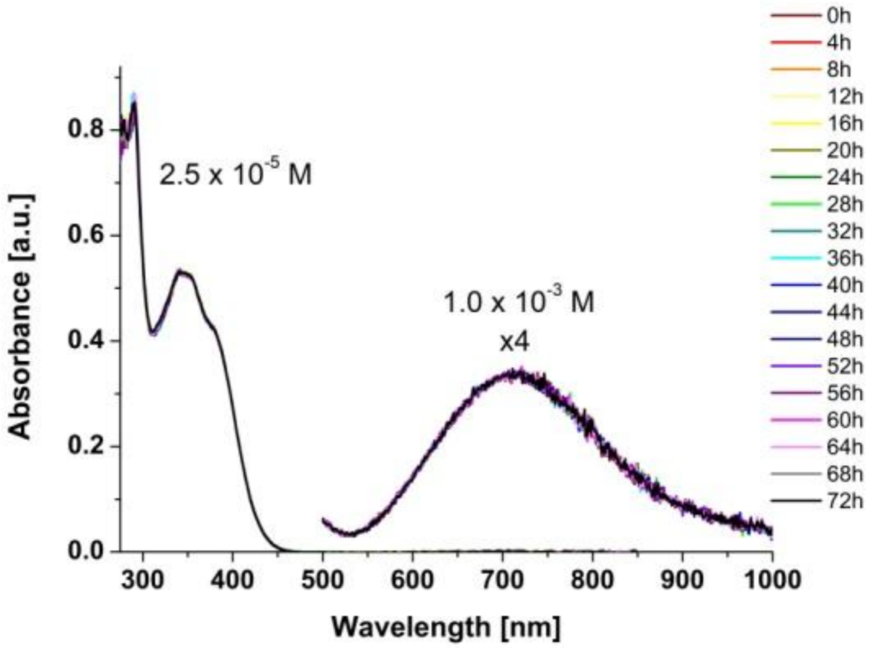

DMSO

Cu1d

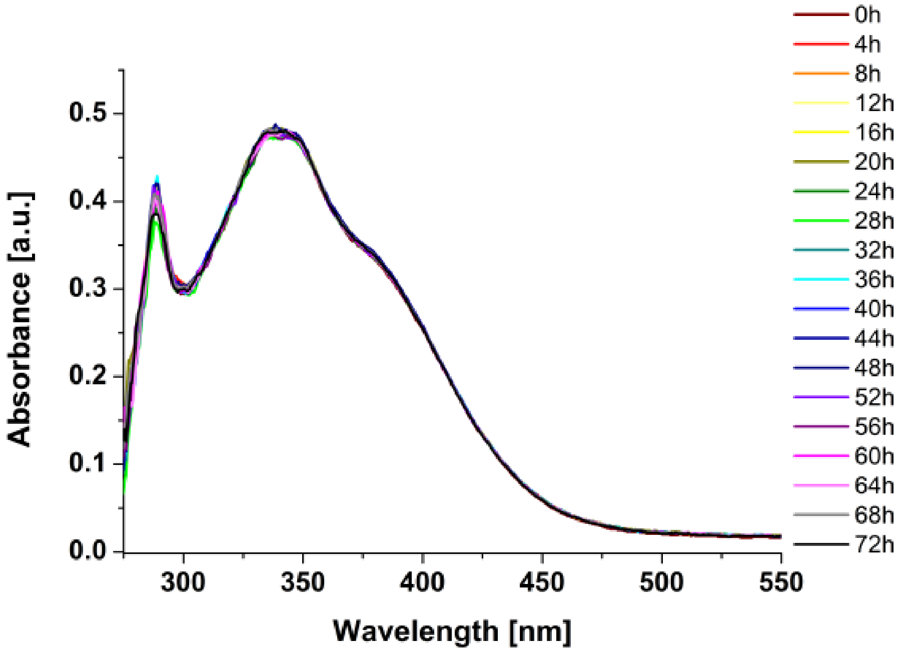

PBS

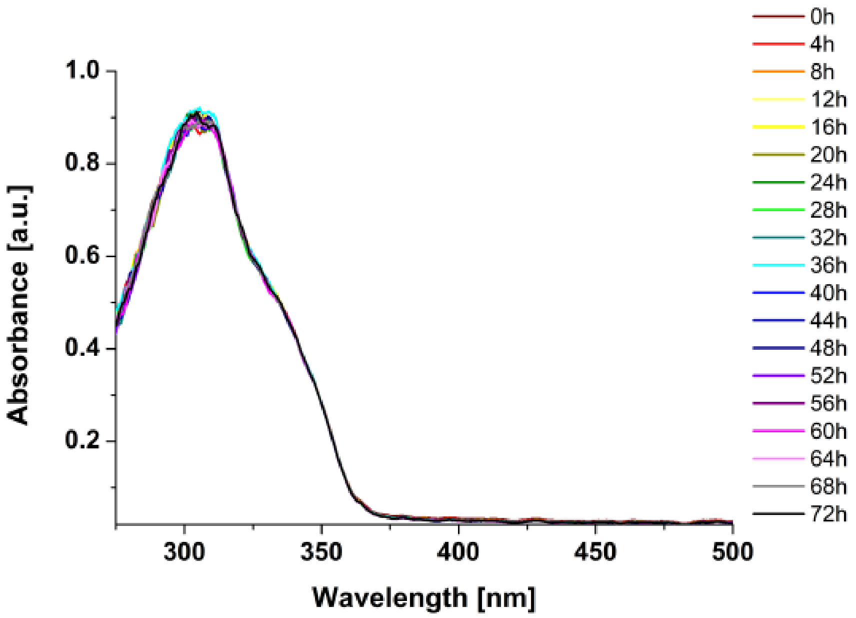

DMSO

Cu2a

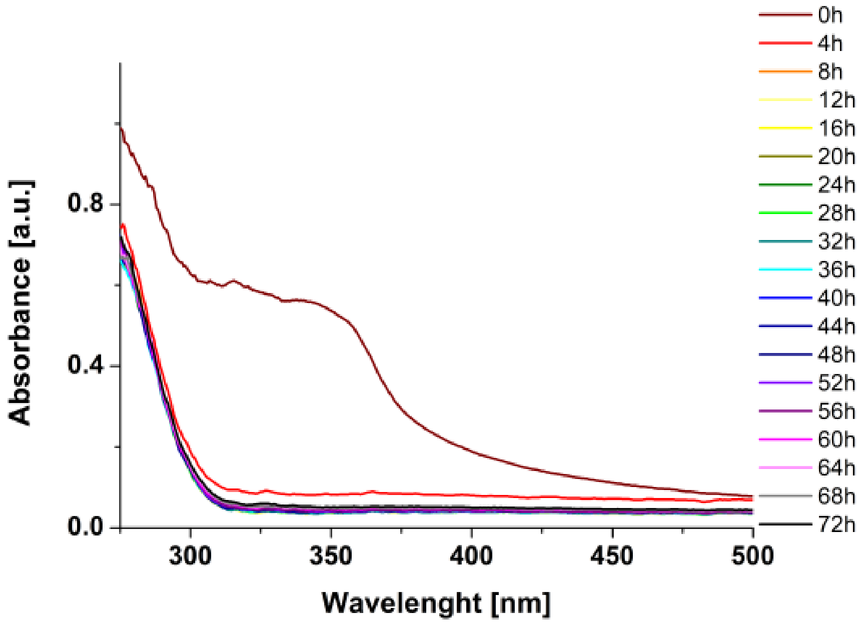

PBS

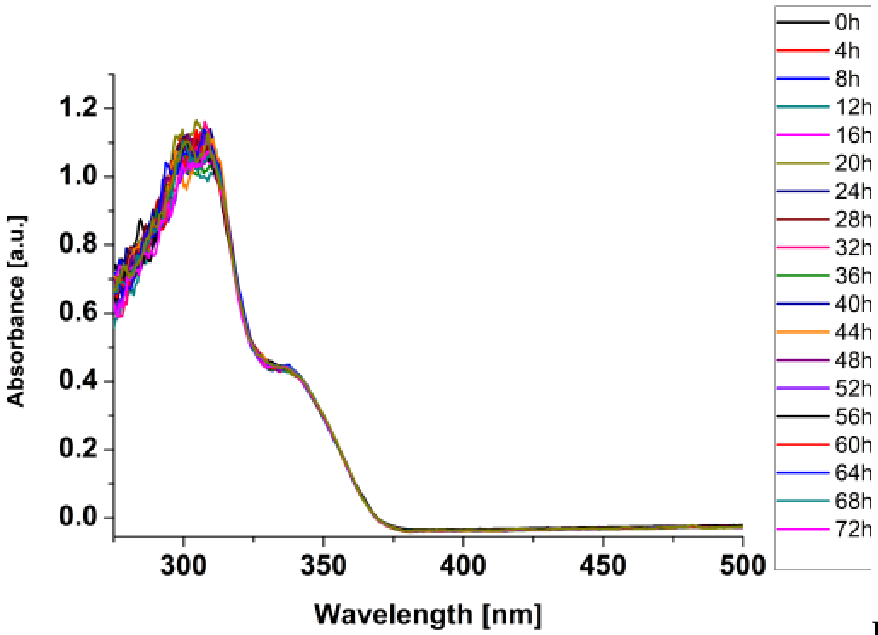

DMSO

Cu2b

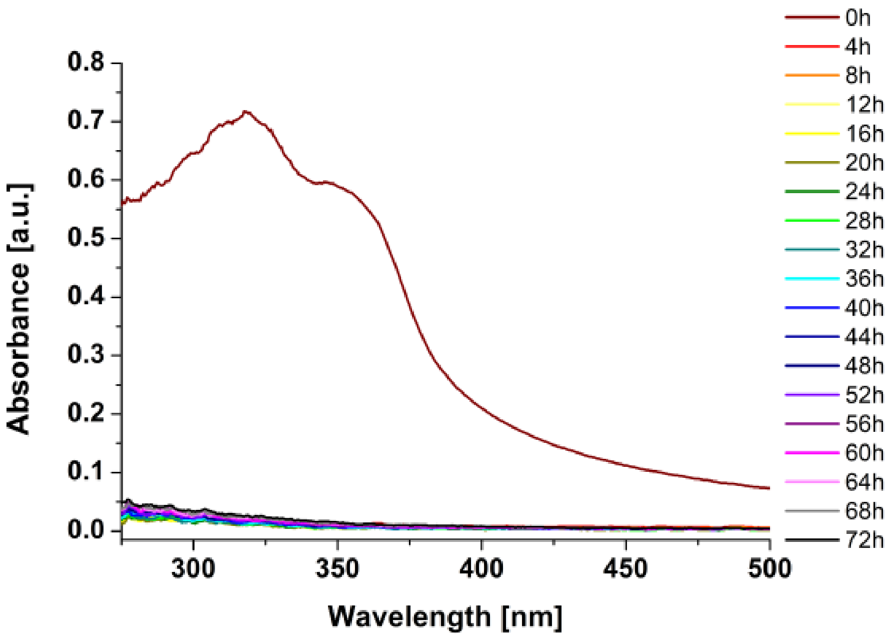

PBS

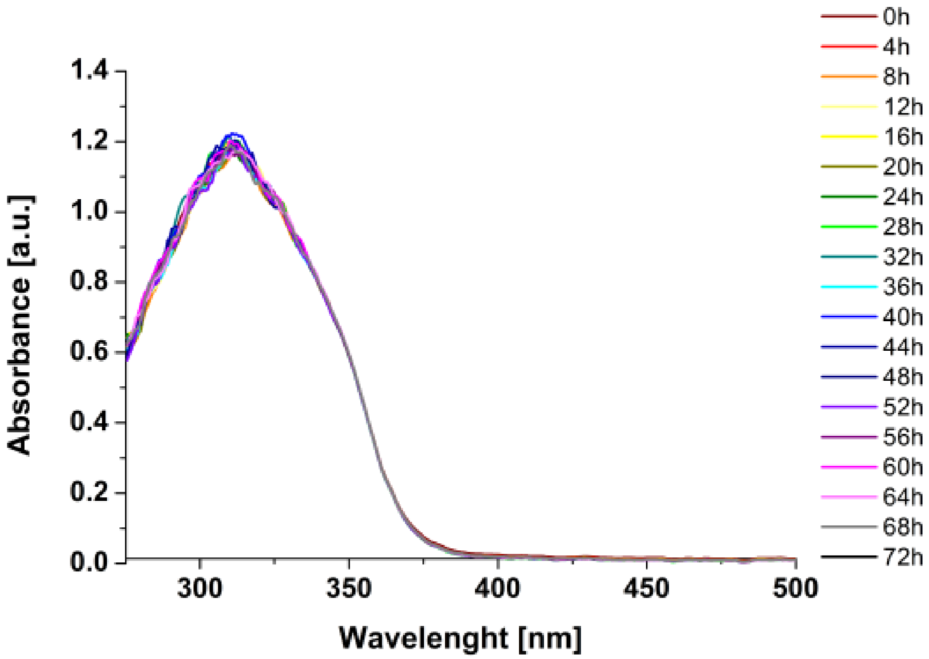

DMSO

Cu2c

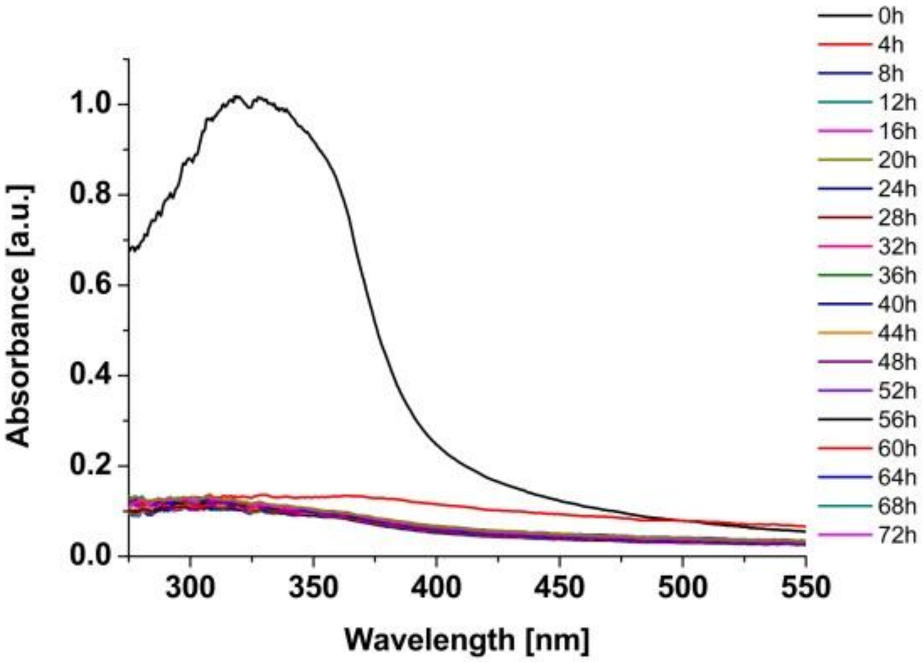

PBS

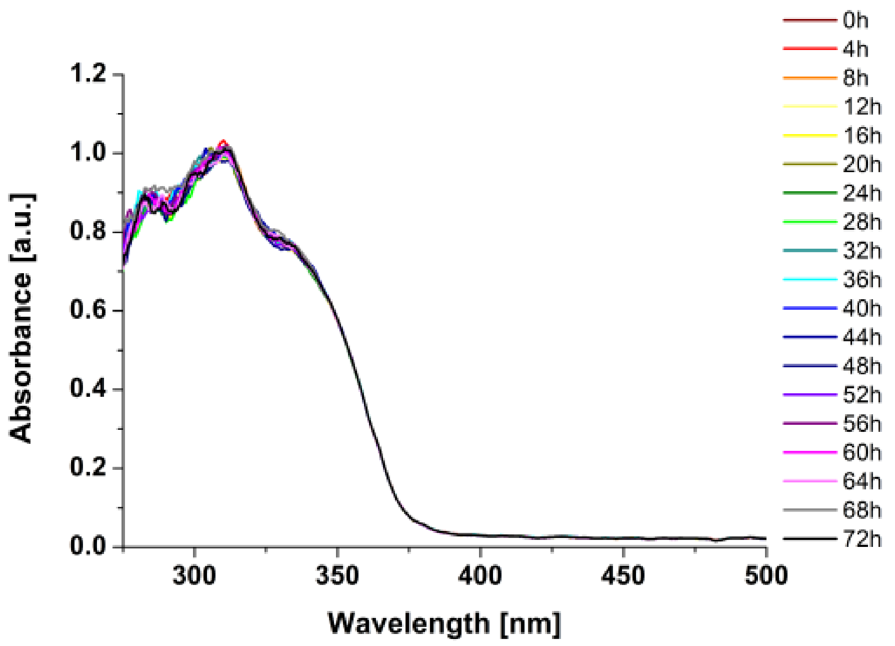

DMSO

Cu2d

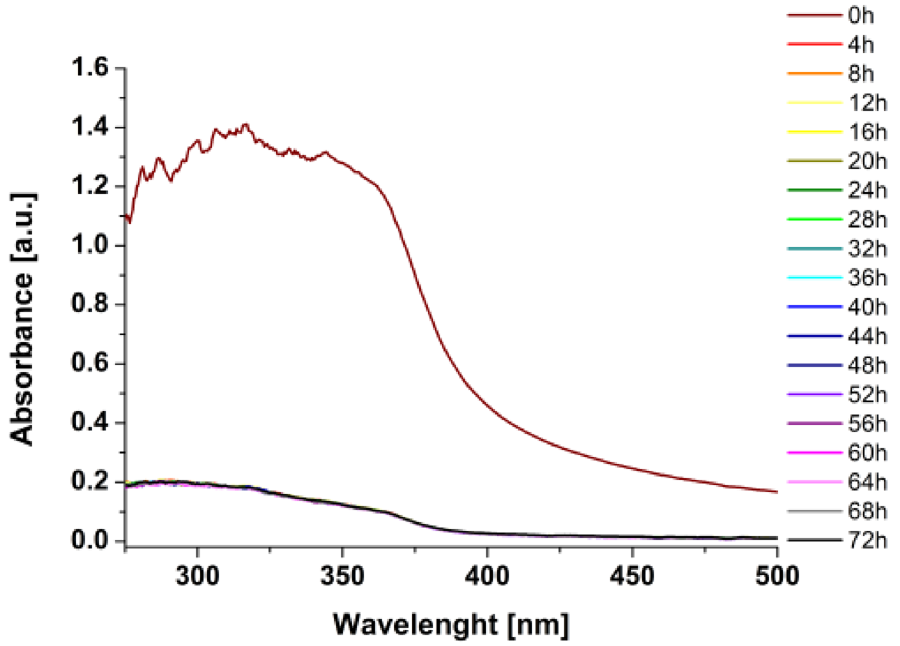

PBS

Figure S6. Stability studies of Cu1a-b, Cu1d and Cu2a-d compounds in DMSO and PBS solutions.

Cu1a

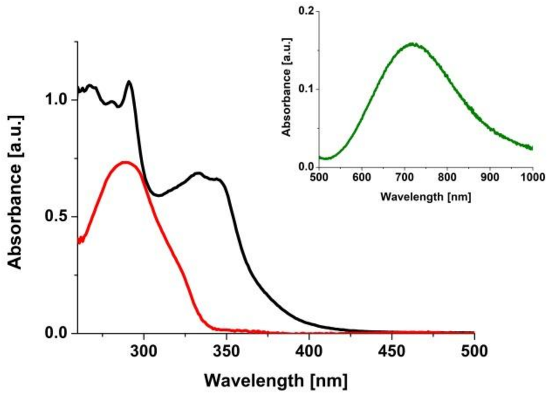

Cu1b

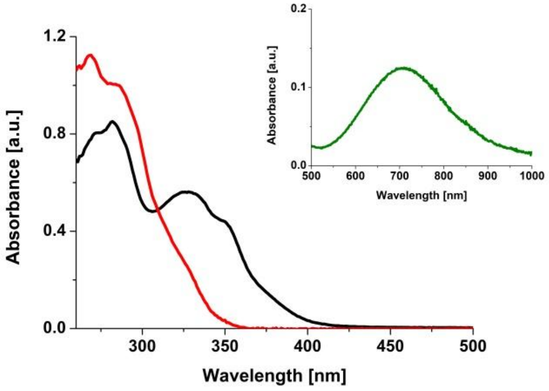

Cu1d

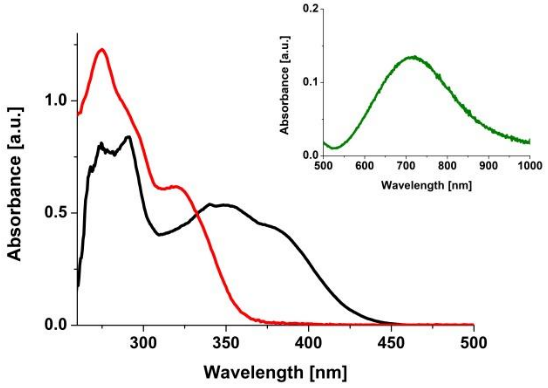

Cu2a

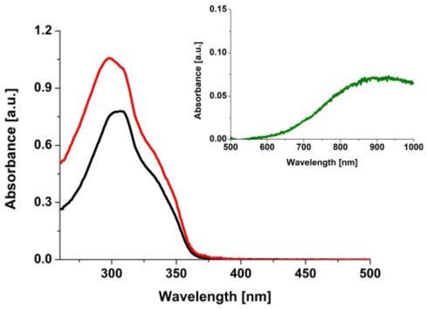

Cu2b

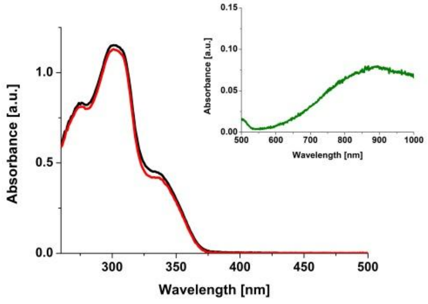

Cu2c

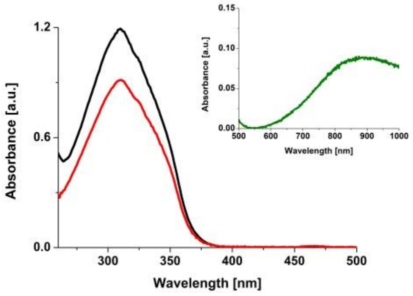

Cu2d

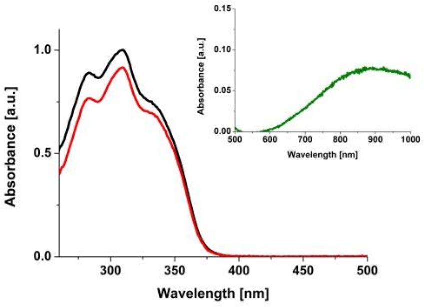

Figure S7. Electronic absorption spectra of compounds Cu1a, b and d and Cu2a-d (black line) in comparison to the corresponding organic ligands (red line) in DMSO solution (c – 25µM) ; inset plots: electronic absorption spectra of copper complexes in concentrated DMSO solution (c – 1mM) (green line).

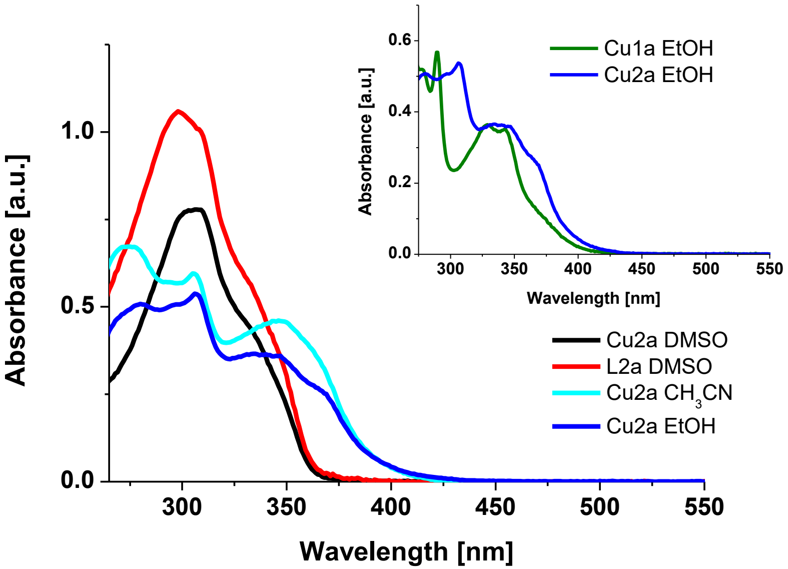

Figure S8a. Electronic absorption spectra of compounds Cu2a in DMSO (c – 25µM, black line), CH_3CN (c – 25µM, light blue), EtOH (c – 25µM, blue) with comparison to the corresponding organic ligands (red line) in DMSO solution (c – 25µM), inset plots: comparison of Cu2a and Cu1a absorption spectra in EtOH solutions (c – 25µM).

<table>
<tr>
<td>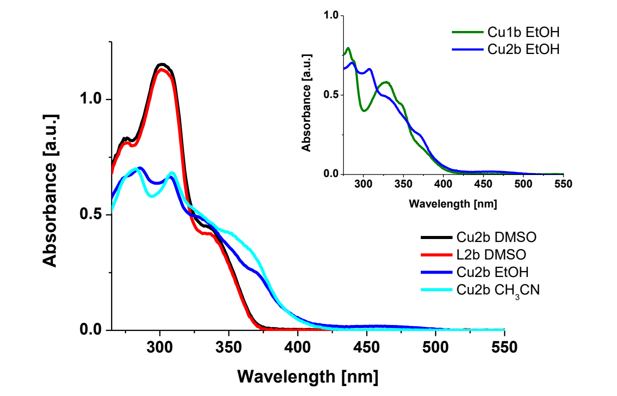
</td>
<td>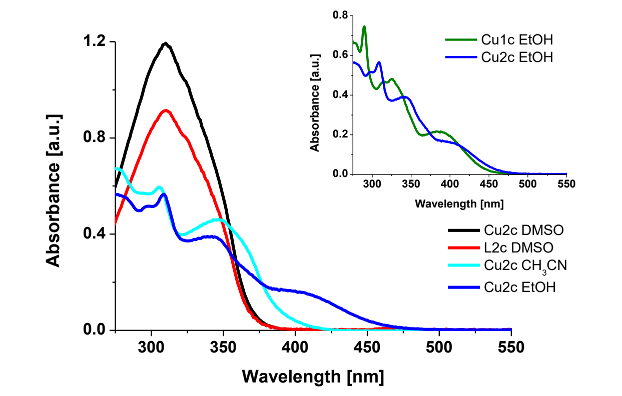
</td>
<td>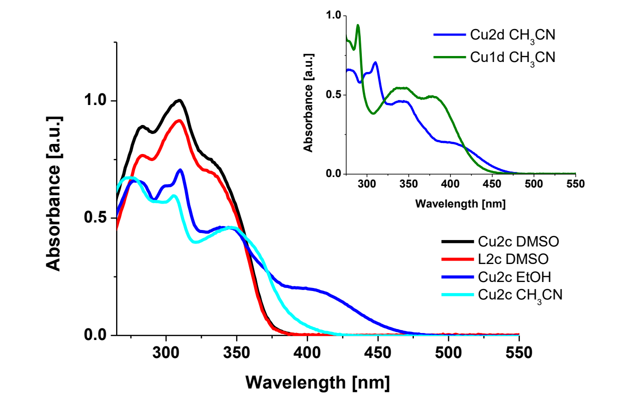
</td>
</tr>
<tr>
<td>Cu2b</td>
<td>Cu2c</td>
<td>Cu2d</td>
</tr>
</table>

Figure S8b. Electronic absorption spectra of compounds Cu2b-d in DMSO (c – 25μM, black line), CH_3CN (c – 25μM, light blue), EtOH (c – 25μM, blue) with comparison to the corresponding organic ligands (red line) in DMSO solution (c – 25μM), inset plots: comparison of Cu2b-d and Cu1b-d absorption spectra in EtOH solutions (c – 25μM).

Table S9. Electronic absorption maxima of complexes Cu1a-b, c and Cu2a-d

	λ [nm] (ε [$M^{-1}cm^{-1}$])	
Cu1a	350 (17840), 327 (22720), 282 (34160), 272 (32160)	709 (126)
Cu1b	344 (26560), 333 (26720), 291 (43160), 280 (39720), 267 (42640)	720 (158)
Cu1d	379 (17480), 348 (21560), 340 (21680), 291 (33840), 274 (32200)	713 (135)
Cu2a	336 (18080), 302 (46200), 276 (33440)	893 (79)
Cu2b	332 (17720), 308 (31120)	921 (71)
Cu2c	324 (41520), 310 (47840)	888 (88)
Cu2d	332 (30000), 309 (40120), 283 (35720)	886 (77)

5. Cell Viability Assays: HCT116, HCT116-DoxR, A2780 and Fibroblasts

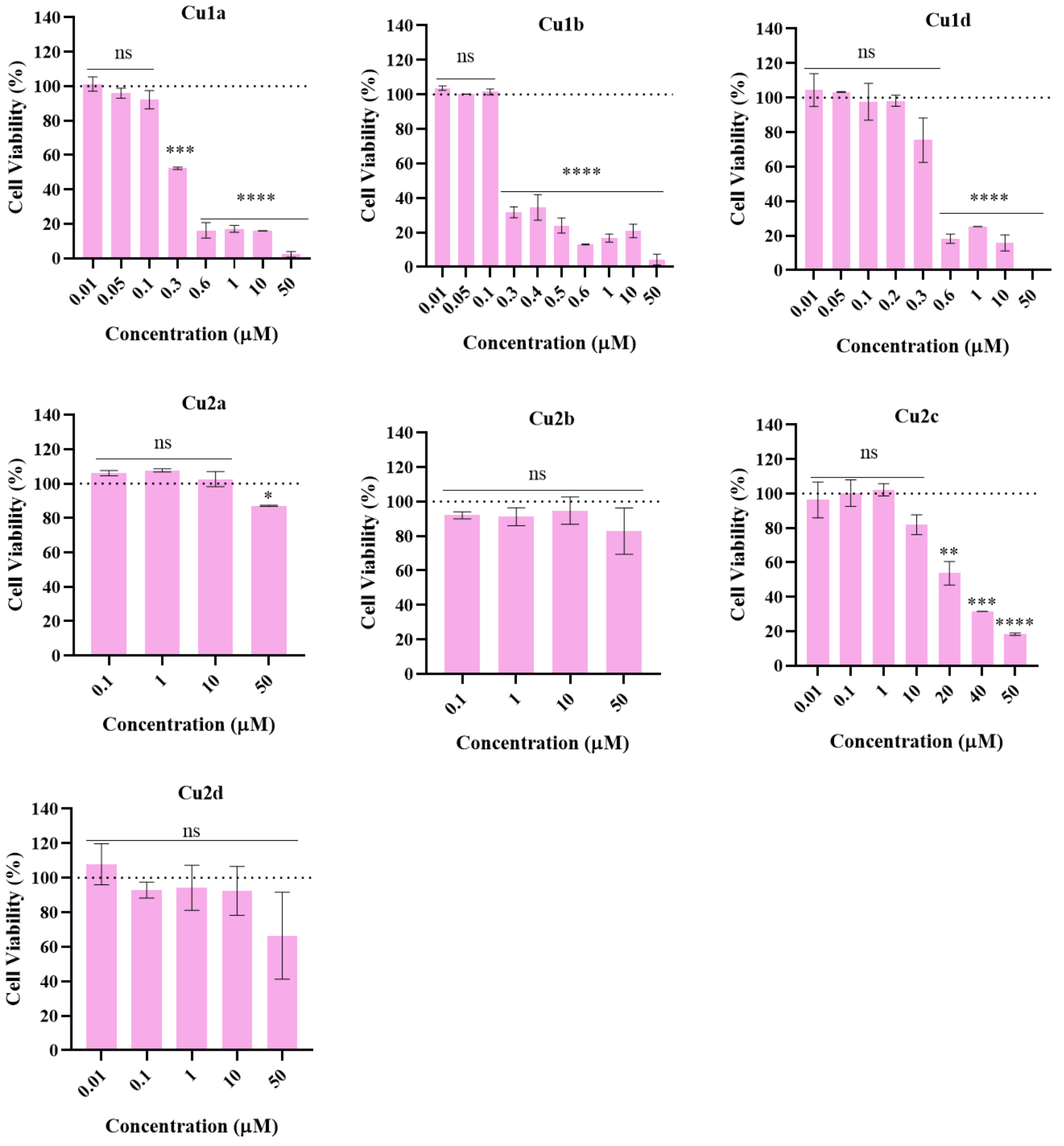

Figure S9. Cell viability of HCT116 tumour cell line after 48 h of exposure to various concentrations of Cu(II) complexes. 0.1% (v/v) DMSO was used as a vehicle control (corresponds to the dot line). The values presented are the mean ± SEM of two independent biological assays, and statistical significance was evaluated in relation to the DMSO control using the Student t-test and One-Way ANOVA (* p-value ≤ 0.05; ** p-value ≤ 0.005; *** p-value ≤ 0.0005; **** p-value ≤ 0.0001).

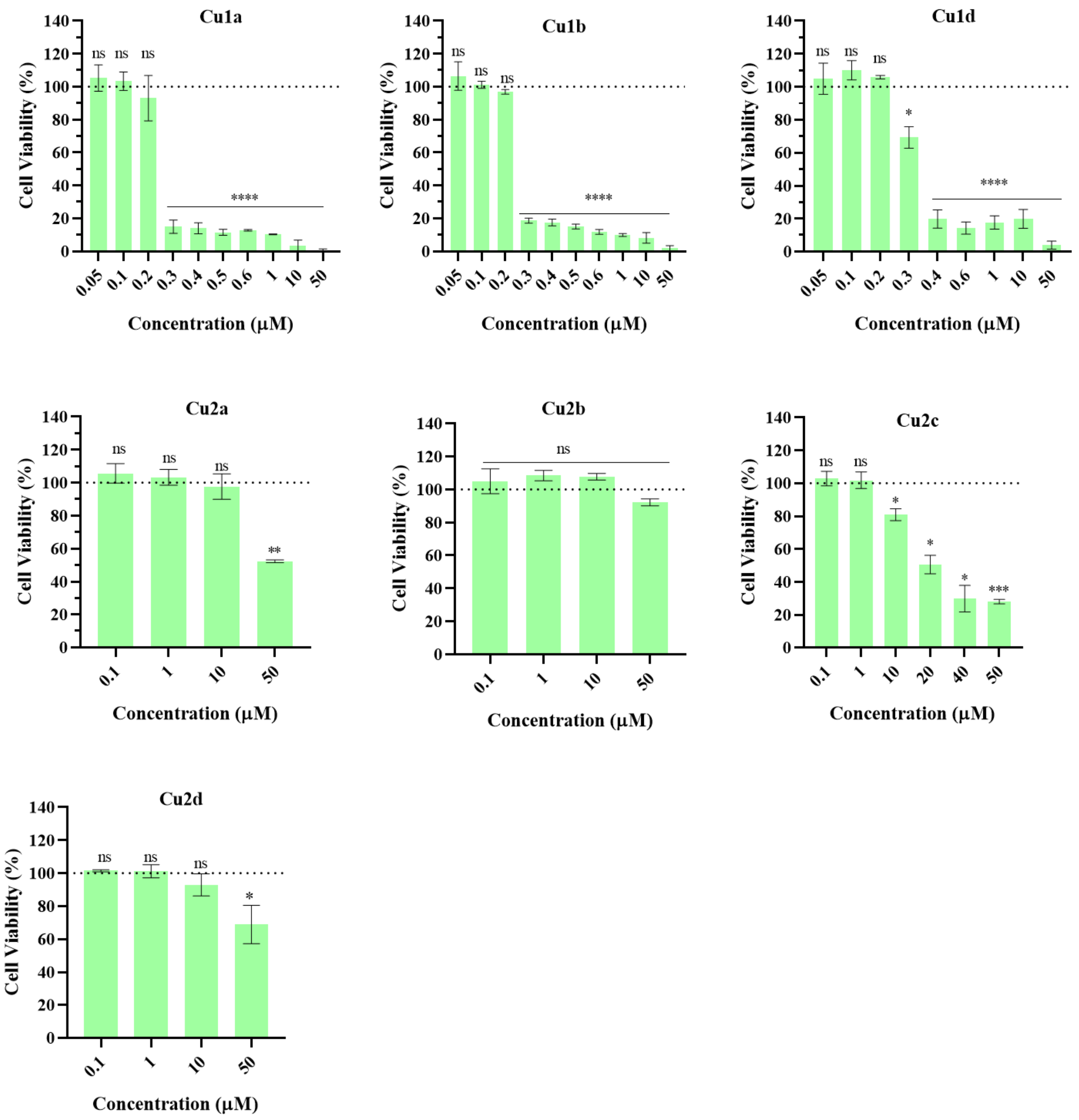

Figure S10. Cell viability of HCT116-DoxR tumour cell line after 48 h of exposure to various concentrations of complexes. 0.1% (v/v) DMSO was used as a vehicle control. The values presented are the mean ± SEM of two independent biological assays, and statistical significance was evaluated in relation to the DMSO control using the Student t-test and One-Way ANOVA (* p-value ≤ 0.05; ** p-value ≤ 0.005; *** p-value ≤ 0.0005; **** p-value ≤ 0.0001).

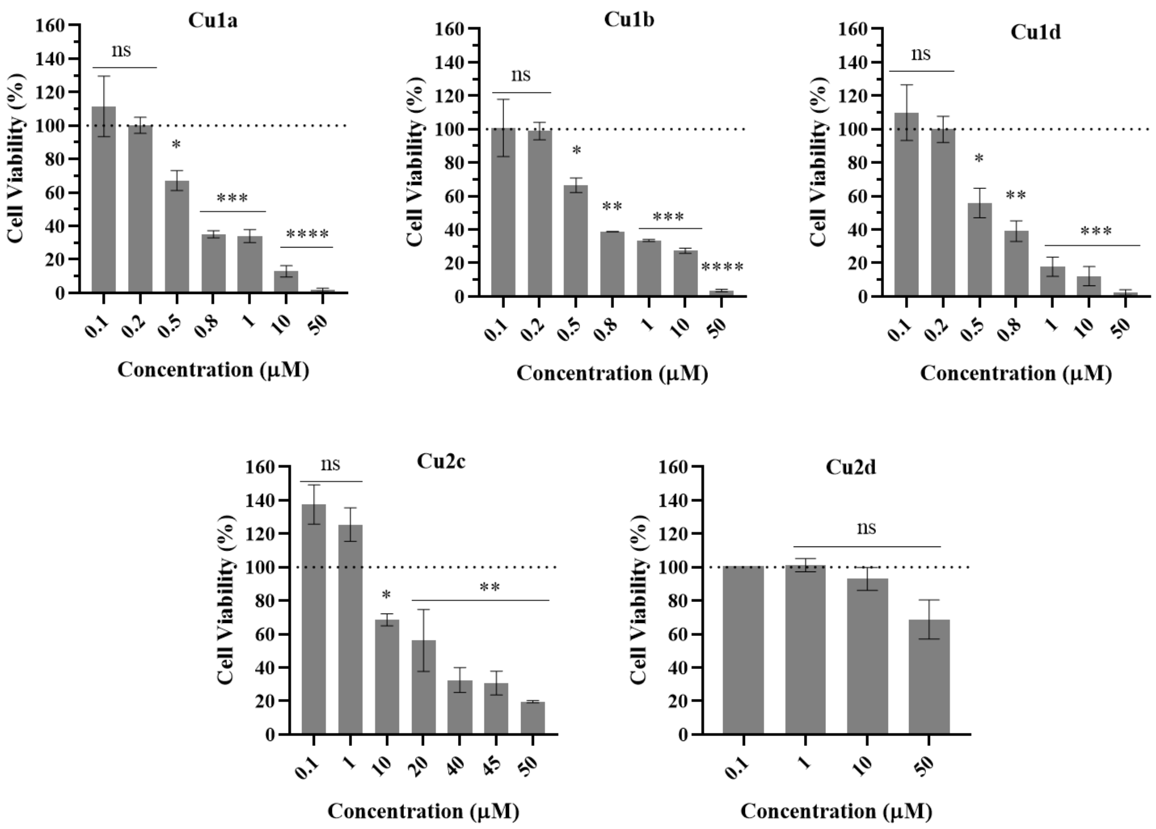

Figure S11. Cell viability of A2780 tumour cell line after 48 h of exposure to various concentrations of complexes. 0.1% (v/v) DMSO was used as a control vehicle. The values presented are the mean ± SEM of two independent biological assays, and statistical significance was evaluated in relation to the DMSO control using the Student t-test and One-Way ANOVA (* p-value ≤ 0.05; ** p-value ≤ 0.005; *** p-value ≤ 0.0005; **** p-value ≤ 0.0001).

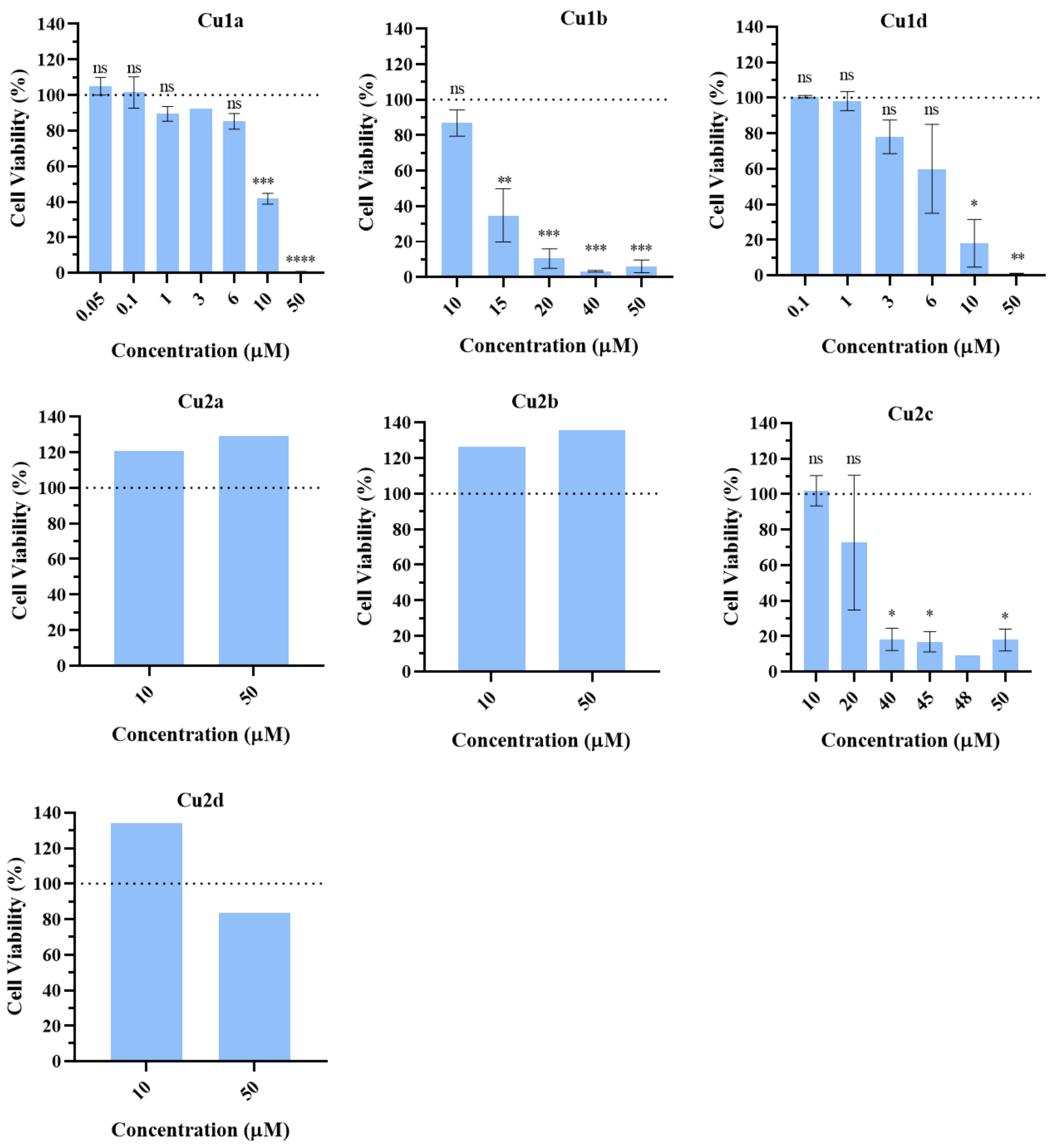

Figure S12. Cell viability of the healthy human Fibroblasts after 48 h of exposure to various concentrations of complexes. 0.1% (v/v) DMSO was used as vehicle control. The values presented are the mean ± SEM of two independent biological assays, and statistical significance was evaluated in relation to the DMSO control using the Student's t-test and One-Way ANOVA (* p-value ≤ 0.05; ** p-value ≤ 0.005; *** p-value ≤ 0.0005; **** p-value ≤ 0.0001).

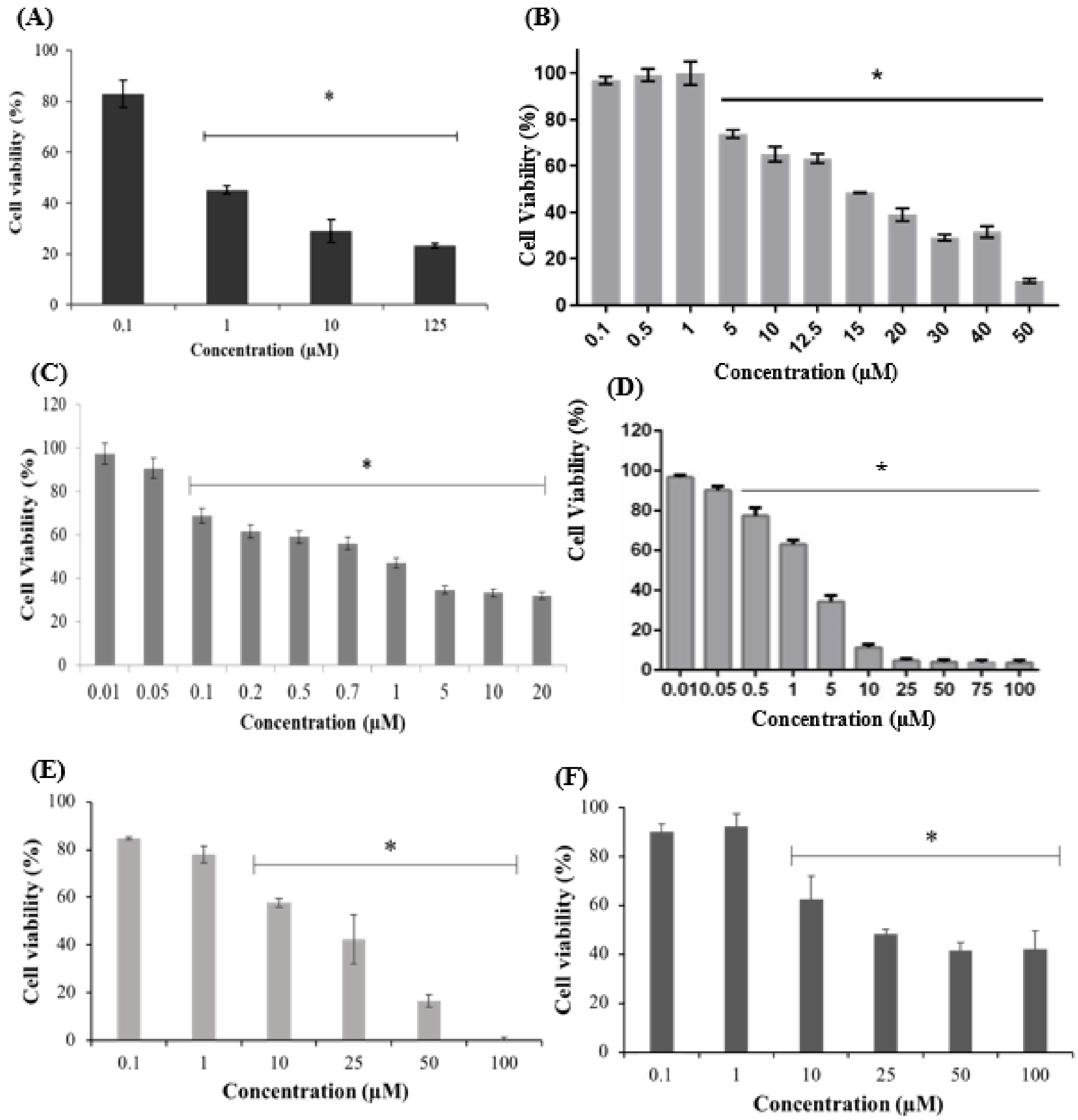

Figure S13. Viability of HCT116 cancer cell line after exposure to different concentrations of doxorubicin (A) and cisplatin (B) for 48 hours. Viability of A2780 cancer cell line after exposure to different concentrations of doxorubicin (C) and cisplatin (D) for 48 hours. Viability of Fibroblasts after exposure to different concentrations of doxorubicin (E) and cisplatin (F). 0.1% (v/v) DMSO was used as the vehicle control. Data are expressed as the mean ± SEM of three biological assays.

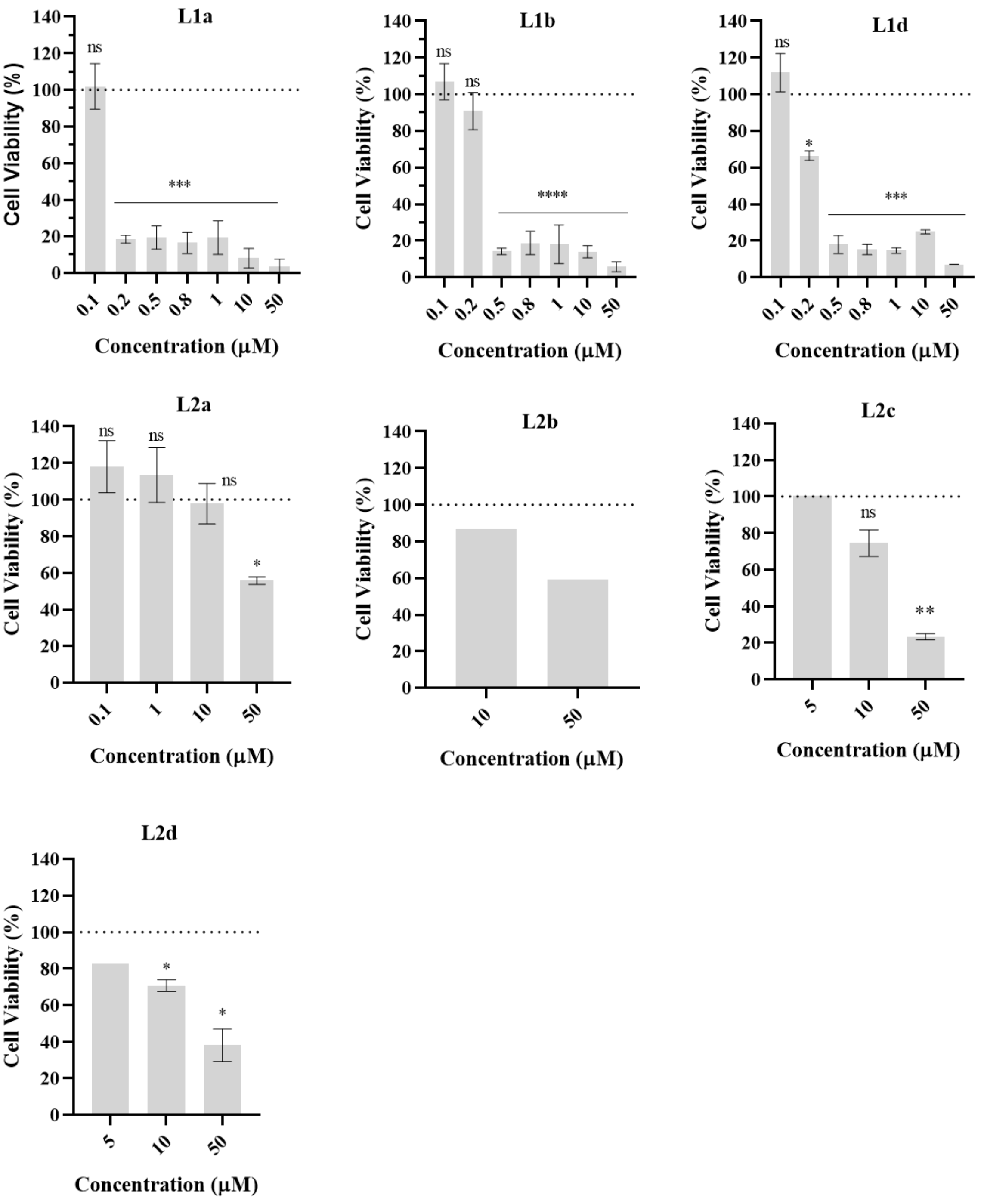

Figure S14. Cell viability of HCT116-DoxR tumour cell line after 48 h of exposure to various concentrations of ligands. 0.1% (v/v)DMSO was used as a vehicle control. The values presented correspond to the mean ± SEM of two independent biological assays, and statistical significance was assessed in relation to the DMSO control using the T-Student method and One-Way ANOVA (* p-value ≤ 0.05; ** p-value ≤ 0.005; *** p-value ≤ 0.0005; **** p-value ≤ 0.0001).

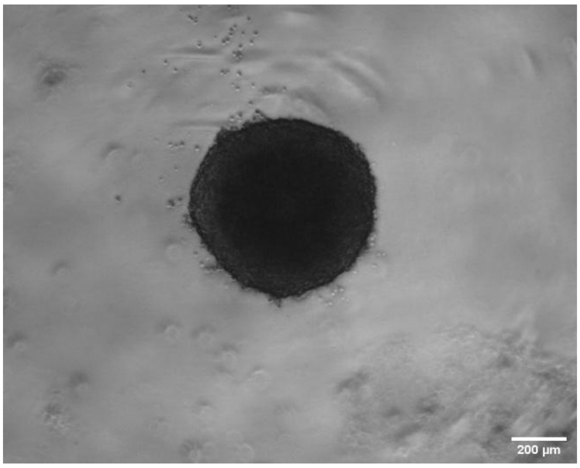

Figure S15. HCT116DoxR spheroid with 6 days of growth. Scale of 200 µm. See also video for spheroid formation till the 6th day of growth.

Video S15. Spheroid formation till the 6th day of growth.

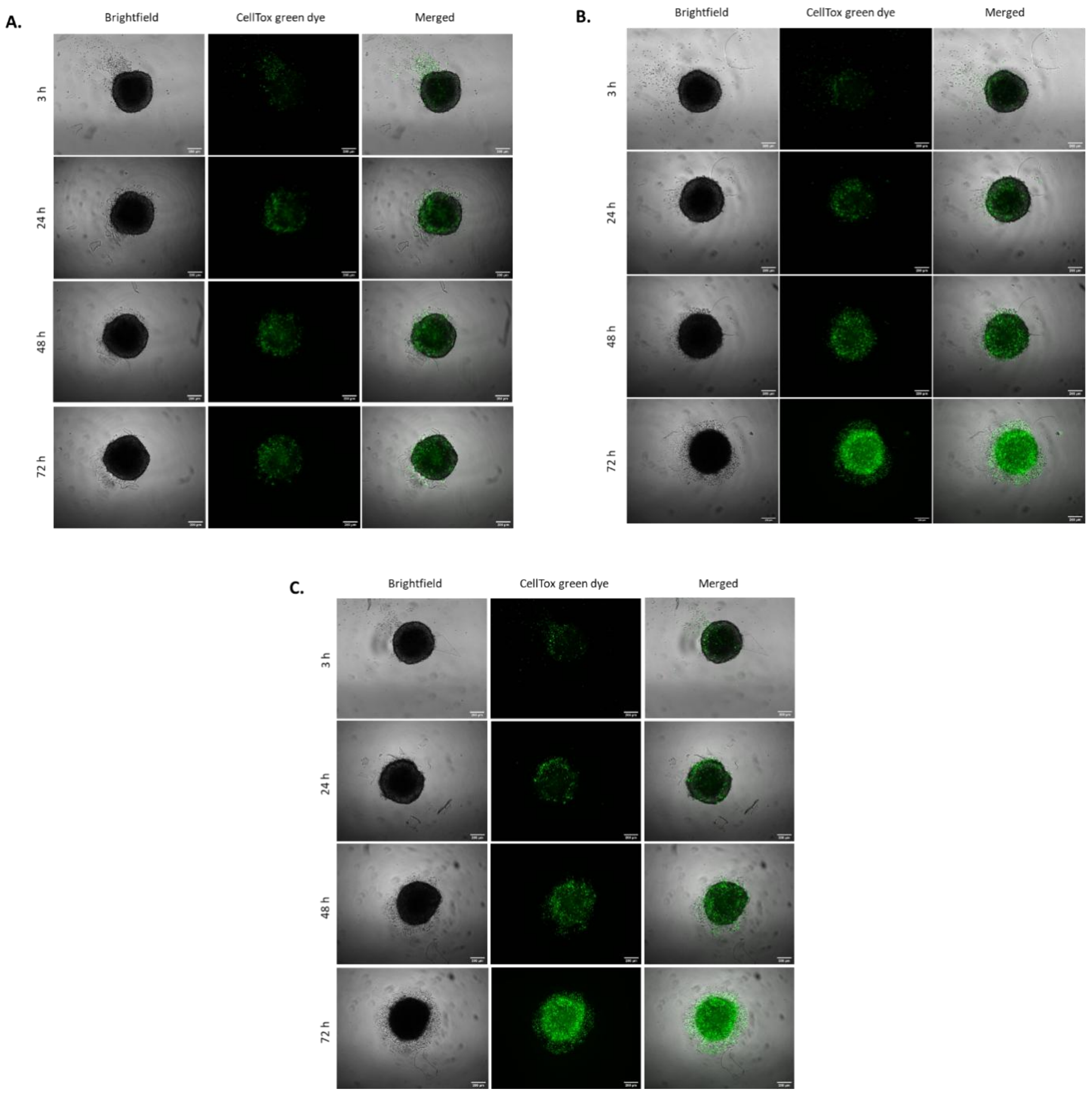

Figure S16. Cell viability in HCT116-DoxR 3D spheroids after exposure to DMSO (vehicle control) or complexes Cu1a and Cu1b and incubation with CellToxTM Green dye for 3, 24, 48 and 72 h. Fluorescence microscopy images of HCT116-DoxR spheroids exposed to (A) DMSO, (B) complex Cu1a and (C) complex Cu1b. Images represent three independent biological assays (each with at least 3 spheroids. Scale of 200 µm.

6. Stability and Solubility of Complexes Cu1a and Cu1b

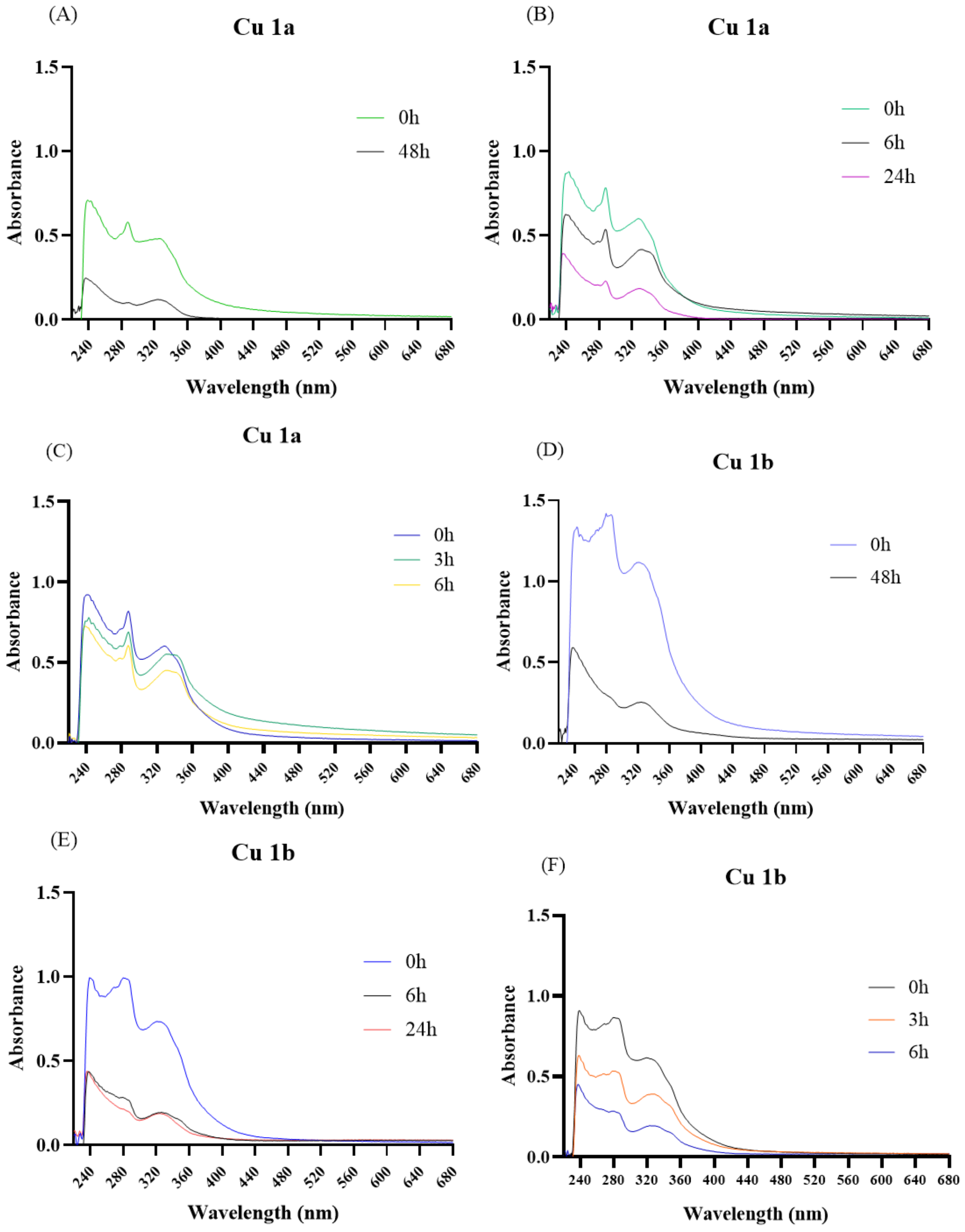

Figure S17. Evaluation of the stability and solubility of complexes Cu1a ((A), (B) and (C)) and Cu1b ((D), (E) and (F)) by UV-Vis spectroscopy. Absorption spectra 50 µM of complexes Cu1a and Cu1b in RPMI culture medium (previously diluted in DMSO) at different incubation times: 0, 3, 6, 24, and 48 h, at 37°C.

7. Western Blot Assay - BAX and BCL-2 Protein Expression

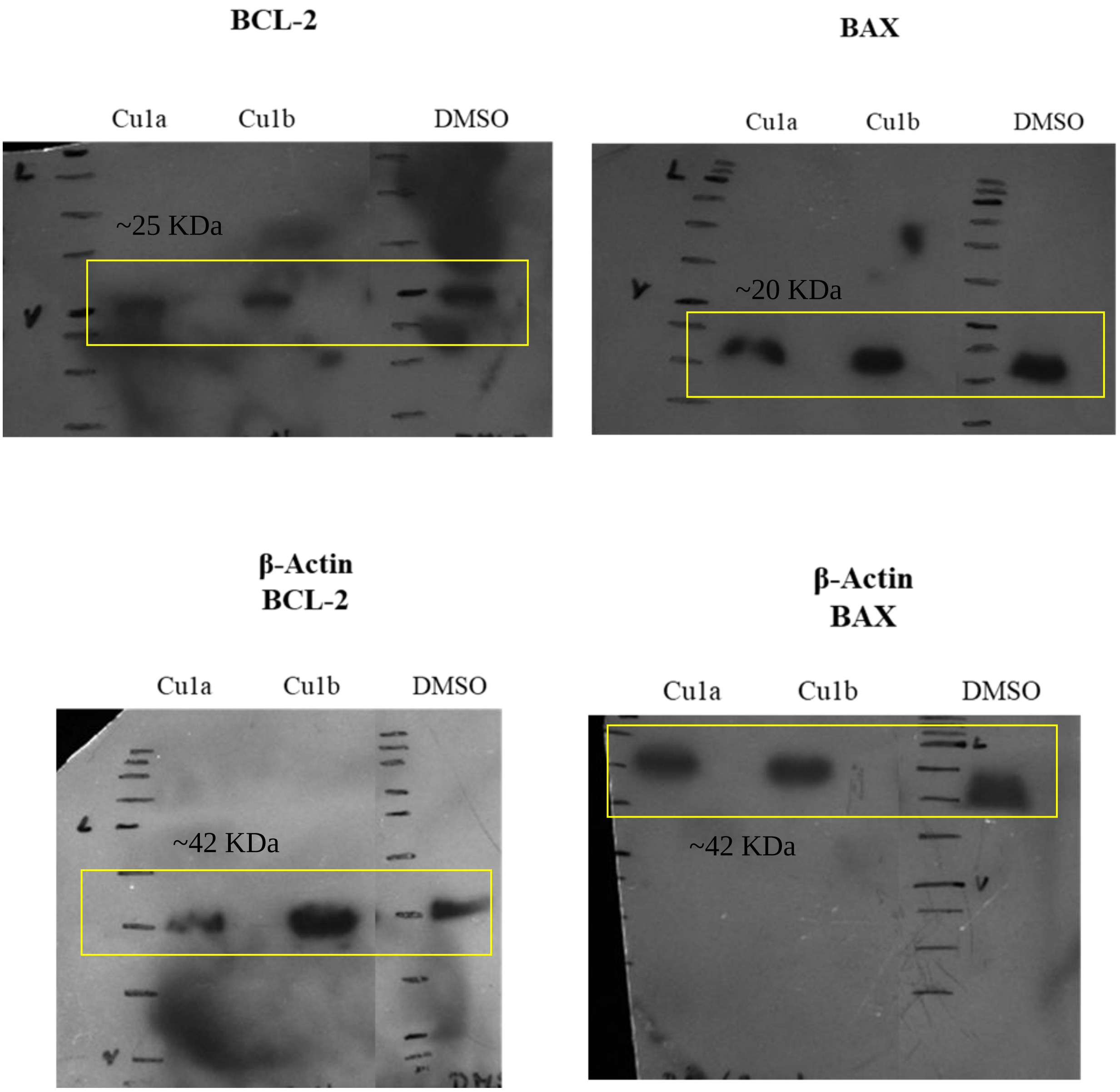

Figure S18. Western Blot bands utilized to quantify the expression of proteins BCL-2 and BAX in HCT116-DoxR cells after exposure to the respective negative control (DMSO) or complexes Cu1a and Cu1b.

8. ctDNA Interaction Studies

Cu1a:

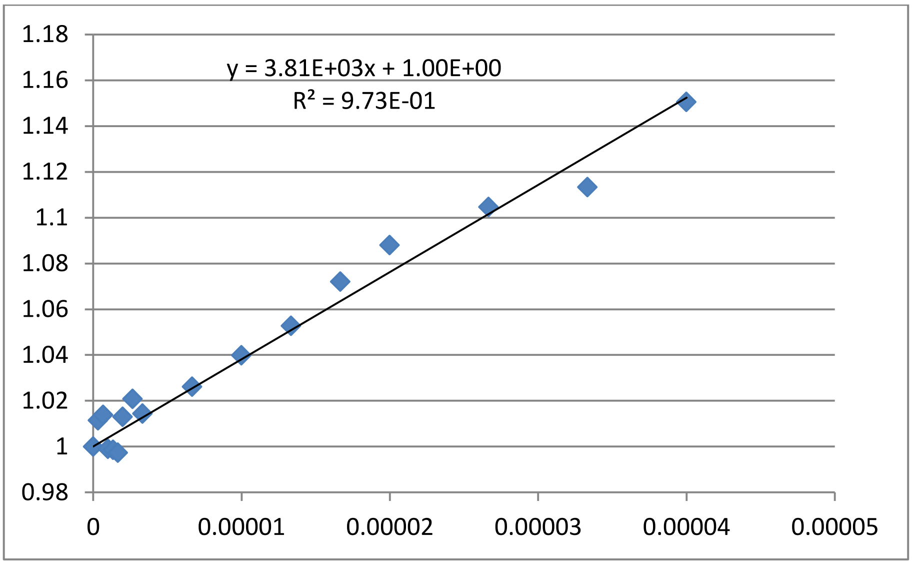

$K_{app} = 1.75 \times 10^5\ M^{-1}$

Figure S19. Stern-Volmer plot for EB-DNA adduct treated with Cu1a

Cu1b:

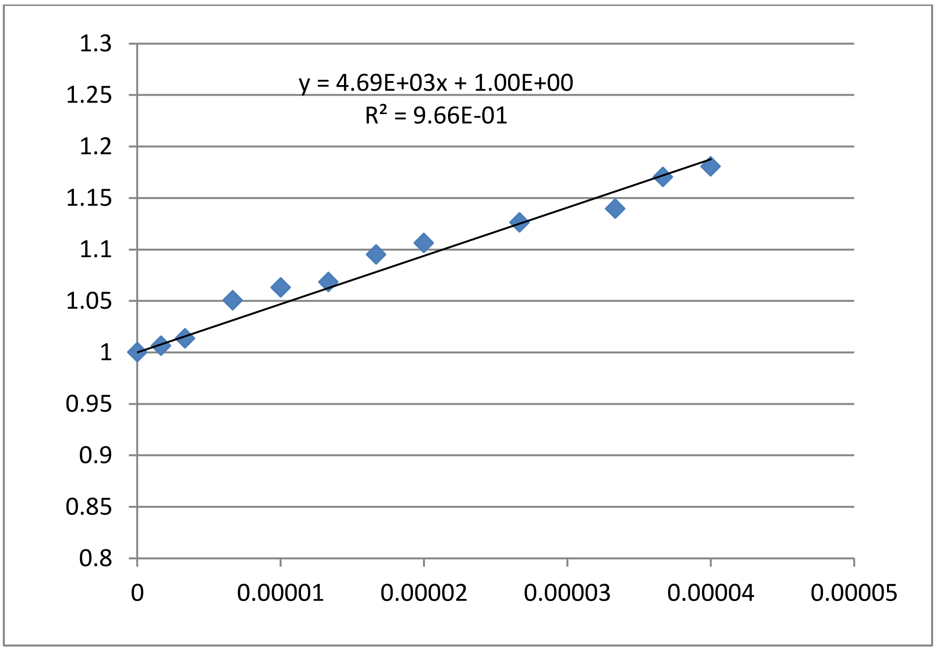

$K_{app} = 2.35 \times 10^6\ M^{-1}$

Figure S20. Stern-Volmer plot for EB-DNA adduct treated with Cu1b

Cu1a:

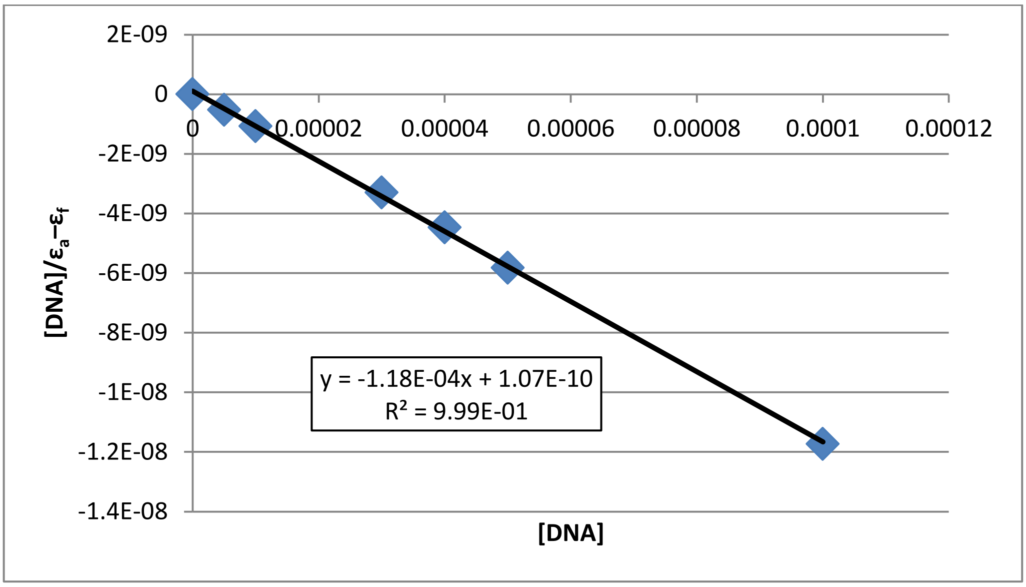

$K_b = 1.10 \times 10^6\ M^{-1}$

Figure S21. Wolfe–Shimmer's plot for Cu1a treated with increased amount of DNA

Cu1b:

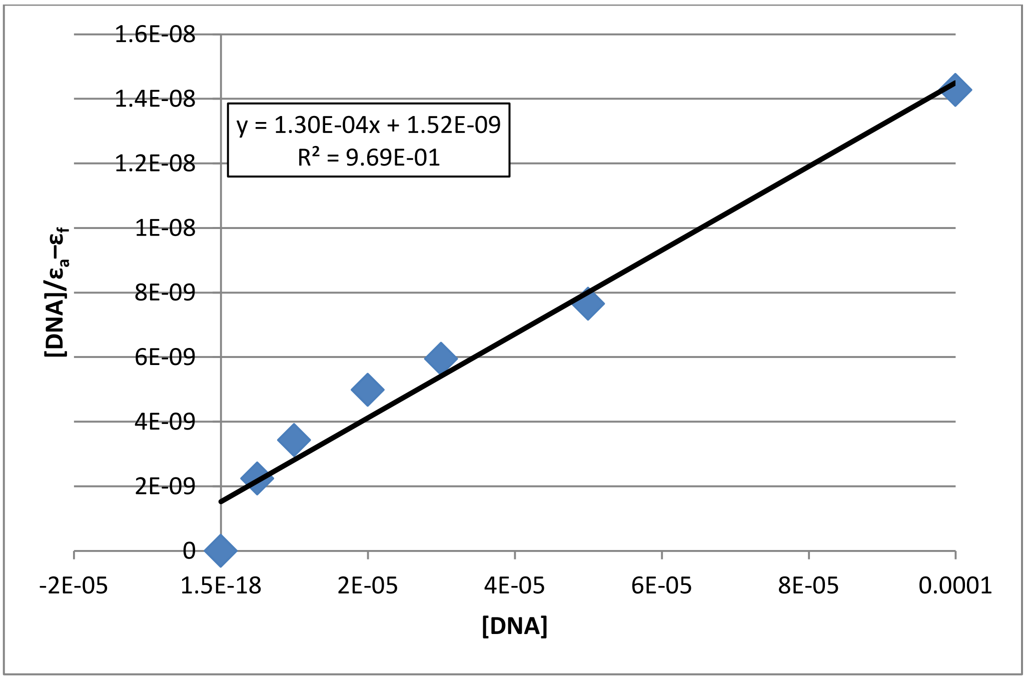

$Kb = 8.85x\ 10^5\ M^{-1}$

Figure S22. Wolfe–Shimmer's plot for Cu1b treated with increased amount of DNA

9. pDNA Cleavage Mechanisms

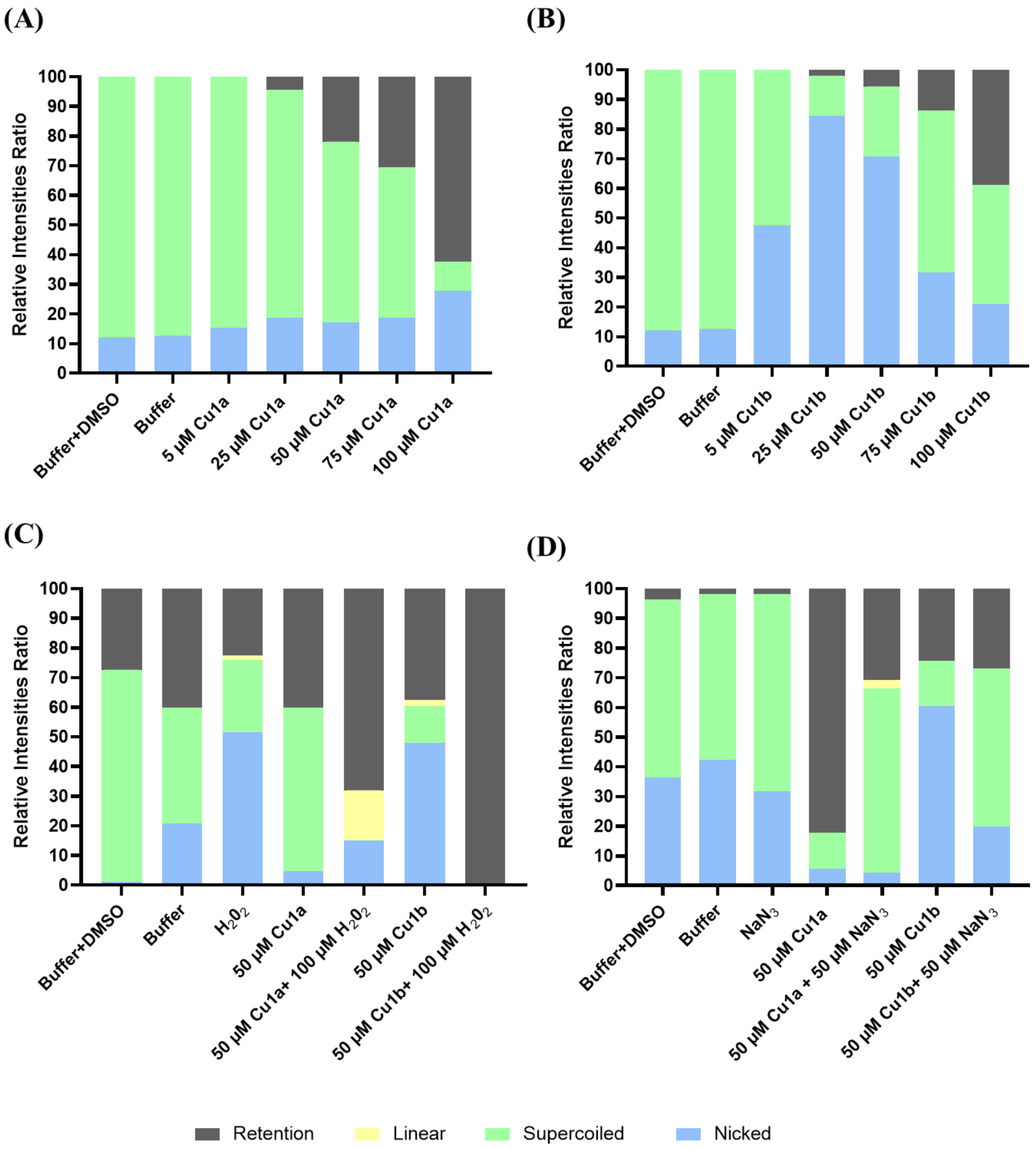

Figure S23. Ratio of the intensities of circular, linear and supercoiled isoforms.(A) and (B) show the quantification of the interaction of Cu(II) complexes with pDNA at increasing concentrations of the complexes (5, 25, 50, 75 and 100 µM).(C) e (D)-quantification of samples subjected to the NaN_3 sequestering agent and the H_2O_2 cleavage positive control, respectively. Incubated at 37 ºC for a period of 24 hours.

10. Fibroblast Cell Migration Assay

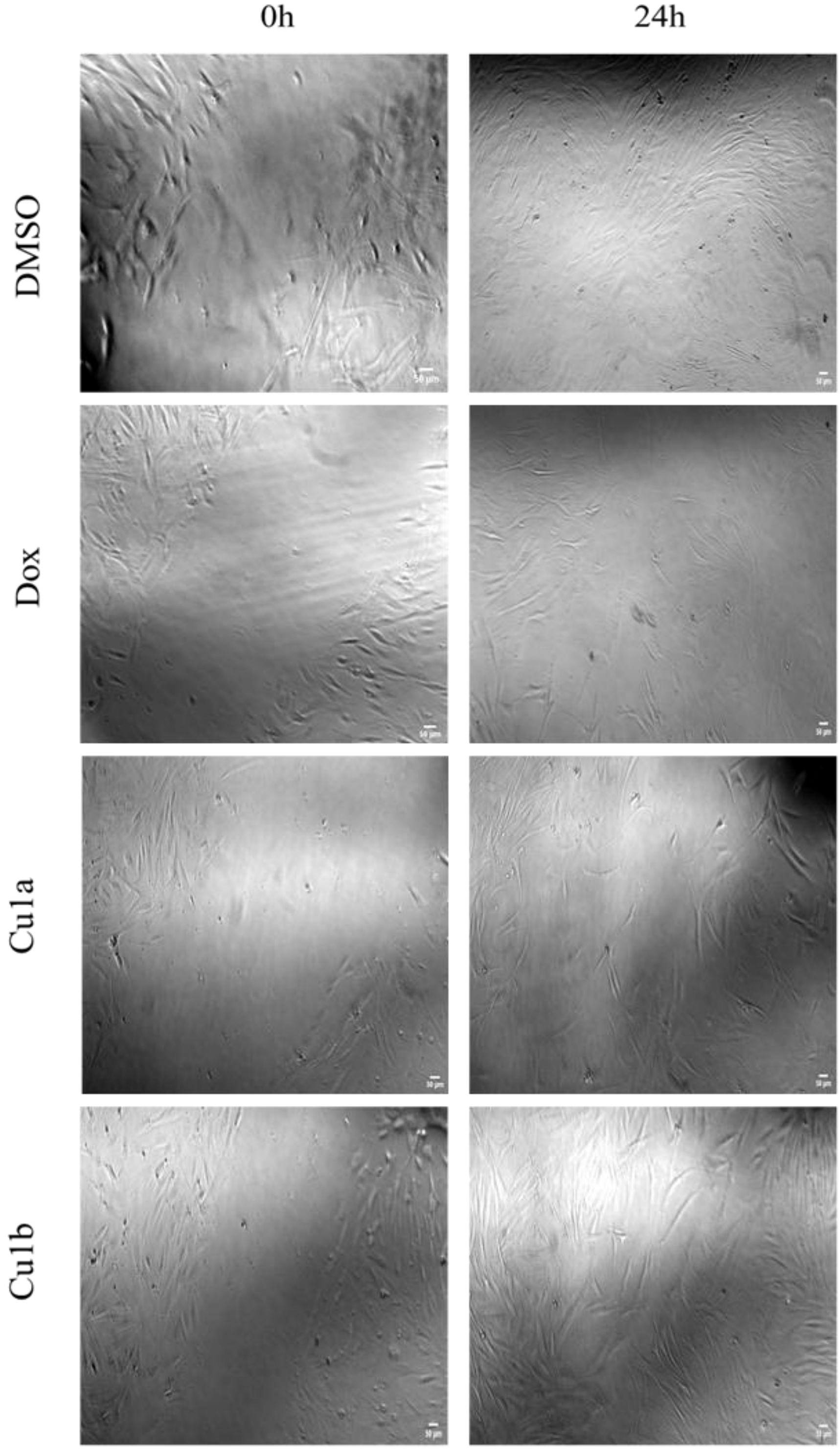

Figure S24. Representative images of the wound-healing assay in fibroblasts after incubation for 0 and 24 h. Inverted microscope with 4x magnification.

11. Evaluation of In-Vivo Angiogenic Potential

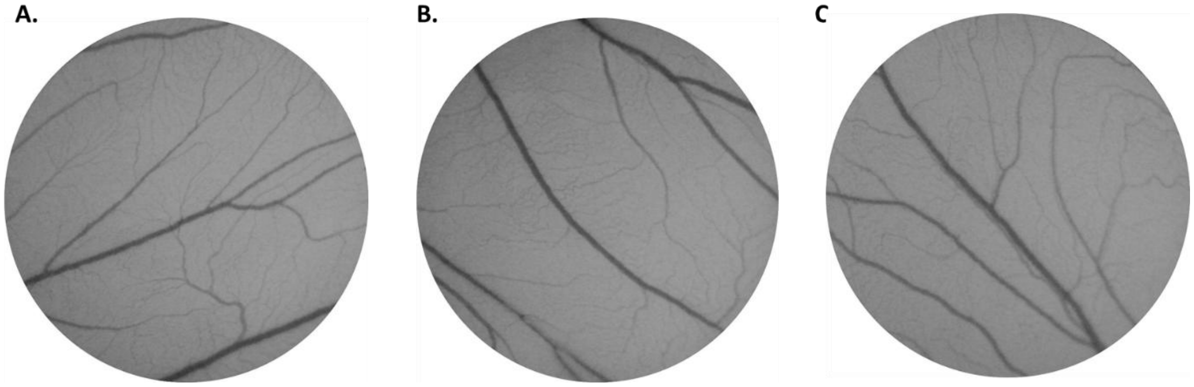

Figure S25. Evaluation of the complexes potentiality to modulate angiogenesis. Green channel of the same image used for counting the number of veins after 24 h exposure to (A) DMSO, (B) IC_{50} concentrations of complex Cu1a and (C) IC_{50}concentrations of complex Cu1b. Inverted microscope with 4x magnification.

12. Interaction with Bovine Serum Albumin (BSA)

In the perspective of translating the complexes into clinical practice and considering their administration and distribution in the human body, it is essential to examine their potential interaction with albumin, a protein present in human plasma at high concentrations[21]. In this regard, bovine serum albumin (BSA) was utilized as a model[22]. BSA is the most abundant plasma protein in vertebrates, sharing approximately 76% similarity with human serum albumin (HSA) and composed of single polypeptide chains with 583 amino acids[21,23].

UV-Visible spectroscopy is an effective method for predicting conformational changes in proteins when interacting with small molecules such as drugs[24]. The interaction of BSA with complexes **Cu1a** and **Cu1b** was characterized using UV-Visible and fluorescence spectroscopy, considering the intrinsic fluorescence of tryptophan residues in BSA. **Figure S26** displays the UV-Visible spectra obtained for BSA, BSA in combination with 0.1% (v/v) DMSO and BSA in the presence of increasing concentrations of the metallic complexes.

Absorbances were measured in a concentration range of 245-500 nm. Aromatic residues in the BSA protein, such as tryptophan, tyrosine, and phenylalanine, exhibit π-π* electronic transitions resulting in maximum absorbance peaks around 280 nm[21,25].

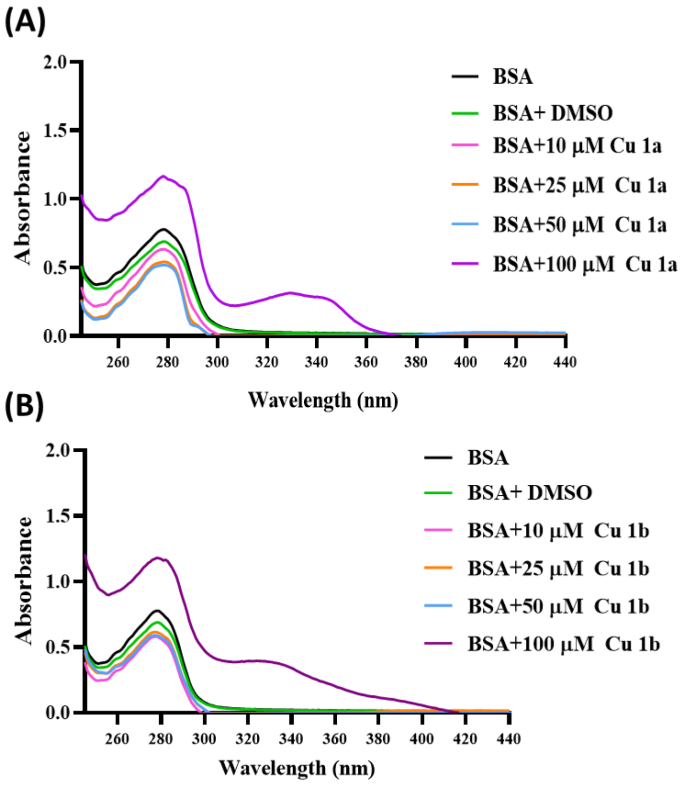

Figure S26. UV-Visible absorption spectra depict the interaction between 20 µM of BSA exposed to variable concentrations of complexes (A) Cu1a and (B) Cu1b. Different interaction ratios were observed after a 24 h incubation period at 37 °C. The spectrum is normalized concerning the absorbance of the individual BSA sample to facilitate the interpretation of the interaction effects.

The absorption spectra (**Figure S26**) reveal that complexes **Cu1a** and **Cu1b** exhibited absorbance peaks around 280 nm, corresponding to the absorption of aromatic residues in BSA.

When BSA was incubated with **Cu1a** and **Cu1b** (10, 25, and 50 µM) a decrease in the BSA maximum absorbance is observed (hypochromism)[26]. These results indicate an interaction between the complexes and BSA, as the decrease in light absorption by BSA suggests

changes in the structure and environment that may affect the proteins' ability to absorb light at specific wavelengths. It could involve blocking chromophore groups or conformational changes that hinder this activity[27].

For higher concentrations of the complexes (100 μM) new minor absorbance peaks emerged at different wavelengths: 310-360 nm for complex **Cu1a**; 310-360 nm for complex **Cu1b** and a strong increase in absorbance is observed.

To further confirm these results and the interaction between **Cu1a** or **Cu1b** and BSA, fluorescence spectroscopy was used. Fluorescence spectroscopy is particularly sensitive in detecting conformational changes in the protein. The intrinsic fluorescence of BSA is exclusively derived from tryptophan (Trp) residues since phenylalanine (Phe) has a relatively low quantum yield, and tyrosine (Tyr) residues virtually exhibit no fluorescence[24,28].

In **Figure S27**, it is shown the fluorescence spectra obtained for BSA and BSA conjugated with increasing concentrations of the complexes **Cu1a** and **Cu1b**. It is evident that the results show a decrease in fluorescence intensity, known as the quenching effect or hypochromism of BSA in the presence of increasing concentrations of the complexes. This quenching effect can be interpreted as an indication that the complexes are interacting with BSA, causing changes in the environment around the tryptophan residues that affect its intrinsic fluorescence. Interestingly, when **Cu1a** was incubated with BSA at its maximum concentration, a complete attenuation of BSA fluorescence was observed (**Figure S27-A)**. This may indicate a complete saturation of all available binding sites on BSA, suppressing its emitted fluorescence. Concerning **Cu1b** for all the tested concentration an increased quenching effect was observed without a saturation (**Figure S27-B**).

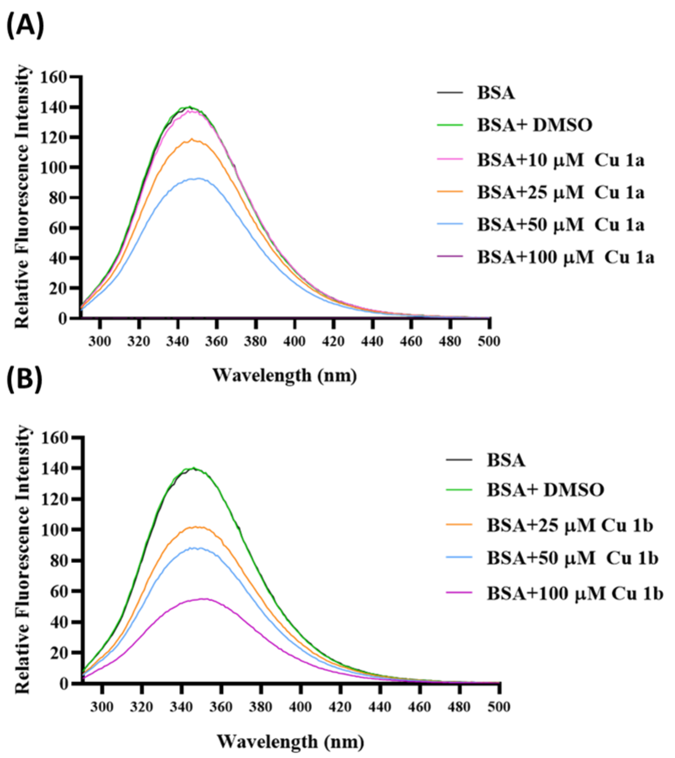

Figure S27. The fluorescence intensity spectrum represents the interaction between BSA and the complexes Cu1a and Cu1b. The BSA concentration is fixed at 20 µM, while the complex concentrations vary between 10-100 µM. The assays were conducted after a 24 h incubation at 37 °C. This spectrum is normalized based on the fluorescence of the individual protein sample to facilitate the interpretation of the interaction's influence.

As in cell-based assays culture medium contain 10% (v/v) FBS, and BSA is a major component of FBS, this interaction may indicate that **Cu1a** and C**u1b** may be internalized in cells with albumin, which is in line with the internalization results (**Table 6**) and mediated by an active process. Also, when translating to more complex *in vivo* systems, these interactions are crucial as they may suggest that the complexes could use human serum albumin as a carrier, allowing better distribution and enhancing their therapeutic activity.

References

[1] Y.-Q. Gu, Y.-J. Zhong, M.-Q. Hu, H.-Q. Li, K. Yang, Q. Dong, H. Liang, Z.-F. Chen, *Dalton Trans.*, 2022, 51, 1968–1978

[2] J.-W. Liang, Y. Wang, K.-J. Du, G.-Y. Li, R.-L. Guan, L.-N. Ji, H. Chao, *J. Inorg. Biochem.*, 2014, 141, 17–27

[3] K. Ni, N.Montesdeoca, J.Karges; Dalton Trans., 2024, 53, 8223

[4] J. Li, H. Yan, Z. Wang, R. Liu, B. Luo, D. Yang, H. Chen, L. Pan, Z. Ma, *Dalton Trans.*, 2021, 50, 8243–8257

[5] A. Maroń, K. Czerwińska, B. Machura, L. Raposo, C. Roma-Rodrigues, A. R. Fernandes, J. G. Małecki, A. Szlapa-Kula, S. Kula, S. Krompiec, *Dalton Trans.*, 2018, 47, 6444–6463

[6] K. Velugula, A. Kumar, J. P. Chinta, *Inorg. Chim. Acta* 2020, 507, 119596

[7] J. Grau, A. Caubet, O. Roubeau, D. Montpeyó, J. Lorenzo, P. Gamez. *ChemBioChem* 2020, 21, 2348–2355

[8] S. Wang, W. Chu , Y. Wang, S. Liu , J. Zhang, S. Li, H. Wei, G. Zhou, X. Qin, *Appl. Organometal. Chem.*, 2013, 27, 373–379

[9] K. Suntharalingam, D. J. Hunt, A. A. Duarte, A. J. P. White, D. J. Mann, R. Vilar, *Chem. Eur. J.* 2012, 18, 15133–15141

[10] K. Czerwińska, B. Machura, S. Kula, S. Krompiec, K. Erfurt, C. Rodrigues, A. Fernandes, L.S. Shul'pina, N.S. Ikonnikov, G.B. Shul'pin, *Dalton Transactions*, 46, 2017, 9591–9604

[11] K. Choroba, B. Machura, S. Kula, L. R. Raposo, A. R. Fernandes, R. Kruszynski, K. Erfurt, L. S. Shul'pina, Y. N. Kozlov, G. B. Shul'pin, *Dalton Transactions*, 2019, 48, 12656–12673

[12] D.-Y. Zhang, Y. Nie, H. Sang, J.-J. Suo, Z.-J. Li, W. Gu, J.-L. Tian, X. Liu, S.-P. Yan,*InorganicaChimicaActa*, 2017, 457, 7–18

[13] Y. Yang, F.-F. Guo, C.-F. Chen, Y.-L. Li, H. Liang, Z.-F. Chen,*Journal of Inorganic Biochemistry*, 2023, 240, 112093

[14] Y. Yang, C.-F. Chen, F.-F. Guo, Y.-Q. Gu, H. Liang, Z.-F. Chen, *Journal of Inorganic Biochemistry*, 2023, 246, 112284

[15] V. M. Manikandamathavan, V. Rajapandian, A. J. Freddy, T. Weyhermüller, V. Subramanian, B. U. Nair, *Eur. J. Med. Chem.*, 2012, 57, 449–458

[16] T. Rojo, M. Vlasse, D. Beltran-Porter, *Acta Cryst. C*, 1983, 39, 194–199

[17] G.-Y. Li, K.-J. Du, J.-Q. Wang, J.-W. Liang, J.-F. Kou, X.-J. Hou, L.-N. Ji, H. Chao, *J. Inorg. Biochem.*, 2013, 119, 43–53

[18] Z. Ma, L. Wei, E. C. B. A. Alegria, L. M. D. R. S. Martins, M. F. C. G. da Silva, A. J. L. Pombeiro, *Dalton Trans.*, 2014, 43, 4048–4058

[19] T.-H. Huang, M.-H. Zhang, C.-Y. Gao, L.-T. Wang, *Inorg. Chim. Acta*, 2013, 408, 91–95

[20] L. Li, K. Du, Y. Wang, H. Jia, X. Hou, H. Chao, L. Ji, *Dalton Trans.*, 2013, 42, 11576–11588

[21] J.H. Shi, K.L. Zhou, Y.Y. Lou, D.Q. Pan, *SpectrochimicaActa - Part A: Molecular and Biomolecular Spectroscopy* 2018, 188, 362–371

[22] T. Topală, A. Bodoki, L. Oprean, R. Oprean, *Clujul Medical*, 2014, 87, 5

[23] P. Sengupta, U. Pal, P. Roy, T. Samanta, N. Chattopadhyay, K. Sen, A. Bose, *ACS Food Science and Technology*, 2022, 2, 114–124

[24] J. Liu, Y. He, D. Liu, Y. He, Z. Tang, H. Lou, Y. Huo, X. Cao, *RSC Advances*, 2018, 8, 7280–7286

[25] Y.-Y. Lou, K.-L. Zhou, D.-Q. Pan, J.-L. Shen, J.-H. Shi, *Journal of Photochemistry and Photobiology B: Biology*, 2017, 167, 158–167.

[26] S. Wongsuwan, J. Chatwichien, W. Sirisaksoontorn, K. Chainok, A. Songsasen, R. Chotima, *New Journal of Chemistry*, 2023, 47, 10624–10637

[27] A. Pan, I. Mitra, S. Mukherjee, S. Ghosh, U. Chatterji, S.C. Moi, *ACS Applied Bio Materials*, 2021, 4, 853–868

[28] H. Xu, N. Yao, H. Xu, T. Wang, G. Li, Z. Li, *International Journal of Molecular Sciences*, 2013, 14, 14185–14203

Abbreviations

A2780 – ovarian endometrioid adenocarcinoma

A549 –lung adenocarcinoma

Bel-7402 –hepatocellular carcinoma

BGC-823 – gastrocarcinoma

CT-26 – mouse colorectal carcinoma

Eca-109 –esophageal squamous carcinoma

GM-5657 – non-cancerous human fibroblasts

HCT116 – colon carcinoma

HeLa –cervix carcinoma

HepG2 – hepatoblastoma

HL-60 – immature granulocyte leukemia

HL-7702 – human papillomavirus-related endocervical adenocarcinoma

HT-29 – colon adenocarcinoma

KB – nasopharyngeal carcinoma

MCF-7 –invasive breast carcinoma

MDA-MB-231 –human breast carcinoma

MGC80-3 –gastric mucinous adenocarcinoma

NCI-H460–lung large cell carcinoma

SK-OV-3 – ovarian serous cystadenocarcinoma

T-24 – bladder carcinoma
